# Horizontal operon transfer, plasmids, and the evolution of photosynthesis in *Rhodobacteraceae*

**DOI:** 10.1038/s41396-018-0150-9

**Published:** 2018-05-24

**Authors:** Henner Brinkmann, Markus Göker, Michal Koblížek, Irene Wagner-Döbler, Jörn Petersen

**Affiliations:** 1 Department of Protists and Cyanobacteria (PuC), Leibniz-Institute DSMZ–German Collection of Microorganisms and Cell Cultures, Braunschweig, Germany; 2 Department of Bioinformatics, Leibniz-Institute DSMZ–German Collection of Microorganisms and Cell Cultures, Braunschweig, Germany; 3 Laboratory of Anoxygenic Phototrophs, Institute of Microbiology, CAS, Center Algatech, Trebon, Czech Republic; 4 Research Group Microbial Communication, Helmholtz Centre for Infection Research, Braunschweig, Germany

## Abstract

The capacity for anoxygenic photosynthesis is scattered throughout the phylogeny of the *Proteobacteria*. Their photosynthesis genes are typically located in a so-called photosynthesis gene cluster (PGC). It is unclear (i) whether phototrophy is an ancestral trait that was frequently lost or (ii) whether it was acquired later by horizontal gene transfer. We investigated the evolution of phototrophy in 105 genome-sequenced *Rhodobacteraceae* and provide the first unequivocal evidence for the horizontal transfer of the PGC. The 33 concatenated core genes of the PGC formed a robust phylogenetic tree and the comparison with single-gene trees demonstrated the dominance of joint evolution. The PGC tree is, however, largely incongruent with the species tree and at least seven transfers of the PGC are required to reconcile both phylogenies. The origin of a derived branch containing the PGC of the model organism *Rhodobacter capsulatus* correlates with a diagnostic gene replacement of *pufC* by *pufX*. The PGC is located on plasmids in six of the analyzed genomes and its DnaA-like replication module was discovered at a conserved central position of the PGC. A scenario of plasmid-borne horizontal transfer of the PGC and its reintegration into the chromosome could explain the current distribution of phototrophy in *Rhodobacteraceae*.

## Introduction

Life on this planet originated in an anoxygenic environment and therefore early microbial evolution was the age of anaerobes [1]. Cells obtained their energy from transfer of electrons, e.g., from inorganic H_2_ to inorganic acceptors such as CO_2_ or elementary sulfur, a mode of growth that is termed litho-autotrophy [2]. A huge breakthrough was the evolution of the ability to generate energy from light. Photosynthesis (PS) represents one of the most important biological processes and the first photosynthetic organisms evolved under anoxic conditions during the Archaean period about 3.5 billion years (Gyr) ago [3]. The earliest phototrophs conducted anoxygenic PS without the release of oxygen. The evolution of oxygenic PS in cyanobacteria started to oxygenate the earth about 2.4 Gyr ago [4]. Today, molecular oxygen is the most abundant electron acceptor for the production of biochemical energy from biomass and fuel respiration in eukaryotes as well as heterotrophic growth of many aerobic bacteria. How oxygenic PS in cyanobacteria evolved from its anoxygenic ancestor is one of the major unsolved questions in evolution [5].

Today, anoxygenic PS is found in six bacterial phyla: *Proteobacteria* (*Alphaproteobacteria*, *Betaproteobacteria*, and *Gammaproteobacteria*), *Chlorobi*, *Chloroflexi*, *Firmicutes*, *Acidobacteria*, and *Gemmatimonadetes* [5, 6]. The primary photosynthetic reaction is catalyzed by two functionally different types of reaction centers (RCs), of which RC1 may be the ancient type. Phototrophic *Firmicutes* (*Heliobacteria*), *Chlorobi*, and *Acidobacteria* utilize the iron sulfur-containing RC1 type, while *Proteobacteria*, *Gemmatimonadetes*, and *Chloroflexi* possess the RC2 that uses quinone electron acceptors [3]. Oxygenic cyanobacteria harbor both RC types that work in concert to bridge the large difference of the redox potential between water and NADP^+^. Sequence analyses resulted in multiple proposals for the origin and evolution of phototrophy, with the role of horizontal gene transfer (HGT) being a major distinction between them [7–11]. Based on comparative physiological analyses, it was suggested that anoxygenic PS originated in protocyanobacteria and later evolved into oxygenic PS [5]. This would imply that the photosynthetic capacity entered *Proteobacteria* by HGT, a hypothesis that is in contrast to the earlier assumption that the ancestor of *Proteobacteria* performed anoxygenic PS and this ability was lost in some extant taxa [12–14].

The genes for anoxygenic PS of *Alphaproteobacteria* are clustered in a characteristic ensemble of operons, the so-called photosynthesis gene cluster (PGC), which has also been identified in photosynthetic *Betaproteobacteria* and *Gammaproteobacteria* [15, 16]. The PGC is a continuous stretch of about 40 genes encoding all proteins of the PS RC, enzymes of the bacteriochlorophyll and carotenoid biosynthetic pathways, regulatory proteins, and cofactors. Such a compact organization of all PS genes in one continuous cluster is noteworthy. It has been suggested that this clustering may facilitate the horizontal transfer [17]. This mechanism was also proposed to explain the presence of PGCs in *Rubrivivax gelatinosus* or (*Proteobacteria*) and *Gemmatimonas phototrophica* (*Gemmatimonadetes*) [18, 19].

The PGC has a patchy distribution along the phylogenetic tree of *Proteobacteria*. Phototrophic species are often closely related to non-photosynthetic ones depending on respiration, fermentation, or denitrification. Moreover, in some extant species of the *Roseobacter* group (*Rhodobacteraceae*) anoxygenic PS is performed in the presence of oxygen [20]. This type of physiology is termed aerobic anoxygenic photosynthesis (AAnP), and as far as we currently know, it is not able to sustain photoautotrophic growth. AAnP species are photo-heterotrophic, i.e., they are dependent on biomass for growth and use light as an additional source of energy. They lack the Calvin cycle-specific enzymes ribulose-1,5-bisphosphate carboxylase/oxygenase (Rubisco) and phosphoribulokinase (PRK), which are therefore used as a proxy to differentiate photo-autotrophic from photo-heterotrophic species. However, it was recently shown that *Dinoroseobacter shibae* uses an alternative pathway for CO_2_ fixation, namely, the Ethylmalonyl–CoA pathway [21].

The family *Rhodobacteraceae* is one of the most intensively studied groups of *Proteobacteria* [20, 22, 23]. Two competing alternatives are discussed regarding the patchy distribution of PS within this lineage: (i) The common ancestor of the *Rhodobacteraceae* was phototrophic and some lineages lost the PGC (regressive evolution model; Koblížek et al. 2013). (ii) The ancestor of *Rhodobacteraceae* was heterotrophic and PS was acquired via horizontal PGC transfer (horizontal transfer model). Single-gene phylogenies often do not provide sufficient resolution to differentiate between both explanations and sporadic HGTs of single genes might moreover result in misleading conclusions. However, the second hypothesis is supported by the discovery of the complete PGCs on plasmids in *Roseobacter litoralis* and *Sulfitobacter guttiformis* [24, 25], two representatives of the *Roseobacter* group. Their PS plasmids are stably maintained by replication systems of the compatibility groups DnaA-like I and RepB-III [26], which suggested two independent PGC transfers from the chromosome. This “chromosomal outsourcing” of the complete PGC with all essential genes for PS is the first step of a horizontal transfer scenario that was proposed for roseobacters [27].

Here we address the relative contribution of vertical evolution and HGT in the evolution of phototrophy in *Rhodobacteraceae*. The scattered occurrence of the PGC can be explained (i) by a common photosynthetic origin and multiple independent losses of the PGC (regressive evolution), (ii) by a heterotrophic origin and PGC acquisition by horizontal operon transfer (HOT), or (iii) by a combination of HOT, vertical evolution, and loss. The first explanation, which served as our null hypothesis, is in agreement with the common assumption that the anoxygenic PS is an ancient trait of *Rhodobacteraceae* [14]. In order to decide between these three scenarios, we concatenated the orthologous proteins from the PGCs of 44 *Rhodobacteraceae*, compared this “PS tree” with the phylogenomic “species tree” based on the same set of strains and state-of-the-art reconciliation methods and could thus pinpoint authentic HOTs. Genome analysis revealed that the PGC is located on plasmids in four additional and distantly related *Rhodobacteraceae*, which suggests that extrachromosomal elements played an important role in the evolution of PS of this group.

## Methods

A phylogenomic species tree of 105 representative *Rhodobacteraceae* was calculated to document the distribution of 44 photosynthetic and 61 non-photosynthetic strains. The major aim was the inference of a robust phylogenetic PGC tree by the concatenation of all conserved PGC genes. Comparisons of single-gene phylogenies with the PGC tree were performed to determine the extent of HGT. The comparison of the PGC tree with the species tree, which was calculated on the same set of 44 photosynthetic *Rhodobacteraceae*, provided the basis for a reliable differentiation between a strictly vertical evolution and HOT of the complete PGC. The treefixDTL software was used to “fix” non-significant conflicts between both phylogenies. Finally, the topological reconciliation of both trees with the program NOTUNG allowed a determination of the minimal number of authentic HOTs in the evolution of *Rhodobacteracae*.

### Phylogenetic analyses and tree reconciliation

For inferring organism trees, core-gene supermatrices of concatenated alignments of orthologous proteins were generated as previously described [28]. Maximum likelihood (ML) trees were inferred from the supermatrices with ExaML v3.0.19 [29] using maximum-parsimony starting trees, automated detection of the best substitution model, and 100 bootstrap replicates to estimate the statistical support of the internal nodes.

For collecting single genes, BlastP v2.7.1+ searches [30] were performed at the NCBI and Integrated Microbial Genome & Microbiomes (IMG) web sites (https://www.ncbi.nlm.nih.gov/; https://img.jgi.doe.gov/). Gene alignments were generated by Clustal Omega [31] and subsequently if necessary manually improved with the Edit option of the MUST package [32]. Highly variable positions and positions with >50% gaps were automatically removed by the program G-blocks [33] implemented in the software package MUST.

The ML-based phylogenetic inference was conducted with RAxML v8.2.4 [34] under a PROTGAMMALGF model; the rapid bootstrap option was used in conjunction with the autoMRE bootstopping criterion [35] and subsequent search for the best tree. Bayesian inference was done with the program PhyloBayes v3.3 under a CAT-GTR+4Γ model [36]. Two independent chains were run for 10,000 replicates and each tenth generation was sampled. The burn-in was estimated using the program “bpcomp” of the PhyloBayes package; the two chains were considered as converged if the maximal difference between the bipartitions was <0.25.

The significance of alternative topologies was estimated with approximately unbiased (AU) test [37].

The treefixDTL software [38] version 1.0.2 was applied to fix the topology of each associate tree (i.e., operon tree or gene tree) under default settings except for an alpha value of 0.001 for the paired-site test and the model closest to PROTGAMMALGF available via treefixDTL (PROTGAMMAJTTF) as RAxML substitution model. To reconstruct the types and numbers of the evolutionary events that explain the discrepancies, if any, between the final topologies, NOTUNG version 2.9 [39] was run under default settings except for the permission of horizontal transfers and the use of a DTL (duplication-transfer-loss) cost matrix of 2-3-1, corresponding to default costs used by treefixDTL.

The freeware program FigTree v1.3.1 for MacIntosh OSX computers created by Andrew Rambaut (Institute of Evolutionary Biology, University of Edinburgh; http://tree.bio.ed.ac.uk/) was used to draw the circular version of the schematic tree in Figure 5.

## Results

### Phylogenomic analysis of *Rhodobacteraceae*

**Fig. 1:**
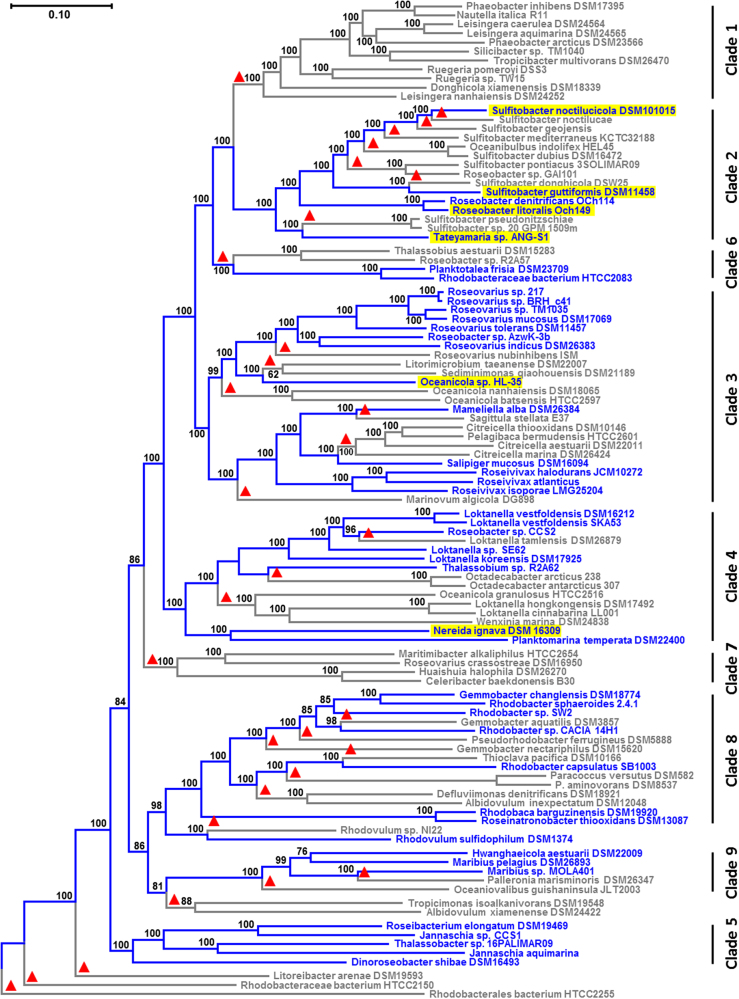
Phylogenomic tree of *Rhodobacteraceae*. Maximum-likelihood analysis of concatenated core-genome alignments with 194,215 amino acid positions from 105 sequenced genomes. The tree was inferred with ExaML under the optimal model and 100 bootstrap replicates conducted. Blue branches and labels display the extant distribution of photosynthetic strains, based on the assumption of (i) a photosynthetic ancestry of *Rhodobacteraceae* and (ii) a strict vertical evolution of the photosynthesis gene cluster (PGC). Red triangles mark independent PGC losses according to this “null-hypothesis”. Plasmid-located PGCs are highlighted in yellow. Rhodobacterales bacterium HTCC2255 was used as a close outgroup based on a preliminary analysis with more distantly related *Alphaproteobacteria* such as *Neomegalonema perideroedes* DSM 15528 (color figure online)

The selection of 105 genome-sequenced strains covering the phylogenetic diversity of the *Rhodobacteraceae* was focused not only on a representative set of 44 species containing the PGC but also included 61 non-photosynthetic relatives (Fig. 1). The number of species from strictly heterotrophic subtrees ranging, e.g., in clade 1 from *Phaeobacter inhibens* DSM 17395 to *Sedimentitalea nanhaiensis* DSM 24252 was reduced compared to the study of Simon et al. [28], and fast-evolving non-photosynthetic genera such as *Rubellimicrobium* and *Ketogulonicigenium* were omitted to minimize potential tree-reconstruction artifacts. The core genome of 580 orthologous proteins that are present in all 105 species was concatenated and the resulting alignment of 194,215 amino acid (aa) positions was used for the phylogenetic ML analysis (Fig. 1, Tab. S1, Dataset S1). The species tree was rooted with Rhodobacteraceae bacterium HTCC2255 in agreement with former phylogenomic analyses that showed the highly supported early-branching position of this strain in *Rhodobacteraceae* [28, 40]. We distinguished nine maximally supported clades (100% bootstrap proportion [BP]; Fig. 1). Branching pattern and denomination of clades 1–7 is congruent with two previous phylogenomic analyses that were focused on marine *Rhodobacteraceae* of the *Roseobacter* group [40, 41]. Clade 8 represents the Rhodobacter–Rhodobaca group, whose nested position among roseobacters has recently been shown [28], and clade 9 comprises recently sequenced genomes of the genera *Hwanghaeicola*, *Maribius*, *Palleronia*, and *Oceaniovalibus*. The relationships among the clades in the upper part of the figure (clades 1, 2, 6, 3, 4) and the position of the three early-branching strains, i.e., *Litoreibacter arenae* DSM 19593, HTCC2150, and HTCC2255, are supported by a 100% BP. In contrast, two *Rhodovulum* strains form the sister lineage of the Rhodobacter–Rhodobaca group with a 95% BP (clade 8; [42]). Furthermore, the relationships among the four remaining clades (7, 8 plus *Rhodovulum*, 9, 5) and a branch comprising the two species *Tropicimonas isoalkanivorans* DSM 19548 and *Albidovulum xiamenense* DSM 24422 are all supported by bootstrap values between 81 and 98%.

Photosynthetic representatives are widely distributed among the analyzed *Rhodobacteraceae* and found in seven of the nine clades (Fig. 1). All genome-sequenced representatives of clades 1 and 7 are heterotrophic, and the former comprises well-investigated model organisms such as *Ruegeria pomeroyi* DSS3, *Silicibacter* sp. TM1040, and *P. inhibens* DSM 17395 [43–45]. The 44 strains with a PGC are highlighted in all phylogenomic analyses of the current study by blue color. The scattered occurrence of phototrophic bacteria could—in agreement with the supposed photosynthetic origin of *Rhodobacteraceae* (null hypothesis)—be explained by 32 independent losses of the PGC, which are indicated by red triangles in Figure 1.

**Fig. 2:**
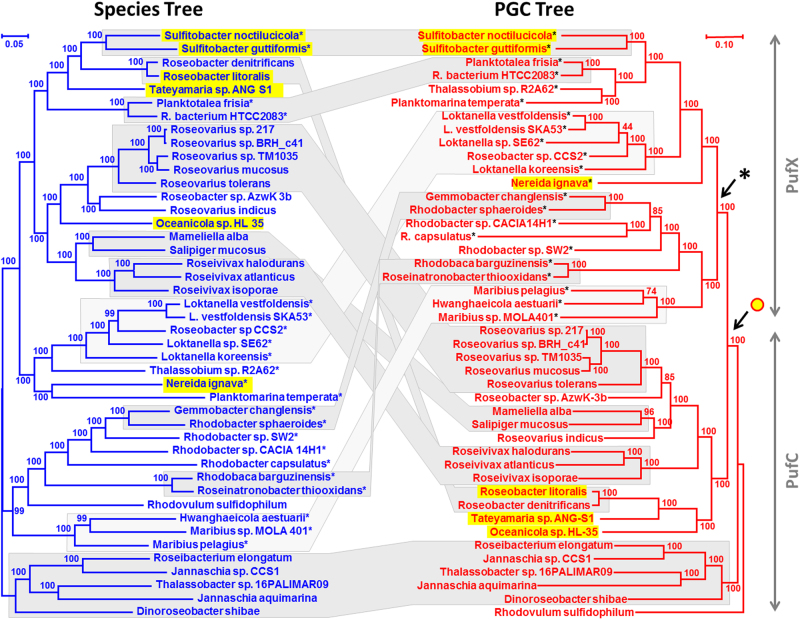
Comparison of species and photosynthesis gene cluster trees. Both the RAxML and the ExaML analyses were calculated based on an identical taxon sampling of 44 photosynthetic *Rhodobacteraceae*. **a** The phylogenomic species tree, which is shown in blue, comprises all 44 photosynthetic strains of the tree presented in Figure 1. It was inferred like the tree in Figure 1 based on 252,109 amino acid positions and rooted with five strains ranging from *Roseibacterium elongatum* to *Dinoroseobacter shibae* (clade 5, Fig. 1). For better comparability, the branching order of the photosynthetic strains in Figure 1 has been maintained in Figure 2. **b** The PGC tree, which is shown in red, is based on 33 concatenated PGC proteins with a largely common evolutionary history and a total of 11,225 amino acid positions after applying G-blocks. Plasmid-located PGCs are highlighted in yellow. The PGC tree is rooted with *Rhodovulum sulfidophilum* based on an additional analysis with four outgroup species (see Fig. S2). If possible, the strains were arranged in the same order as in the species tree. Gray blocks connect vertical evolving distal regions that are conserved between the two trees. Dark gray corresponds to an identical topology and light gray reflects similar topologies that can be reconciled with Treefix (significance level *p* = 0.05). Plasmid-located PGCs are highlighted in yellow. The arrow below the yellow circle indicates the putative last common ancestor with a DnaA-like I plasmid-located PGC. Stars indicate the presence of *pufX* in the PGC. The arrow below the star shows the replacement of the archetypal *pufC* gene by *pufX*. Note that the mean evolutionary rate of the PGC tree is roughly 1.5 times the one of the species tree (color figure online)

In order to trace the vertical evolution and potential horizontal transfers of PS in *Rhodobacteraceae*, a second species tree with the 44 strains containing the PGC was calculated (left tree, Fig. 2, Tab. S2, Dataset S2). The topology of both trees is virtually identical (Figs. 1 and 2), the sole exception is the altered position of *Hwanghaeicola aestuarii*, whose clustering with *Maribius pelagius* DSM 26893 is only weakly supported in Figure 1. The topology of the “PS” species tree is very well supported with 100% BP for all but three nodes that exhibit 99% BP. This was caused by the removal of non-photosynthetic strains including the three most divergent early-branching *Rhodobacteraceae* resulting in a larger number of characters (778 proteins; 252,109 aa positions). Thus the species tree can be solidly reconstructed with the 44 strains harboring the PGC.

### Phylogenetic analysis of the PGC

**Fig. 3:**
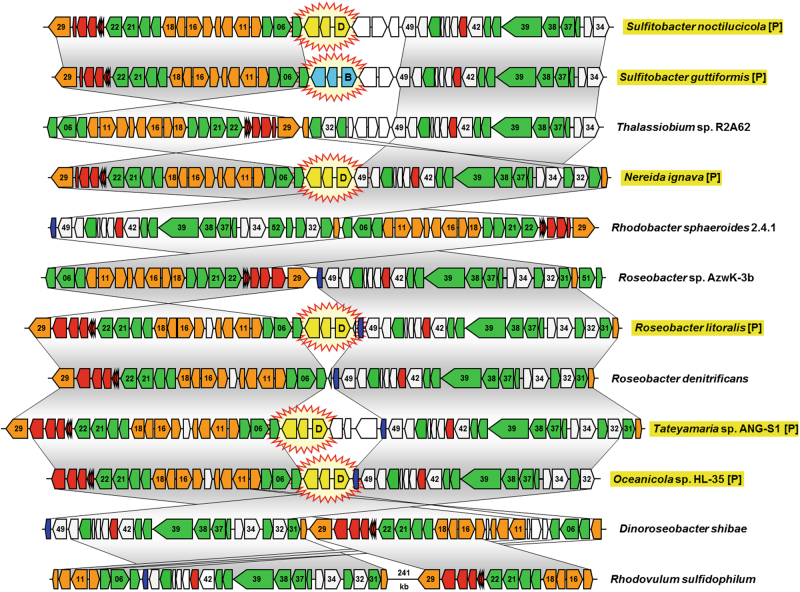
Comparison of six plasmid-located and six chromosomal photosynthesis gene clusters from *Rhodobacteraceae*. Selection of 12 among the 48 PGCs that were analyzed in the current study (see Tab. S4). Genes are colored according to biological categories: green, bacteriochlorophyll biosynthesis (*bch*); orange, carotenoid biosynthesis (*crt*); red, light-harvesting and photosynthesis reaction center (*puf*); dark blue, cytochrome *c*_*2*_ (*cycA*); gray, additional conserved genes of the PGC; white, non-conserved genes. The positioning of plasmid modules in the photosynthesis gene cluster, which are shown in yellow (DnaA-like I) and light blue (RepB-III), is highlighted by star-shaped icons. Identical gene order between PGCs is indicated by vertical gray shaded areas. Two conserved parts of the chromosomal PGC from *Rhodovulum sulfidophilum* are separated by a DNA stretch of 241-kb. Plasmid-located PGCs are highlighted in yellow and by the suffix [P] (color figure online)

The PGCs of *Alphaproteobacteria*, *Betaproteobacteria*, and *Gammaproteobacteria* contain a set of essential genes for PS [15, 16], which are orthologous and thus reflect a shared, concerted evolutionary history. The operon has not been independently assembled within different members of the group. This is supported by the high degree of structural conservation found for the 44 *Rhodobacteraceae* PGCs investigated in the current study; apart from sporadic recombination events, a conspicuous degree of conservation even between distantly related strains was observed (Fig. 3). In order to retrace the evolutionary history of the PGCs, we established a comprehensive set of 33 deduced proteins that were chosen based on strict exclusion criteria. The PGC from *Dinoroseobacter shibae* DFL-12, a photosynthetic model organism of the *Roseobacter* group [46], served as a reference (Table 1, Fig. S1). A total of 17 genes were excluded from further analyses, because they were either (i) not unique for the PGC or had (ii) <100 aa positions after applying G-blocks, which was used as the minimal amount of information for further processing (see below; Table 1). A concatenated protein alignment of the 33 remaining PGC markers with 10,971 aa was used in an initial ML (RAxML) analysis of 44 *Rhodobacteraceae* and four alphaproteobacterial outgroup sequences (Fig. S2A, Dataset S3), which allowed to determine the root of the ingroup. The early-branching position of *Rhodovulum sulfidophilum* DSM 1374 in the rhodobacteracean subtree is well supported (97% BP), and this strain was accordingly also used to root the *Rhodobacteraceae*-specific PGC tree that was calculated based on an alignment with 11,225 aa positions after G-blocks (Fig. S2B, Dataset S4). Both phylogenies have an identical topology apart from the non-resolved position of *Roseobacter* sp. AzwK-3b. The concatenation of 33 proteins resulted in a robust PGC tree with considerable statistical support for most branches (Fig. 2, right).

**Table 1:** List of PGC genes in *Rhodobacteraceae*

**No**.	**Gene**	**Function**	**Locus tag**	**Note**
01	*hemH*	Ferrochelatase	Dshi_3498	Not specific to PGC
02	hyp	Hypothetical methyltransferase	Dshi_3499	Not specific to PGC
03	*comF*	Competence protein F	Dshi_3500	Not specific to PGC
**04**	***crtA***	**Spheroidene monooxygenase**	Dshi_3501	
**05**	***bchI***	**Mg-chelatase, subunit I**	Dshi_3502	
**06**	***bchD***	**Mg-chelatase, subunit D**	Dshi_3503	
**07**	***bchO***	**Putative hydrolase**	Dshi_3504	
08	hyp	Hypothetical regulatory protein MarR family	Dshi_3505	Not specific to PGC
09	hyp	Hypothetical protein	Dshi_3506	Not specific to PGC
10	*grxC*	Glutaredoxin-3	Dshi_3507	Not specific to PGC
**11**	***crtI***	**Phytoene dehydrogenase**	Dshi_3508	
**12**	***crtB***	**Phytoene synthase**	Dshi_3509	
**13**	***tspO***	**Tryptophan-rich sensory protein** *(crtK)*	Dshi_3510	
14	hyp	Hypothetical protein	Dshi_3511	Not specific to PGC
**15**	***crtC***	**Hydroxyneurosporene dehydrogenase**	Dshi_3512	
**16**	***crtD***	**Methoxyneurosporene dehydrogenase**	Dshi_3513	
**17**	***crtE***	**Geranylgeranyl pyrophosphate synthase**	Dshi_3514	
**18**	***crtF***	**Hydroxyneurosporene-O-methyltransferase**	Dshi_3515	
**19**	***bchC***	**3-Hydroxyethyl bacteriochlorophyllide** ***a*** **dehydrogenase**	Dshi_3516	
**20**	***bchX***	**Bacteriochlorophyllide reductase, subunit X**	Dshi_3517	
**21**	***bchY***	**Bacteriochlorophyllide reductase, subunit Y**	Dshi_3518	
**22**	***bchZ***	**Bacteriochlorophyllide reductase, subunit Z**	Dshi_3519	
23	*pufQ*	Ferrochelatase regulator PufQ	Dshi_3520	<100 amino acids
24	*pufB*	Light-harvesting antenna LH1, beta subunit	Dshi_3521	<100 amino acids
25	*pufA*	Light-harvesting antenna LH1, alpha subunit	Dshi_3522	<100 amino acids
**26**	***pufL***	**Reaction center protein L**	Dshi_3523	
**27**	***pufM***	**Reaction center protein M**	Dshi_3524	
28	*pufC*	Reaction center cytochrome C	Dshi_3525 in some species alternated with PufX	
29	*DXPS*	1-Deoxy-D-xylulose-5-phosphate synthase	Dshi_3526	
30	*idi*	IPP isomerase	Dshi_3527	<100 amino acids
**31**	***bchP***	**Geranylgeranyl reductase**	Dshi_3528	
**32**	***pucC2***	**Putative LH assembly protein**	Dshi_3529	
**33**	***bchG***	**Bacteriochlorophyll synthase**	Dshi_3530	
**34**	***ppsR***	**Transcriptional regulator PpsR (CrtJ)**	Dshi_3531	
35	*ppaA*	Regulatory protein PpaA (AerR)	Dshi_3532	<100 amino acids
**36**	***bchF***	**3-Vinyl bacteriochlorophyllide** ***a*** **hydroxylase**	Dshi_3533	
**37**	***bchN***	**Protochlorophyllide reductase (DPOR), subunit N**	Dshi_3534	
**38**	***bchB***	**Protochlorophyllide reductase (DPOR), subunit B**	Dshi_3535	
**39**	***bchH***	**Mg-chelatase, subunit H**	Dshi_3536	
**40**	***bchL***	**Protochlorophyllide reductase (DPOR), subunit L**	Dshi_3537	
**41**	***bchM***	**Mg-protoporphyrin IX SAM O-methyltransferase**	Dshi_3538	
**42**	***lhaA***	**Putative assembly factor LhaA**	Dshi_3539	
**43**	***puhA***	**Reaction center protein H**	Dshi_3540	
**44**	***puhB***	**Putative assembly factor PuhB**	Dshi_3541	
45	*puhC*	PuhC protein	Dshi_3542	<100 amino acids
46	hyp	Hypothetical protein	Dshi_3543	<100 amino acids
**47**	***acsF***	**Mg-protoporphyrin IX oxidative cyclase, aerobic form**	Dshi_3544	
**48**	***puhE***	**Putative assembly factor PuhE**	Dshi_3545	
49	*hemA*	5-Aminolevulinate synthase	Dshi_3546	Not specific to PGC
50	*cycA*	Cytochrome c2	Dshi_3547	<100 amino acids
*Additional photosynthetic genes*				
51	*bchJ*	Hypothetical protein	Dshi_2636	Absent in some species
52	*bchE*	Mg-protoporphyrin IX oxidative cyclase, anaerobic form	Dshi_2637	Absent in some species

The genes are listed according to their order in *D. shibae* (see the locus tag numbers). Genes 51 and 52 are part of the PGC in *Rhodobacter*- and *Rhodobaca*-related strains. Number of amino acids is calculated after gap removal using G-blocks. Only the genes printed in bold were selected for the phylogenetic analyses

### Single-gene transfers

The PGC phylogeny with 44 strains served as a reference to systematically investigate the relevance of HGT for the composition of the PS machinery in *Rhodobacteraceae*. First, we determined the frequency of single-gene transfers by individually calculating RAxML phylogenies with bootstrap values of all 33 PS proteins and comparing the resulting trees with the PGC reference tree (Figs. S3-01 to S3-33; Fig. S2B). The detection of putative HGTs was based on the identification of those topological incongruencies that are supported by >70% BP in the single-gene phylogeny. This comparably stringent threshold allowed the identification of 73 conflicts (Tab. S3), which might either represent authentic HGTs or reflect tree reconstruction errors such as long branch attraction artifacts [47] or simply random error due to lack of information in short genes. The comparative analysis of the single-gene topologies and the PGC reference tree with TreeFix [38] allowed us to “fix” 33 conflicts (Fig. S3-21; Tab. S4). Six additional incongruencies, which concern, e.g., the position of *Loktanella* sp. SE62 in the BchB tree (gene #38) and the position of *Maribius* sp. MOLA401 in the AcsF tree (gene #47) (Figs. S3-25, S3-32), were weakly supported in the PGC phylogeny (70% BP; Fig. S2B) and were thus considered as “uncertainty of the reference tree.” The 34 remaining conflicts were regarded as authentic HGTs. A prime example is the *bchG* gene (#33) from *S. guttiformis* that groups together with *Loktanella koreensis* to the exclusion of *S. noctilucicola* that represents the sister strain in the PGC reference phylogeny (100% bootstrap contradiction; Fig. S3-21, Fig. S2B). Another example is the *bchH* gene (#39) from *D. shibae* that forms a separate branch with *Nereida ignava* (98% BP, Fig. S3-26), which is contradictory to the maximal supported positioning of *Dinoroseobacter* at the basis of a PGC subtree comprising the genera *Roseibacterium*, *Jannaschia*, and *Thalassobacter* (100% BP, Fig. S2B). Thus our investigations revealed a small number of single-gene replacements in the PGCs of *Rhodobacteraceae* (34/1452 genes; Tab. S3). This low rate of HGT-affected genes of 2.3% is in accordance with the high statistical support of the concatenated tree (Fig. S2).

### Concerted evolution of the PGC

The hypothesis of a mainly concerted evolution of the PGC genes entails several predictions, particularly given the evidence for HOTs. First, the number of horizontal transfers of each gene relative to the PGC tree should not necessarily be zero but lower than the number of its horizontal transfers relative to the organism tree; second, the number of horizontal transfers of each gene relative to the PGC tree should be lower than the number of HOTs. To test the hypothesis, we thus inferred trees for each PGC gene separately, “fixed” these gene trees by using either the PGC tree or the organism tree as reference tree, and conducted tree reconciliation with NOTUNG between each of the adapted gene trees and the reference tree that was used for “fixing” them. We also studied the stability of the results relative to the use of G-blocks alignment filtering and the TreeFix alpha value, which determines the number of topological changes when “fixing” gene trees.

The results are shown in Supplementary Text S1. For a TreeFix alpha value of 0.001, the median number of transfers relative to the PGC tree was only two, significantly lower than the number of HOTs inferred under the same settings. Moreover, the number of horizontal transfers of each gene relative to the PGC tree after adapting this gene tree to the PGC tree was significantly lower than the number of horizontal transfers of that gene tree relative to the organism tree after adapting that gene tree to the organism tree. This outcome was stable regarding the use of alignment filtering and the TreeFix alpha value, even though the alpha value, as expected, had a significant effect on the absolute number of inferred transfers.

The concerted evolution of the PGC therefore provides the basis for a comparison of the PGC tree and the species tree (see below). To improve our analysis, we decided to reduce the conflicting signal in our dataset and removed 34 unequivocal horizontally transferred genes from the concatenated alignment (Dataset S5). The topology of the final PGC tree that is presented in Figure 2b remains unchanged in comparison to Figure S2B, but some bootstrap values differ. For example, the statistical support for the already weakly supported positioning of *Loktanella* sp. SE62 was reduced (70 vs. 44% BP), whereas the backbone of the subtree ranging from *Roseovarius* sp. 217 to *Oceanicola* sp. HL-35 was better supported (49 vs. 85%, 92 vs. 100%, 75 vs. 100% BP; Fig. 2, Fig. S2).

### Horizontal transfer of the complete PGC

The comparison of the species-tree with the PGC tree in Figure 2 clearly shows that both phylogenies, which are based on the same strain sampling, are not congruent. Only some tips of the trees are conserved; nine subtrees with an identical topology are highlighted in dark gray and two additional subtrees with non-significant topological differences (see below) are shown in bright gray (Fig. 2, Fig. S4). The backbone of both trees is completely different and this can not be explained by tree reconstruction artifacts, because both phylogenies obtained solid statistical support. The capacity of anoxygenic PS was hence not strictly vertically inherited in the evolution of *Rhodobacteraceae*, thereby falsifying our null hypothesis of a common origin of the PGC and multiple losses (Fig. 1). The data unequivocally document HOTs of the complete PGC comprising operons and superoperons with genes for the RC, bacteriochlorophyll, and carotenoid biosynthesis (Fig. 3, Fig. S1). This does not exclude the possibility that independent PGC losses account in part for the current distribution of the PGC.

We then searched for the minimal number of HOTs that are required to reconcile the PGC tree with the species tree. First, we took the topologies—irrespective of some statistical weaknesses in the PGC tree—as they are and used them for a NOTUNG analysis [48]. This program reconciles an associate tree with a reference tree. Under the cost matrix for duplications, transfers, and losses as used by TreeFix, NOTUNG recovered a total of 64 equivalent and most parsimonious scenarios with 12 putative HOTs (transfers) and 12 corresponding losses of the PGC in the respective donor (Fig. S5). However, a few nodes of the PGC tree had a low statistical support, and this might decrease the number of significant conflicts with the species tree. Therefore, we tested a set of alternative topologies of the PGC tree with AU test [49] and could document that an alternate position of *Roseobacter* sp. CCS2 (44% BP), *Maribius* sp. MOLA401 (74% BP), and *Roseovarius* sp. AzwK-3b (85% BP), which corresponds to the topology of the species tree, is not significantly rejected (*p* = 0.05; Fig. S6A, B, C; Fig. 2). In contrast, the tested topological changes in the Rhodobacter–Rhodobaca group and of *R. sulfidophilum* were significantly rejected by AU test (Fig. S6E, F, I), which is indicative of authentic HOTs.

**Fig. 4:**
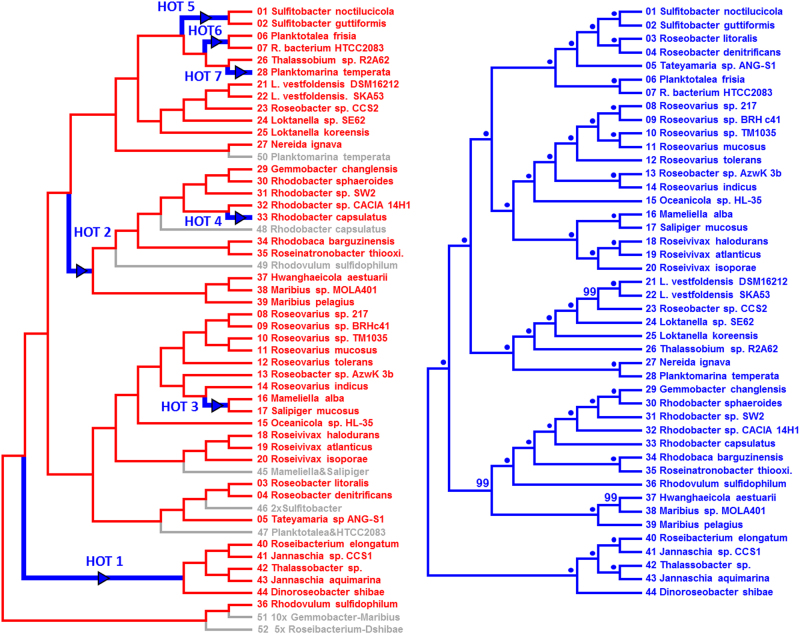
Reconciliation of the PGC and species tree. **a** NOTUNG analysis showing one of two optimal solutions for the reconciliation of the PGC and the species tree with seven HOTs and eight losses of the PGC (see also Fig. S7–1). The presented PGC tree contains five topological changes in comparison with Fig. 2b that were introduced by the program Treefix (*p* = 0.001; see also Fig. S8C). Strains are shown in red, because the “fixed” topology still corresponds to those of the PGC tree. HOTs of the PGC are highlighted by green arrows. PGC losses are indicated in gray. For a better understanding of the inferred losses, the names of the strains are provided (Fig. S7–1). **b** Cladogram of the green species tree shown in Figure 2a. Statistical support is provided; bootstrap proportion of 100% is indicated by a dot. The consecutive numbering of the tips in the species tree facilitates the comparison with the tips in the PGC tree. HOT horizontal operon transfer, PGC photosynthesis gene cluster (color figure online)

Second, a TreeFix analysis of the initial PGC tree and the corresponding species tree allowed us to “fix” 5 of the 12 conflicts (*p* = 0.001), which is compatible with the outcome of AU tests (Fig. S6). The topological differences between the PGC trees in Figs. 4a and 2b represent the five “fixed” conflicts (see also Fig. S8B, C). Accordingly, the subsequent NOTUNG analysis recovered a minimal number of seven authentic HOTs and eight losses (Fig. 4a; Fig. S7) that are required to explain the observed differences between the PGC and the species tree (Fig. 4). One example is HOT3 that corresponds to a horizontal transfer of the PGC in a common ancestor of *Mameliella alba* and *Salipiger mucosus*. The gray branch with the operational node “45” in the PGC tree (Fig. 4a) indicates the placement of the subtree as a sister lineage of the genus *Roseivivax* in the species tree (Fig. 4b). The sister group of the *Mameliella* and *Salipiger* subtree is supported by 100% BP in both phylogenies (Fig. 2, Fig. 4a). As a second example, HOT4 is solving the positional conflict of the well-investigated PGC of *Rhodobacter capsulatus* [50]. The scenario of the NOTUNG analysis suggests an early loss of the PGC in *R. capsulatus* and a subsequent replacement by the PGC of *Rhodobacter* sp. CACIA 14H1. Third, HOT5 explains the actual position of the plasmid-encoded PGCs from *Sulfitobacter noctilucicola* and *S. guttiformis* in the PGC tree, combined with the loss of the corresponding gray node “46” reflecting its position in the species tree. A more explicit scenario of the seven HOTs that illustrates the stepwise transition from the red PGC tree to the green species tree is shown in Figure S8. The blue proportion of the respective cladogram increases with each horizontal PGC transfer. The effect is highlighted by bold blue branches, which allows one to trace the above-mentioned HOTs 3, 4, and 5 (Fig. S8G, H, I). The probably most conspicuous difference between the PGC and the species tree in Figure 4 is the early-branching position of the well-investigated PGC of *R. sulfidophilum* [51, 52]. It was shown in a subanalysis with four non-rhodobacteracean PGCs, which were used to root the tree (Fig. S2A), and best explained by a single HOT from an outgroup taxon that was not sampled yet (see HOT1*, Fig. S8). Owing to the distinct rooting, the transfer scenarios of HOTs 1 and 2 in Figure 4 and Figure S8 are different. However, both scenarios that are presented in the current study independently showed that a minimum of seven HOTs were required to reconcile the “fixed” PGC tree with the species tree.

**Fig. 5:**
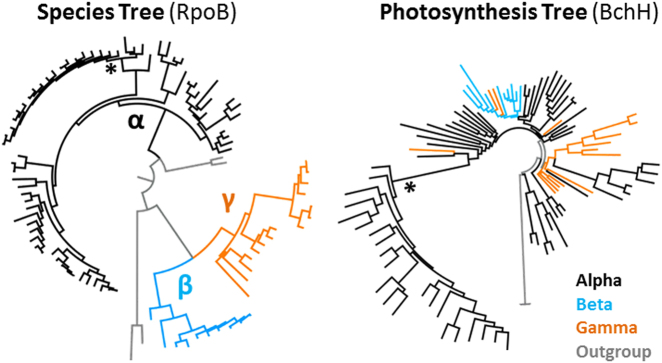
Relationships of *Alphaproteobacteria*, *Betaproteobacteria*, and *Gammaproteobacteria* in RpoB and BchH phylogenies. Schematic presentation of two PhyloBayes trees with 91 ingroup strains of photosynthetic *Proteobacteria* (see Figures S9, S10). The RpoB phylogeny reflects the organismal evolution (species tree) in contrast to the BchH phylogeny corresponding to the evolution of the PGC (photosynthesis tree). Alphaproteobacteria, betaproteobacteria, and gammaproteobacterial branches are shown in black, light blue, and orange, respectively. *Rhodobacteraceae (color figure online)

Frequent horizontal transfers of the PGC, which inter alia explain the early-branching position of *R. sulfidophilum* in the PGC tree (see HOT 1 in Fig. S8, Fig. 2, Fig. S2), challenge the former assumption of a photosynthetic ancestry of *Rhodobacteraceae*. The presence of three non-photosynthetic representatives at the basis of the species tree that is shown in Figure 1 might reflect their heterotrophic origin followed by a single recruitment of the PGC from an alphaproteobacterial donor (see supplemental Text S2; Fig. 5, Figs. S9, S10).

### Conservation of the PGC structure in *Rhodobacteraceae*

The structural conservation of the PGC in *Rhodobacteraceae* is shown in Figure 3. A comparison of the PGCs of all 44 investigated *Rhodobacteraceae* and four outgroup strains, which were used to root the PGC tree (Fig. S2A), is presented in Table S4. The order of tips from *S. noctilucicola* to *R. sulfidophilum* corresponds to the branching pattern of the phylogenetic tree (Fig. 2b). The numbering and color of the genes (based on the reference *D. shibae*; Fig. S1) provides an overview about the conspicuous conservation of the PGC organization in *Rhodobacteraceae*. Its reminiscence of a “theater seating plan” allows easily to retrace recombination events, but the extent of superoperon reshuffling is rather low (Fig. 3, Tab. S4). The structural composition of the whole PGC is in accordance with the phylogenetic tree and conserved over large evolutionary distances (Tab. S4, Fig. 2b). One example is the upper subtree ranging from *S. noctilucicola* to *N. ignava*. Another example is the PGC of *R. sulfidophilum*, which represents the outgroup of the phylogenetic tree (Fig. S2A) and exhibits a striking structural conservation with the equivalents of the early-branching subtree ranging from *Roseibacterium elongatum* to *D. shibae* (Tab. S4). This synteny is indicative of a common origin, thereby suggesting that it represents the ancestral PGC structure of *Rhodobacteraceae* prior to its subsequent spread via vertical evolution and horizontal transfer.

### Recruitment of *pufX* as a diagnostic event in PGC evolution of *Rhodobacteraceae*

Our comparative analysis of the 44 PGCs showed that the *pufC* gene (#28; Fig. S1), which encodes a RC-bound cytochrome subunit, is missing in the 22 strains that range from *S. noctilucicola* to *Maribius* sp. MOLA401 (Fig. 2b). The absence of *pufC* has previously been reported for *Rhodobacter sphaeroides* and several other proteobacterial lineages [53], thus indicating that its functional role as electron donor to bacteriochlorophyll can be taken over by alternative proteins. Interestingly, all 22 *pufC*-lacking *Rhodobacteraceae* harbor the *pufX* gene at precisely the same location downstream of the *pufQBALM* operon (#23 to #27) and upstream of the *dxps* gene for isoprenoid biosynthesis (#29; Tabs. S4, S5). PufX is a small essential protein involved in the assembly of the light-harvesting complex of *Rhodobacter* species [54], facilitating the dimerization of its RC [55]. In the PGC tree, all *pufX*-containing strains form a monophyletic group that is supported by a maximal BP (see arrow with star, Fig. 2). The nested position within the rooted PGC phylogeny clearly documents that the PufX type of the PS RC represents a secondary replacement of the archetypal PufC-type RC that happened late in the evolution of *Rhodobacteraceae*. The single ancestral acquisition of *pufX* represents a diagnostic event in the evolution of anoxygenic PS.

The presence of *pufX* genes has initially been reported for the genus *Rhodobacter* [56, 57], but they are also present in marine representatives of the *Roseobacter* group [58]. However, it was difficult to understand their evolutionary origin based on single-gene phylogenies of *pufL*, *pufM* and the 16S-rRNA. The establishment of the well-supported concatenated PGC tree in Figure 2 allowed us to pinpoint the origin of the PufX-type photosystem and to understand the scattered distribution of the two PGC types within the species tree (Fig. 1). The contemporary localization of *pufX* in five of the seven photosynthetic lineages of *Rhodobacteraceae* (clade 2, 6, 4, 9, and 8 [*Rhodobacter*–*Rhodobaca*]) and PufC in clade 2, 3, and 5 is the result of vertical evolution, PGC loss, and five of the seven HOTs reported in the current study (Fig. 4, Fig. S8).

### Localization of PGCs on plasmids

The presence of circular PS plasmids with DnaA-like I and RepB-III replication modules has previously been reported for *R. litoralis* and *S. guttiformis* [24, 25]. Here we found four novel plasmid-located PGCs, namely, in *S. noctilucicola*, *N. ignava*, *Tateyamaria* sp. ANG-S1, and *Oceanicola* sp. HL-35 and all harbor DnaA-like I replication systems (Fig. 3, Tab. S4). Remarkably, these replication modules are all located at precisely the same position within the PGC, providing strong evidence for their relevance for the horizontal transfers of the PGC (HOTs). One example is the distribution of extrachromosomal PGCs in the genus *Sulfitobacter*. The species tree shows a considerable phylogenetic distance between the two photosynthetic representatives *S. noctilucicola* and *S. guttiformis* with eight non-photosynthetic strains between them (Fig. 1), which makes a single plasmid-mediated HOT more probable than the alternative scenario of six independent PGC losses. The identical position of two different replication systems (DnaA-like I, RepB-III) within the highly conserved PGCs of both *Sulfitobacter* strains (Fig. 3, Tab. S4) is indicative of a secondary replacement of one module. Given the early-branching position of *N. ignava* in the PGC tree (Fig. 2b), which harbors a DnaA-like I replication system (Fig. 3), the most likely explanation is that the ancestral DnaA-like I system has been replaced by a RepB-III equivalent in *S. guttiformis*.

## Discussion

### Proof of horizontal PGC transfer in *Rhodobacteraceae*

A very recent review article about the origin and evolution of PS stated that “*horizontal gene transfer has played a substantial role in generating the highly dispersed distribution of photosynthesis that is observed among modern bacterial lineages*,” but it also concluded that “*there is no consensus concerning the number of transfers that took place during evolution or the directions in which those transfers occurred*” [5]. Former studies about the evolution of PS in *Proteobacteria* were based on single-gene phylogenies and a limited strain sampling [16, 18], and thus there was not enough statistical support in favor of a specific evolutionary scenario. The transfer of the complete PGC, which is based on the assumption that an adaptive advantage is only conferred to the new host if the module remains functional, has not been analyzed before. We were able to distinguish between (i) horizontal transfer of single genes of the PGC (HGTs) and (ii) HOTs.

HGTs occurred at a low rate and such rare events did not confound the concatenated PGC tree (Fig. 2b, Fig. S2). GTAs and phages represent the most likely vehicle for this mode of transfer. GTAs are present in the majority of *Rhodobacteraceae* and are able to package up to ~4 kb of chromosomal DNA [59]. Roseophages could also transfer small pieces of genetic information between species [60–62].

The current study provides the first clear proof that the capacity for PS has been horizontally transferred via HOTs, which was exemplified for the family *Rhodobacteraceae*. The prerequisite for our analyses was the clustering of all essential genes in the PGC. The phylogenomic species tree of 105 *Rhodobacteraceae* showed a scattered distribution of the 44 strains harboring the PGC (Fig. 1). According to our “null hypothesis”, which proposes a common photosynthetic origin of *Rhodobacteraceae* and a strictly vertical evolution of the PGC, at least 32 independent losses would have to be postulated to account for the phylogenetic distribution of the 61 non-photosynthetic strains. This “exclusive loss” scenario was tested and finally falsified by constructing a phylogenetic tree based on 33 unique genes of the PGC. The PS tree was extremely robust (100% bootstrap for most branches; Fig. 2b), which is in agreement with a modular evolution of the PGC, but it also showed multiple conflicts with the species tree (Fig. 2). Several methods were applied to determine the number of HOTs. Under the most stringent criteria, we detected a minimum number of at least seven authentic HOTs that are required to reconcile the PGC with the species tree (Fig. 4).

### Plasmids as evolutionary drivers of PS in *Rhodobacteraceae*

We suggest plasmids as vehicles for HOT, which is supported by the localization of the PGC on extrachromosomal elements in six phylogenetically distant species in *Rhodobacteraceae* (Fig. 1). Five of the PS plasmids harbor a DnaA-like I replication module (Fig. 3), which provides insights into the origin and timing of plasmid-borne HOTs. In *Rhodobacteraceae*, the PGC was at least once “outsourced” from the chromosome to a DnaA-like I type plasmid (Fig. 2). The deeply nested position of the chromosome-located PGC from *Roseobacter denitrificans* within a subtree of plasmid-located PGCs (Fig. 2b) provides evidence for a later reintegration into the chromosome (supplemental Text S3), thus completing the scenario for HOT [27]. DnaA-like plasmids are exclusively found in *Rhodobacteraceae* and the narrow host range is regarded as a diagnostic trait of this family [26, 28]. The localization of DnaA-like I plasmid replication modules at an identical, central position within the PGC is remarkable (Fig. 3), since it is found in distantly related species in the PGC phylogeny, spanning the subtree from *S. noctilucicola* to *Oceanicola* sp. HL-35 (Fig. 2b). This distribution documents that the reported PGC shuffling from the chromosome to a plasmid and back occurred in *Rhodobacteraceae* probably after the emergence of the photosynthetic lineage comprising five strains including *D. shibae* (Fig. 2). Furthermore, the whole operon structure of the plasmid-module-containing PGCs is nearly identical despite the distinct phylogenetic localization of the two *Sulfitobacter* strains, *Nereida*, and the subtree with *Roseobacter*, *Tateyamaria*, and *Oceanicola* (Tab. S4). Based on the currently analyzed strains, we hypothesize that the PGCs in *Rhodobacteraceae* have been horizontally transferred via DnaA-like I type plasmids after a single “chromosomal outsourcing” event in a common ancestor of the 39 PGCs ranging from *S. noctilucicola* to *Oceanicola* sp. HL-35 (see black arrow, Fig. 2b). For the remaining 33 PGCs, there were probably several reintegration events into the chromosome (“insourcing”) and they may have secondarily lost their plasmid replication systems due to the lack of function.

### Plasmid transfer in *Alphaproteobacteria*

Type-IV secretion systems (T4SS) for conjugation are abundant in *Rhodobacteraceae* [63] and have been shown to mediate conjugation between distantly related taxa [64]. So far they have not been detected on the PGC-containing plasmids. Given the presence of up to a dozen replicons in some representatives of the *Roseobacter* group [65], a recombination of PS and mobilizable plasmids should be no principle obstacle. Constructing vectors for the transfer of the complete PGC into a heterotrophic bacterium would be of huge interest not only from an evolutionary point of view but also for application in biotechnology and synthetic biology, and such a strategy is, for example, pursued for nitrogen-fixation modules in *Sinorhizobium meliloti* [66]. Examples for the shuffling of large functional modules between plasmid and chromosome and their horizontal transfer between different strains are the symbiosis islands in *Mesorhizobium* and *Bradyrhizobium* [67, 68]. It was even possible to introduce the symbiosis plasmid of *Cupriavidus taiwanensis*, a mimosa symbiont, into the pathogenic soil bacterium *Ralstonia solanaceum*, and by repeated sub-culturing in plant–bacteria co-cultures finally, mimosa symbionts of *R. solanaceum* were obtained [69]. An analysis of co-existing rhizobia in an agricultural soil showed that the ability to nodulate the bean *Phaseolus vulgaris* was strictly dependent on a symbiosis plasmid that was found in phylogenetically distinct taxa [70].

### Replacement of *pufC* by *pufX* in *Rhodobacteraceae*

*PufC* is a common part of the *puf* operon and widespread in phototrophic *Alphaproteobacteria*, *Betaproteobacteria*, and *Gammaproteobacteria* [58]. Interestingly, in many members of *Rhodobacteraceae* this gene is missing and its position in the operon is replaced by the *pufX* gene [57, 58]. The small PufX protein is an essential component of their RC light-harvesting complex and plays a key role in its dimerization and assembly [54, 55]. The distribution in the 44 photosynthetic *Rhodobacteraceae* analyzed here clearly shows that the gene replacement of *pufC* by *pufX* occurred only once in the evolution of their PGCs and divides extant phototrophs into two groups. The ancestral half of the phototrophic *Rhodobacteraceae* contains *pufC*, the derived half contains *pufX* (Fig. 2). Interestingly, the genus *Rhodobacter* harbors the derived *pufX* type of the PGC, which is in agreement with its previously shown nested (=non-early-branching) position within *Rhodobacteraceae* ([28]; Fig. 1). The observed distribution might either suggest that PufC and PufX are functionally equivalent for the structural integrity of the PS RC light-harvesting complex or that PufX confers an adaptive advantage. It would be interesting to further investigate these alternative explanations.

### Was the last common ancestor of *Rhodobacteraceae* photosynthetic?

The early-branching strains in the recent whole-genome phylogenies of the *Rhodobacteraceae* are not photosynthetic (Fig. 1; [28]), suggesting that their last common ancestor was heterotrophic and the PGC was introduced by a HOT. However, the discovery of a single photosynthetic strain with an ancestral position both within the species and the PGC tree would challenge this conclusion. Promising reference species for the future investigation of the photosynthetic ancestry of *Rhodobacteraceae* are the early-branching heterotrophic genera (*Amaricoccus*, *Albimonas*, *Oceanicella*), but especially the phototrophic representative *Rubrimonas cliftonenesis* DSM 15345 (Figs. S9, S10). The proof of horizontally transferred PGCs rebuts recent PufLM phylogeny-based conclusions about the lack of lateral PGC transfer at least for *Rhodobacteraceae* [71]. Our study showed (i) the enhanced resolution by concatenation of the PGC and it documented (ii) the need for a robust phylogenomic reference topology to avoid premature deductions.

New insights into the evolution of PS were provided in the past decade based either on the isolation of new species with an evolutionary key position or on metagenome analyses of the uncultured biodiversity. Two striking examples of “connecting links” are represented by the cyanobacterium *Gloeomargarita lithophora*, the closest cultured relative of primary plastids [72, 73], and the apicomplexan alga *Chromera velia*, a close photosynthetic relative of malaria parasites [74, 75]. The metagenomic discovery of two non-photosynthetic sister classes of *Cyanobacteria*, i.e. the *Melainabacteria* and *Sericytochromatia*, provided some evidence for a heterotrophic origin of this superensemble and a comparably late acquisition of PS by the last common cyanobacterial ancestor [4, 11]. The alternative explanation is a secondary loss of PS in the two non-photosynthetic sister lineages. Regarding the photosynthetic ancestry of *Cyanobacteria*, a genuine invention of PS seems to be plausible based on a parsimony-based “loss and gain” argumentation, whereas the orders of magnitude more frequent gain of PS in *Proteobacteria* likely reflects an evolutionary plug and play due to HOTs.

### HOTs and the distribution of anoxygenic PS in *Proteobacteria*

*Proteobacteria* and *Gemmatimonadetes* are the only phyla where PS genes are organized in typical PGCs [19, 76]. A similar clustering of the PS genes has also been reported for the firmicute *Heliobacterium modesticaldum* [77]. Our study provides for the first time clear evidence that the PGC was horizontally transferred at least seven times in the evolution of *Rhodobacteraceae*. Could similar mechanisms possibly account for the scattered distribution of the PGC in *Proteobacteria* in general? Structurally conserved PGCs with type-2 RCs that are homologous to rhodobacteracean PGCs are widely distributed among *Alphaproteobacteria*, *Betaproteobacteria*, and *Gammaproteobacteria* [15, 16]. Their phylogenetic distribution is scattered (Fig. 5; [8]). The PGC has been transferred at least once even into a different phylum, the *Gemmatimonadetes* [19, 78]. Natural plasmids with a broad host range such as pBBR1 from *Bordetella bronchiseptica* were manufactured to shuttle vectors [79, 80] that are functional in *Alphaproteobacteria*, *Betaproteobacteria*, and *Gammaproteobacteria* [81–83]. Therefore, we propose that the distribution of PS among *Proteobacteria* is mediated by HGT. PS plasmids have not yet been discovered in *Betaproteobacteria* and *Gammaproteobacteria*. In addition to conjugation, other mechanisms of HGT might be operating, e.g., transformation, which is already part of the GTA machinery [84]. Small extracellular vesicles have been discovered to be abundant in the ocean and to be produced by many marine bacteria; some of them contain DNA, and might act as vectors for HGT in the extremely dilute marine environment [85, 86]. However, in the light of hundreds of million years of bacterial evolution the extent of horizontal PGC transfer is rather low. All 44 analyzed PGCs have a common origin, and irrespective of the open question of the origin of PS in *Rhodobacteraceae*, there is so far no indication that this bacterial family has recruited the PGC a second time from other *Alphaproteobacteria*, *Betaproteobacteria*, or *Gammaproteobacteria*. The highly discrete phylogenetic position in the species and the PGC tree might furthermore reflect a lineage-specific evolution of PS in *Rhodobacteraceae*.

## Conclusion

In the current study, we provide a clear-cut evidence for the horizontal transfer of the complete PGC in *Rhodobacteraceae*. The scattered distribution of photosynthetic strains can be explained by vertical evolution, frequent PGC losses, and sporadic HOTs. The discovery of six extrachromosomal PGCs provided the basis for an evolutionary scenario proposing a plasmid-based transfer of PS at least in this family of *Alphaproteobacteria*.

## Electronic supplementary material

Dataset S1

Dataset S2

Dataset S3

Dataset S4

Dataset S5

Table S1

Table S2

Table S3

Table S4

Table S5

Text S1

Text S2

Text S3

Figure S1

Figure S2

Figure S3

Figure S4

Figure S5

Figure S6

Figure S7

Figure S8

Figure S9

Figure S10
