# Supplementary material for: Horizontal operon transfer, plasmids, and the evolution of photosynthesis in Rhodobacteraceae
Source: ISME J. 2018 May 24;12(8):1994–2010. doi: 10.1038/s41396-018-0150-9 (PMC6052148; doi:10.1038/s41396-018-0150-9)
Supplement: Supplementary file 11 — Text S1 [file 41396_2018_150_MOESM11_ESM.pdf]

Supplementary Text S1.

Analyses of single-gene trees with TreeFix and Notung

Trees inferred from the either the G-blocks filtered or original alignments of the selected genes belonging to the PGC were ‘fixed’ with TreeFix using either the PGC tree or the organism tree as reference tree and three distinct alpha values (0.001, 0.01 and 0.05), respectively. This yielded a total of 2 × 2 × 3 = 12 settings per gene. Tree reconciliation was done for each gene and each setting with Notung using the same costs matrix as for TreeFix and the corresponding reference tree.

The dependency of the costs was and determined in R version 3.4.3 using a linear model considering variable interactions, followed by step-wise, bidirectional variable elimination:

```
m1 <- with(x, lm(Score ~ Referencetree * Gblocks * Alpha))
m1 <- step(object = m1, direction = "both")
summary(m1)
```

The resulting model was as follows (the display within R is pictured):

```
Call:
lm(formula = Score ~ Referencetree + Alpha)

Residuals:
    Min      1Q  Median      3Q     Max
-22.205  -7.018  -0.311   5.596  38.689

Coefficients:
                 Estimate Std. Error t value Pr(>|t|)
(Intercept)        25.363      1.216  20.859  < 2e-16 ***
ReferencetreePGC  -15.053      1.203 -12.514  < 2e-16 ***
Alpha0.01           6.093      1.444   4.220 3.28e-05 ***
Alpha0.05          11.895      1.460   8.148 1.17e-14 ***
---
Signif. codes:  0 ‘***’ 0.001 ‘**’ 0.01 ‘*’ 0.05 ‘.’ 0.1 ‘ ’ 1

Residual standard error: 10.15 on 286 degrees of freedom
  (106 observations deleted due to missingness)
Multiple R-squared:  0.4389,    Adjusted R-squared:  0.433
F-statistic: 74.58 on 3 and 286 DF,  p-value: < 2.2e-16
```

Then use of G-blocks for alignment filtering is irrelevant (the variable gets eliminated). The reference tree has the most significant effect on the score. Using the PGC tree as reference tree (instead of the species tree) decreases the score, i.e. improves the fit between the gene tree and the reference tree and causes fewer evolutionary events to be postulated in addition to vertical transfers. Using higher alpha values (0.01 and particularly 0.05) causes more paired-site tests to be judged significant by TreeFix and thus fewer topological adaptations ('fixes') to be made by TreeFix, hence even the resulting 'fixed' topologies more strongly differ under these settings from the reference tree and the score gets increased.

Figures N1 and N2 show the scores of each single-gene tree in dependency on alpha and the reference tree, when omitting or using G-blocks, respectively. Figures N3 and N4 show the difference between the score obtained with the organism tree as reference tree and the score obtained with the PGC tree as reference tree of each single-gene tree in dependency on alpha, when omitting or using G-blocks, respectively. Obviously, the mean difference is significantly larger than zero, hence the score obtained with the species tree as reference tree is significantly larger, under all settings investigated.

The optical impression was confirmed with t-tests, which were significant ($p < 0.001$) under all six settings (two kinds of alignments times three values of alpha).

Figures N5-N8 show the same as N1-N4 but with the inferred number of horizontal transfers as dependent variable instead of the score. The median number of transfers when the PGC tree is used as reference tree is significantly lower then otherwise. Note that the number of transfers is also lower than the one inferred in the comparison between the PGC tree and the species tree (see main manuscript).

It should be noted that Notung discards solutions that are optimal according to the cost matrix but not 'temporarily feasible' (because they imply conflicting time points of transfers between ancestors). When all initial solutions are filtered out, this yields NA ('not available') values of the Notung score in R, which automatically get removed from all subsequent calculations.

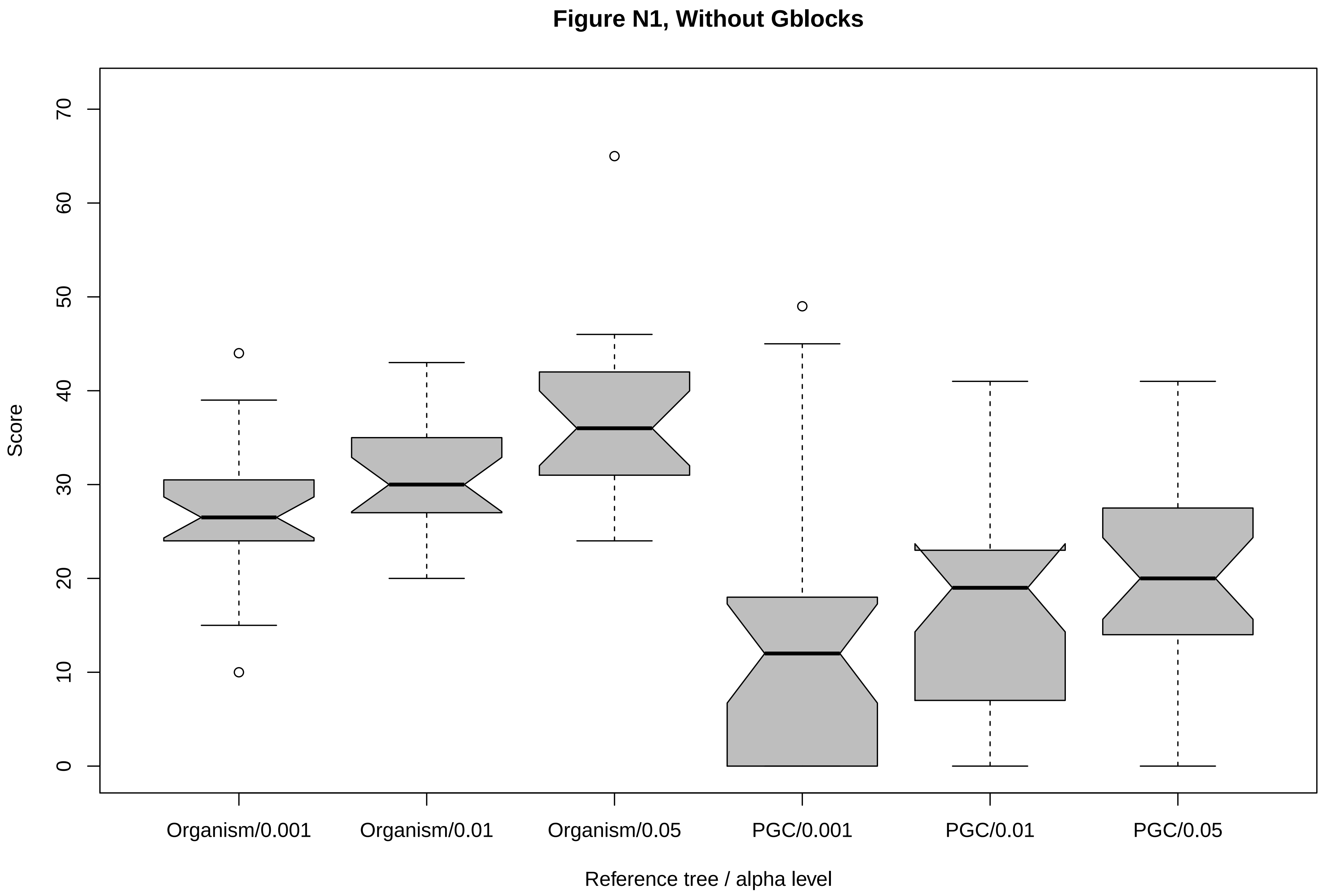
Figure N1, Without Gblocks
Score
0
10
20
30
40
50
60
70
Organism/0.001
Organism/0.01
Organism/0.05
PGC/0.001
PGC/0.01
PGC/0.05
Reference tree / alpha level

Figure N2, With Gblocks

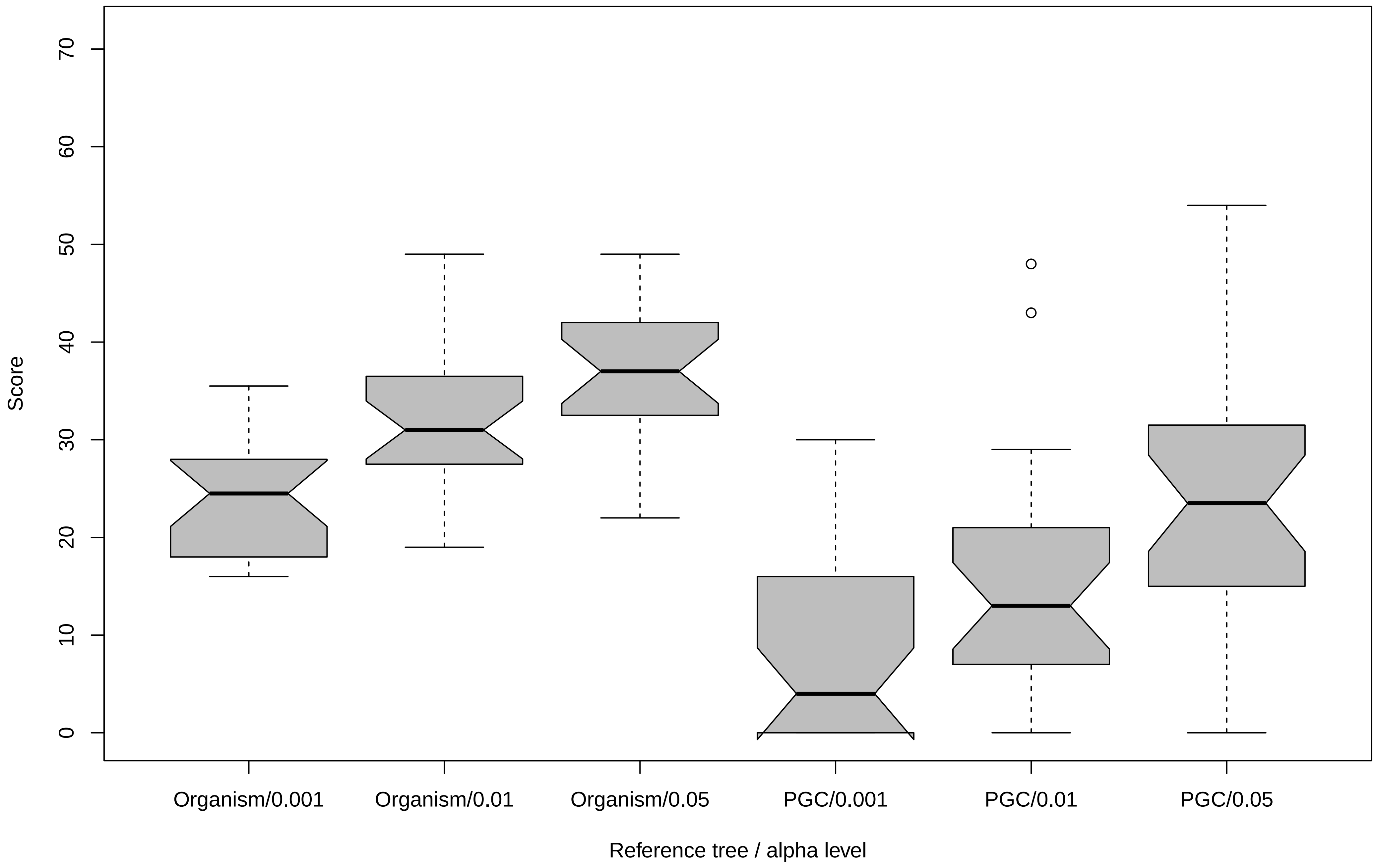

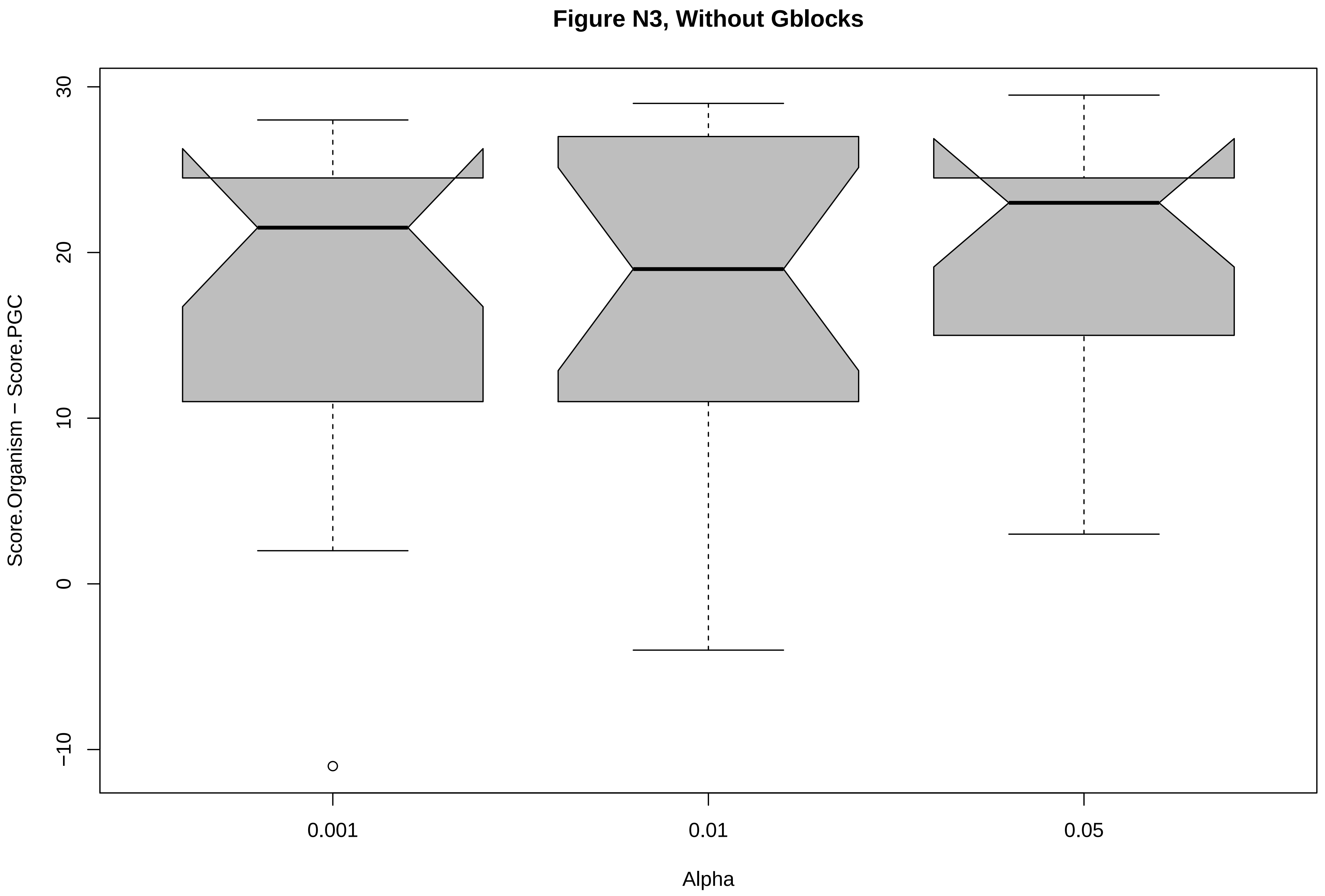
Figure N3, Without Gblocks
Score.Organism − Score.PGC
30
20
10
0
−10
0.001
0.01
0.05
Alpha

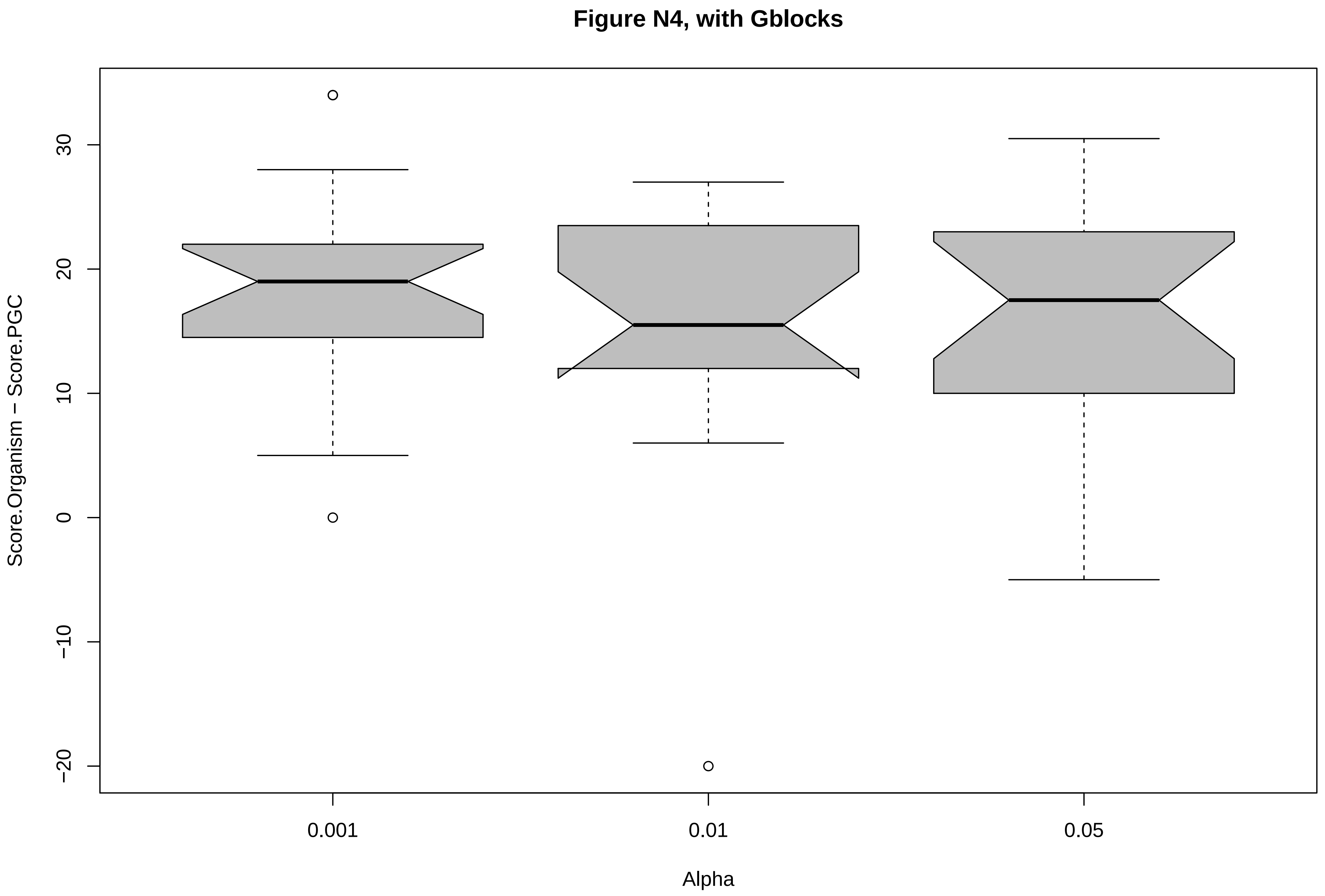
Figure N4, with Gblocks
Score.Organism − Score.PGC
30
20
10
0
−10
−20
0.001
0.01
0.05
Alpha

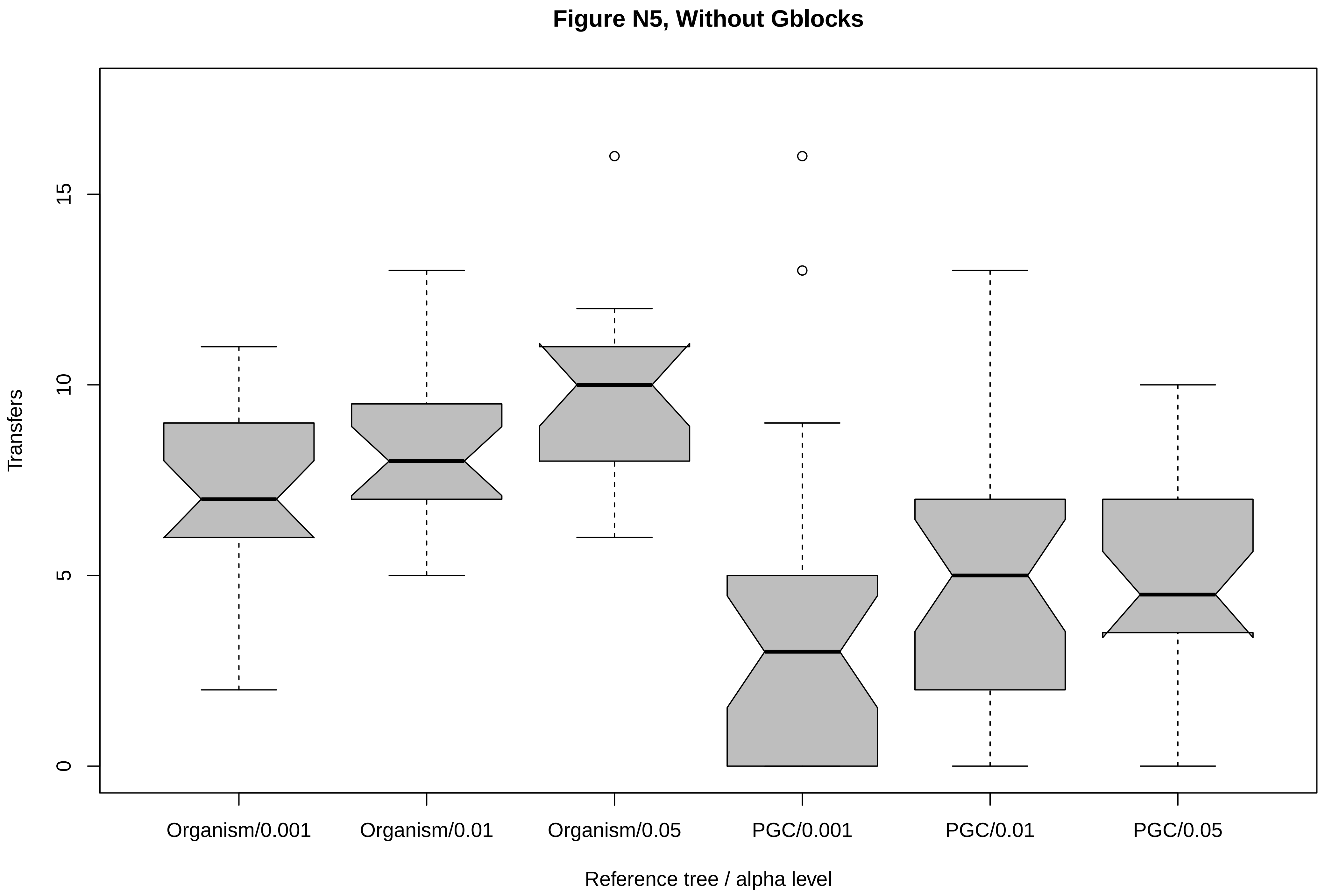

Figure N5, Without Gblocks
Transfers
0
5
10
15
Organism/0.001
Organism/0.01
Organism/0.05
PGC/0.001
PGC/0.01
PGC/0.05
Reference tree / alpha level

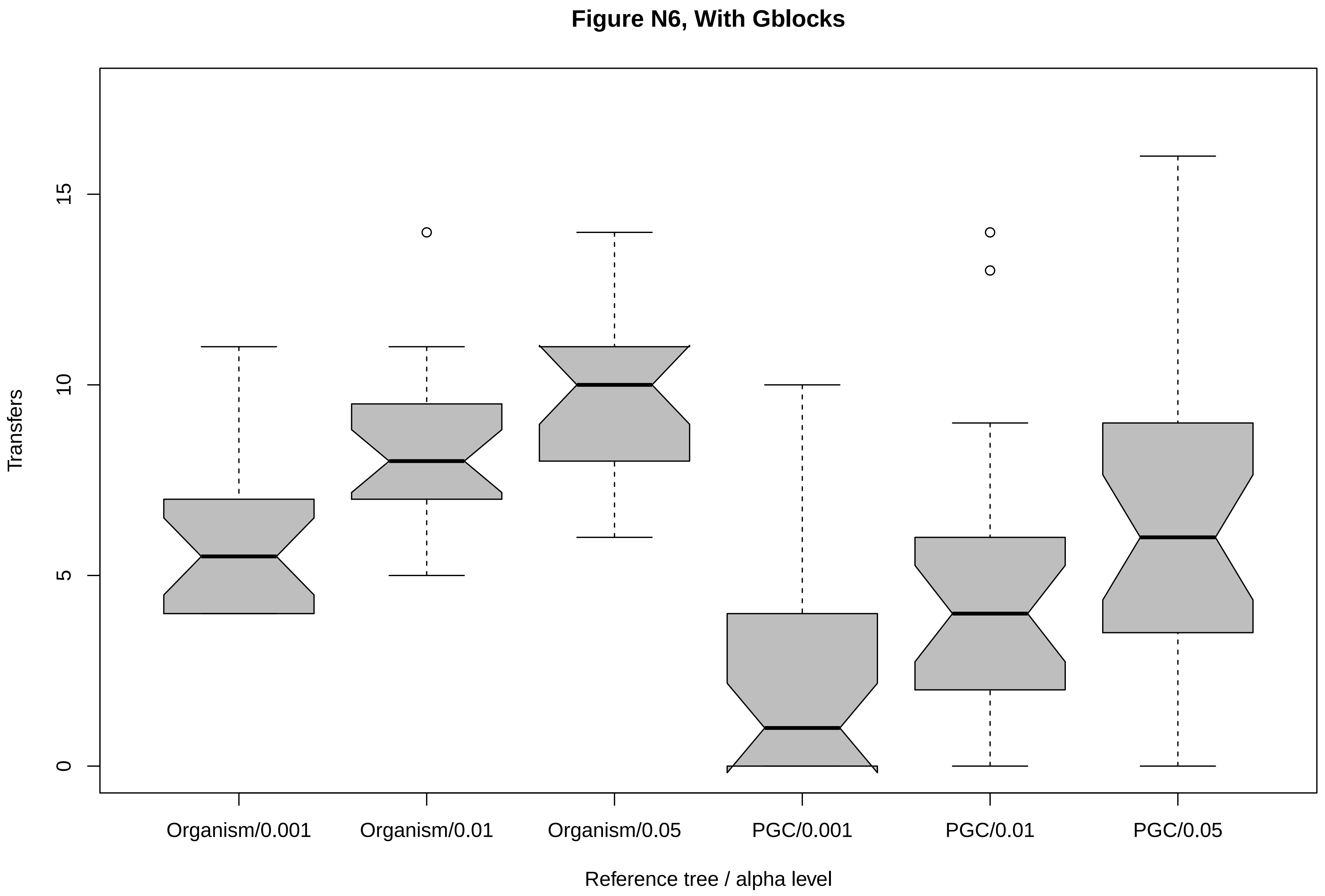
Figure N6, With Gblocks
Transfers
0
5
10
15
Organism/0.001
Organism/0.01
Organism/0.05
PGC/0.001
PGC/0.01
PGC/0.05
Reference tree / alpha level

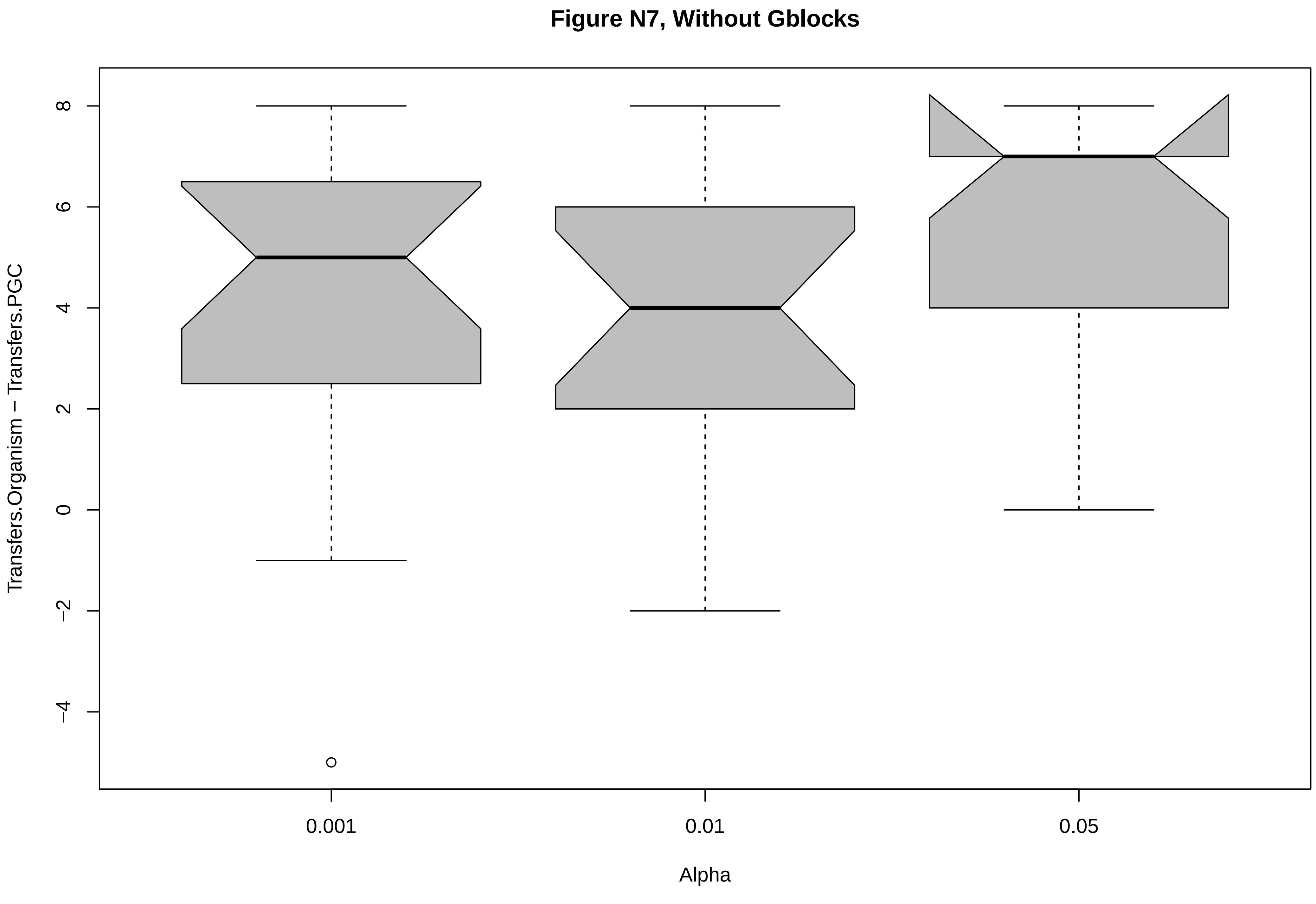

Figure N7, Without Gblocks
Transfers.Organism − Transfers.PGC
8
6
4
2
0
−2
−4
0.001
0.01
0.05
Alpha

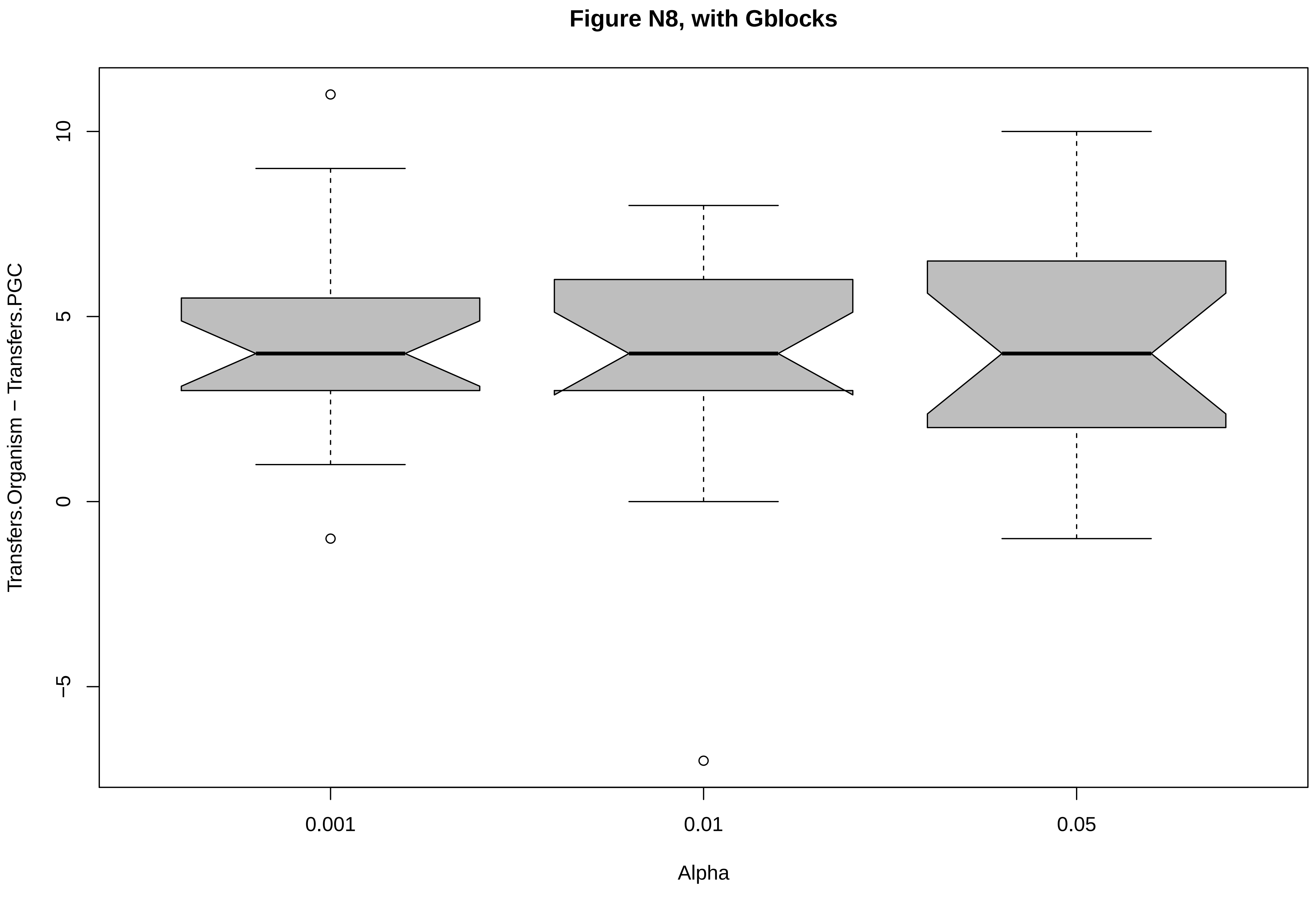
Figure N8, with Gblocks
Transfers.Organism − Transfers.PGC
10
5
0
−5
0.001
0.01
0.05
Alpha
