# Supplementary material for: Horizontal operon transfer, plasmids, and the evolution of photosynthesis in Rhodobacteraceae
Source: ISME J. 2018 May 24;12(8):1994–2010. doi: 10.1038/s41396-018-0150-9 (PMC6052148; doi:10.1038/s41396-018-0150-9)
Supplement: Supplementary file 12 — Text S2 [file 41396_2018_150_MOESM12_ESM.pdf]

Supplementary Text S2.

Phylogenetic RpoB and BchH analyses

Sequences of the beta subunit of the RNA polymerase (RpoB) from 91 photosynthetic *Alpha*-, *Beta*- and *Gammaproteobacteria* were analysed to retrace their phylogenetic relationships (Fig. 5A, Fig. S9). Eight additional sequences of non-photosynthetic *Proteobacteria* were included into the analysis that either harbor a phylogenetic key position (*Alpha*-, *Zetaproteobacteria*) or were used to root the tree (*Deltaproteobacteria*). The RpoB phylogeny is based on a dataset with 1227 aa positions and the expected branching of the photosynthetic representatives of the three proteobacterial classes was inferred. *Alpha*-, *Beta*-, and *Gammaproteobacteria*, which are located in distinct well-supported subtrees (100%, 100%, 90% BP), are highlighted in black, orange and blue, respectively; the outgroup taxa are shown in gray (Fig. 5, Fig. S9). The RpoB tree shows the joint grouping of *Beta*- and *Gammaproteobacteria* forming together with the *Zetaproteobacteria* a sister group of *Alphaproteobacteria* to an exclusion of *Deltaproteobacteria*. The long basal branches of the different lineages are remarkable, because they reflect the enormous evolutionary distances between these five proteobacterial classes. The phylogeny also recovers the five photosynthetic orders among the *Alphaproteobacteria*, i.e. *Rhodospirillales* (89% BP), *Sphingomonadales* (100% BP), *Caulobacterales* (100% BP), *Rhizobiales* (87% BP) and a distinct subtree of *Rhodobacterales* (100% BP), which comprises *Rhodobacteraceae* that were investigated in the current study (Fig. 1). The RpoB phylogeny reflects according to its canonical branching pattern the 'species tree' of *Proteobacteria*.

A phylogeny with an identical ingroup sampling (91 taxa) and *Gemmatimonas phototrophica* issued as a close outgroup (Zeng et al., 2015) was calculated for bacteriochlorophyll H (Fig. 5B, Fig. S10). BchH represents the largest marker of the PGC (#39; Fig. S1) and the tree is based on a dataset with 1062 aa positions. The clustering of photosynthesis genes in a superoperon is

characteristic for *Alpha*-, *Beta*- and *Gammaproteobacteria* (Fuchs et al., 2007; Zheng et al., 2011), but it was also observed in *Gemmatimonadetes* (Zeng et al., 2016). The BchH phylogeny is hence considered to be a proxy for the 'photosynthesis tree'. It should allow drawing first clues about the evolution of the PGC in *Proteobacteria* irrespective of sporadic HGTs that have been observed in *Rhodobacteraceae* (see above; Fig. S3-26). The schematic overview in Figure 5 provides - as illustrated by the color code - a first impression of the scattered distribution of *Alpha*-, *Beta*- and *Gammaproteobacteria* in the photosynthesis tree, which is strikingly different to their clear separation in the species tree. The solid backbone of the RpoB tree with long basal branches does not exist in the corresponding BchH analysis, thus indicating that the last common ancestor of the contemporary PGCs in *Proteobacteria* originated comparably late and does not reflect the organismal separation of the three classes. Evidence for massive horizontal transfers of the photosynthesis superoperon (HOTs) is furthermore provided by the placement of *Rhodospirillales* and *Rhizobiales* that are both found in at least five different positions in the BchH tree (Fig. S10). Examples of putative HOTs are provided by *Oceanibaculum indicum* P24 (*Rhodospirillales*) that is located among Rhizobia and two distantly related *Gammaproteobacteria* (*Nevskia ramosa*, *Ectothiorhodospira magna*) that are deeply nested within the betaproteobacterial subtree (Fig. 5).

References

Fuchs, B. M., Spring, S., Teeling, H., Quast, C., Wulf, J., Schattenhofer, M., et al. (2007). Characterization of a marine gammaproteobacterium capable of aerobic anoxygenic photosynthesis. *Proc. Natl. Acad. Sci.* 104, 2891–2896. doi:10.1073/pnas.0608046104.

Zeng, Y., Baumbach, J., Vieira Barbosa, E. G., Azevedo, V., Zhang, C., and Koblížek, M. (2016). Metagenomic evidence for the presence of phototrophic Gemmatimonadetes bacteria in diverse environments. *Environ. Microbiol. Rep.* 8, 139–149. doi:10.1111/1758-2229.12363.

Zeng, Y., Selyanin, V., Lukes, M., Dean, J., Kaftan, D., Feng, F., et al. (2015). Characterization of the microaerophilic, bacteriochlorophyll a-containing bacterium Gemmatimonas phototrophica sp. nov., and emended descriptions of the genus Gemmatimonas and Gemmatimonas aurantiaca. *Int. J. Syst. Evol. Microbiol.* 65, 2410–2419. doi:10.1099/ijs.0.000272.

Zheng, Q., Zhang, R., Koblížek, M., Boldareva, E. N., Yurkov, V., Yan, S., et al. (2011). Diverse arrangement of photosynthetic gene clusters in aerobic anoxygenic phototrophic bacteria. *PLoS One* 6, e25050. doi:10.1371/journal.pone.0025050.
