# Supplementary material for: Horizontal operon transfer, plasmids, and the evolution of photosynthesis in Rhodobacteraceae
Source: ISME J. 2018 May 24;12(8):1994–2010. doi: 10.1038/s41396-018-0150-9 (PMC6052148; doi:10.1038/s41396-018-0150-9)
Supplement: Supplementary file 13 — Text S3 [file 41396_2018_150_MOESM13_ESM.pdf]

Supplementary Text S3.

Chromosomal insourcing of the PGC exemplified for *Roseobacter denitrificans*

Novel insights into the mechanism of chromosomal integration of horizontally transferred PGCs are provided by the PGC-subtree comprising *Roseobacter litoralis*, *Roseobacter denitrificans*, *Tateyamaria* sp. ANG-S1 and *Oceanicola* sp. HL-35 (Fig. 2B). The structural composition of their PGCs is almost identical, the sole exception is the lack of the DnaA-like I replication module in *R. denitrificans* (Fig. 3, Tab. S2). Based on the incongruent branching pattern of PGC and species trees (compare Fig. 2B with Fig. 2A), the capacity of anaerobic photosynthesis was most likely horizontally transferred via the prevalent DnaA-like I plasmid-type. Based on Occam´s razor (parsimony), the chromosomal localization of the PGC in *R. denitrificans* is best explained by 'chromosomal insourcing' i.e an intracellular transfer of the PGC from the plasmid to the chromosome followed by a secondary loss of the no longer required replication module. This scenario is diametrically opposed to the formerly proposed 'chromosomal outsourcing' as favored explanation for the different localization of the PGC on a plasmid in *R. litoralis* and on the chromosome in *R. denitrificans* (Petersen et al., 2012, 2013). It represents an example how parsimony-driven conclusions depend on the comprehensiveness (size, quality) of the datasets. However, irrespective of the ultimate scenario of donors and recipients, the discovery of four additional extrachromosomal PGCs provided strong evidence for the impact of plasmid-mediated HOTs on the evolution of photosynthesis in *Rhodobacteraceae*.

References

Petersen, J., Brinkmann, H., Bunk, B., Michael, V., Päuker, O., and Pradella, S. (2012). Think pink: photosynthesis, plasmids and the Roseobacter clade. *Environ. Microbiol.* 14, 2661–2672. doi:10.1111/j.1462-2920.2012.02806.x.

Petersen, J., Frank, O., Göker, M., and Pradella, S. (2013). Extrachromosomal, extraordinary and essential - the plasmids of the Roseobacter clade. *Appl. Microbiol. Biotechnol.* 97, 2805–2815. doi:10.1007/s00253-013-4746-8.
