# Supplementary material for: Horizontal operon transfer, plasmids, and the evolution of photosynthesis in Rhodobacteraceae
Source: ISME J. 2018 May 24;12(8):1994–2010. doi: 10.1038/s41396-018-0150-9 (PMC6052148; doi:10.1038/s41396-018-0150-9)
Supplement: Supplementary file 14 — Figure S1 [file 41396_2018_150_MOESM14_ESM.pdf]

Figure S1

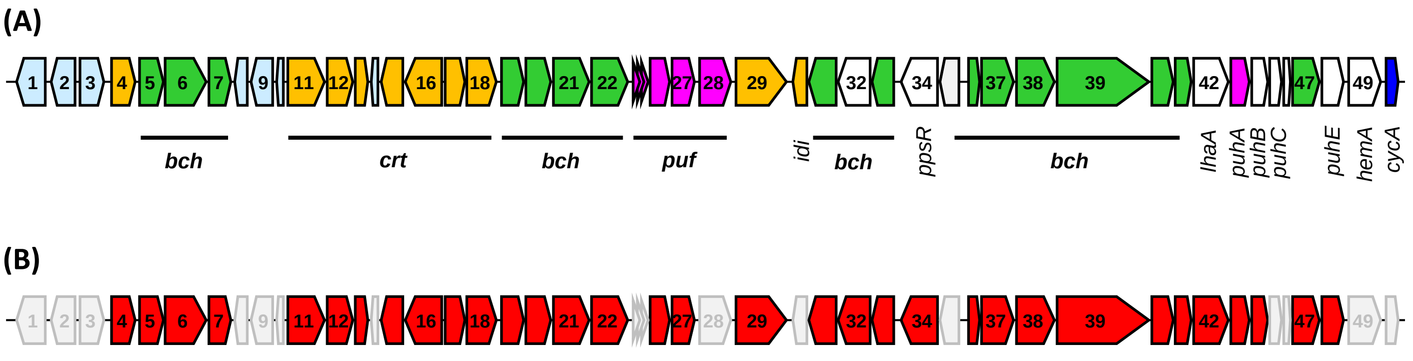

01 *hemH* (Dshi_3498)
02 hypo (Dshi_3499)
03 *comF* (Dshi_3500)
04 *crtA* (Dshi_3501)
05 *bchI* (Dshi_3502)
06 *bchD* (Dshi_3503)
07 *bchO* (Dshi_3504)
08 *marR* (Dshi_3505)
09 hypo (Dshi_3506)
10 *grxC* (Dshi_3507)
11 *crtI* (Dshi_3508)
12 *crtB* (Dshi_3509)
13 *tspO* (Dshi_3510)
14 hypo (Dshi_3511)
15 *crtC* (Dshi_3512)
16 *crtD* (Dshi_3513)
17 *crtE* (Dshi_3514)
18 *crtF* (Dshi_3515)
19 *bchC* (Dshi_3516)
20 *bchX* (Dshi_3517)
21 *bchY* (Dshi_3518)
22 *bchZ* (Dshi_3519)
23 *pufQ* (Dshi_3520)
24 *pufB* (Dshi_3521)
25 *pufA* (Dshi_3522)
26 *pufL* (Dshi_3523)
27 *pufM* (Dshi_3524)
28 *pufC** (Dshi_3525)
29 *dxps* (Dshi_3526)
30 *idi* (Dshi_3527)
31 *bchP* (Dshi_3528)
32 *pucC* (Dshi_3529)
33 *bchG* (Dshi_3530)
34 *ppsR* (Dshi_3531)
35 *ppsA* (Dshi_3532)
36 *bchF* (Dshi_3533)
37 *bchN* (Dshi_3534)
38 *bchB* (Dshi_3535)
39 *bchH* (Dshi_3536)
40 *bchL* (Dshi_3537)
41 *bchM* (Dshi_3538)
42 *lhaA* (Dshi_3539)
43 *puhA* (Dshi_3540)
44 *puhB* (Dshi_3541)
45 *puhC* (Dshi_3542)
46 hypo (Dshi_3543)
47 *acsF* (Dshi_3544)
48 *puhE* (Dshi_3545)
49 *hemA* (Dshi_3546)
50 *cycA* (Dshi_3547)

Further PS-genes
51 *bchJ* (Dshi_2636)
52 *bchE* (Dshi_2637)

Figure S1. Photosynthesis gene cluster from *Dinoroseobacter shibae* DFL-12. **(A)** Coloring of the PGC according to Petersen et al. (2012). green, bacteriochlorophyll biosynthesis (*bch*); orange, carotenoid biosynthesis (*crt*); pink, photosynthesis reaction centre (*puf*); dark blue, cytochrome c_2 (*cycA*). Genes shown in white are conserved among the PGCs (see also Table S2); light blue ones are specific for *D. shibae*. **(B)** 33 PGC genes that are used for the phylogenetic analyses of the current study are highlighted in red. The removal of the remaining 17 genes is explained in Table S1. PGC, photosynthesis gene cluster. **pufC* is only present in a subset of PGCs, in other species it is replaced by *pufX* (see Fig. 2).
