# Supplementary material for: Horizontal operon transfer, plasmids, and the evolution of photosynthesis in Rhodobacteraceae
Source: ISME J. 2018 May 24;12(8):1994–2010. doi: 10.1038/s41396-018-0150-9 (PMC6052148; doi:10.1038/s41396-018-0150-9)
Supplement: Supplementary file 15 — Figure S2 [file 41396_2018_150_MOESM15_ESM.pdf]

Figure S2

Phylogenetic RAxML Analyses (LGF4Γ; 100 bootstrap replicates)

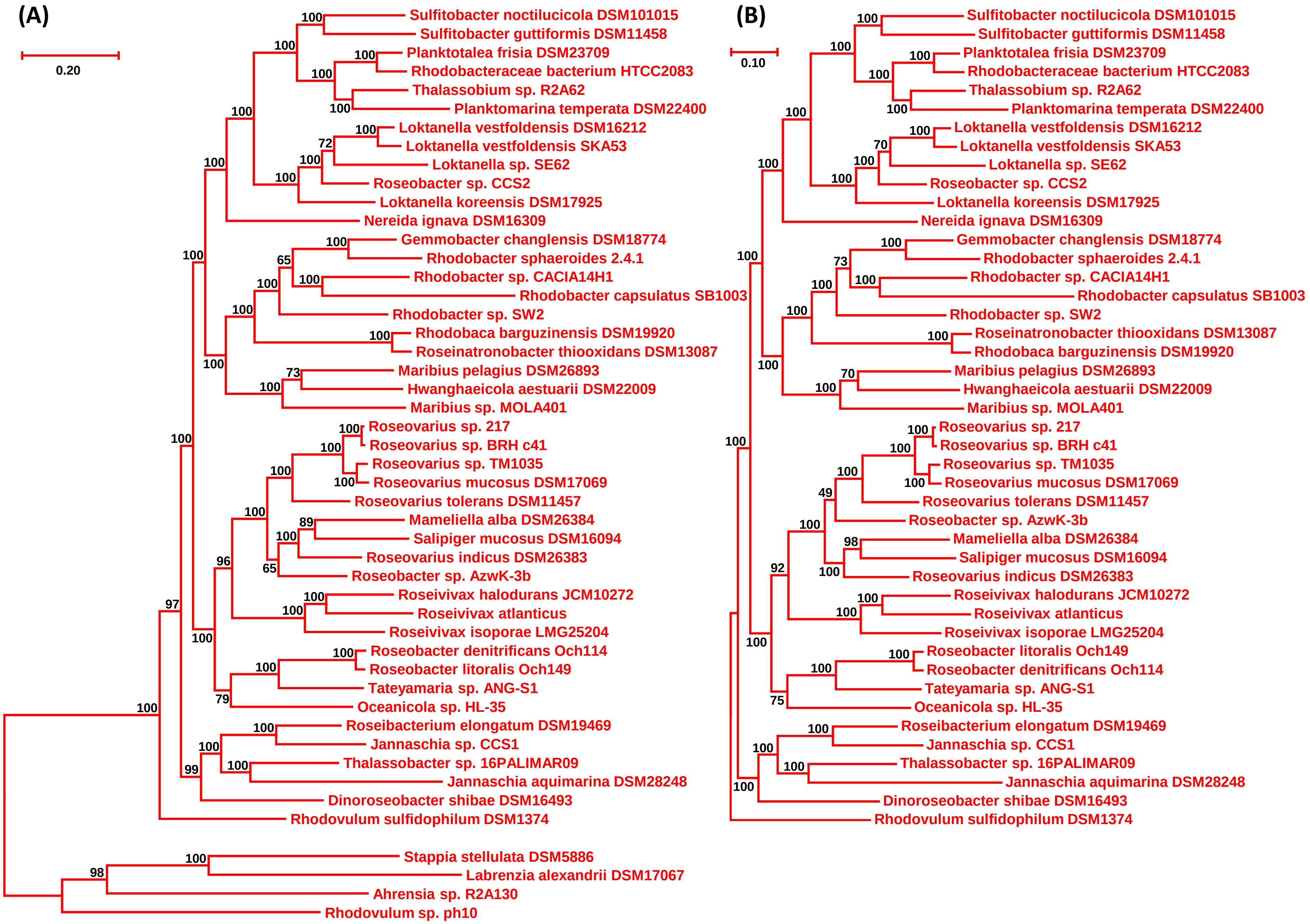

Figure S2. Maximum likelihood tree of 33 concatenated protein markers of the photosynthesis gene cluster (PGC). The phylogenetic inference was performed with RAxML v8.2.4 under a LGF4Γ model. The statistical support of the internal nodes was estimated with 100 bootstrap replicates by the rapid bootstrap option with the same model. According to the color code of the current study, PGC-trees are shown in red. **(A)** PGC tree of 44 *Rhodobacteraceae* and four alphaproteobacterial outgroup taxa based on 10,971 amino acid positions after g-blocks. **(B)** PGC tree of 44 *Rhodobacteraceae* based on 11,225 amino acid positions after g-blocks. The tree was rooted with *Rhodovulum sulfidophilum* DSM 1374 according to the branching pattern in Fig. S2A.
