# Supplementary material for: Horizontal operon transfer, plasmids, and the evolution of photosynthesis in Rhodobacteraceae
Source: ISME J. 2018 May 24;12(8):1994–2010. doi: 10.1038/s41396-018-0150-9 (PMC6052148; doi:10.1038/s41396-018-0150-9)
Supplement: Supplementary file 16 — Figure S3 [file 41396_2018_150_MOESM16_ESM.pdf]

Figure S3-01 - crtA #04

Phylogenetic RaxML Analysis (LGF4Γ; 100 bootstrap replicates)

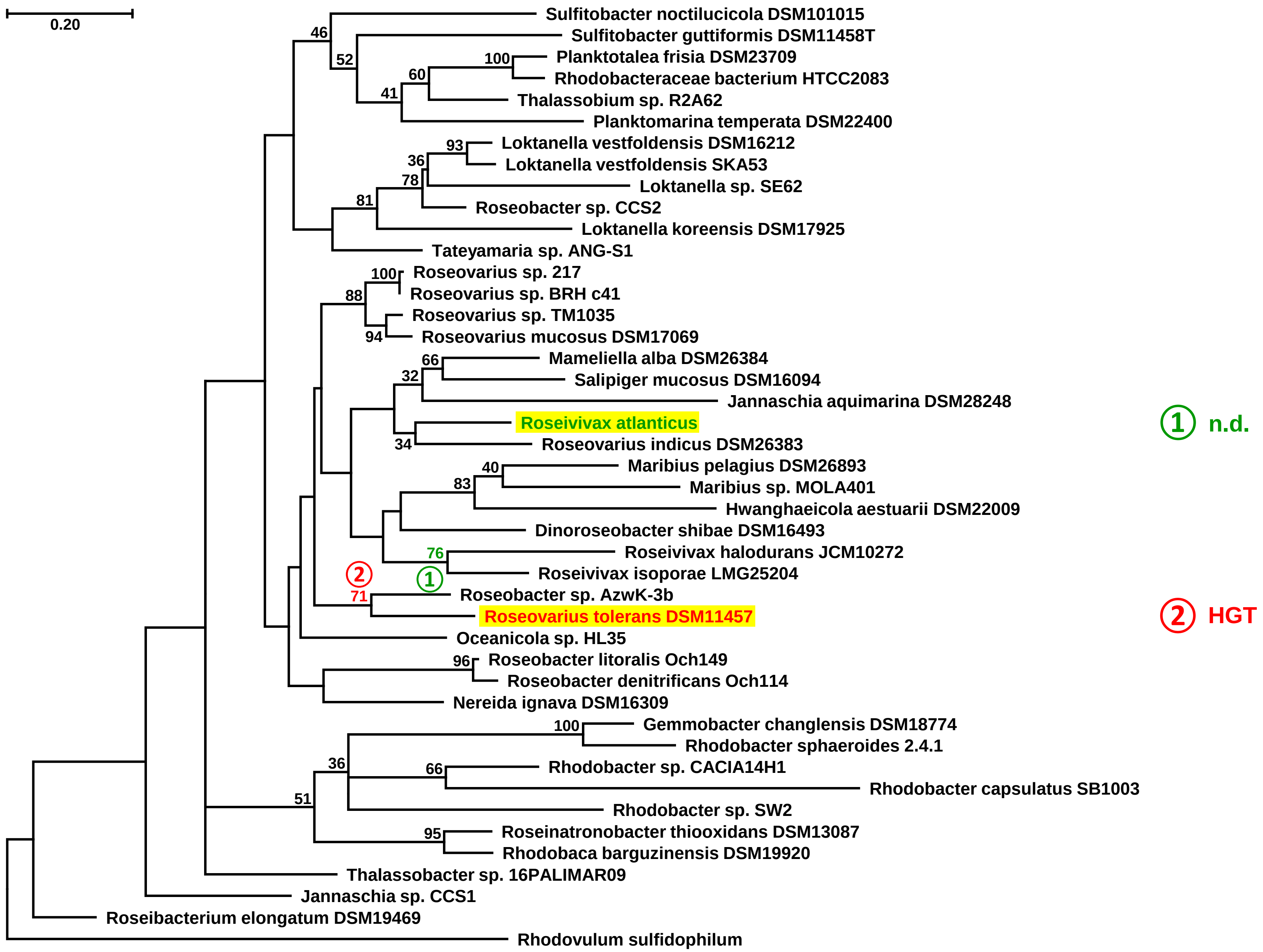

Figure S3-02 - bchI #05 Phylogenetic RaxML Analysis (LGF4Γ; 100 bootstrap replicates)

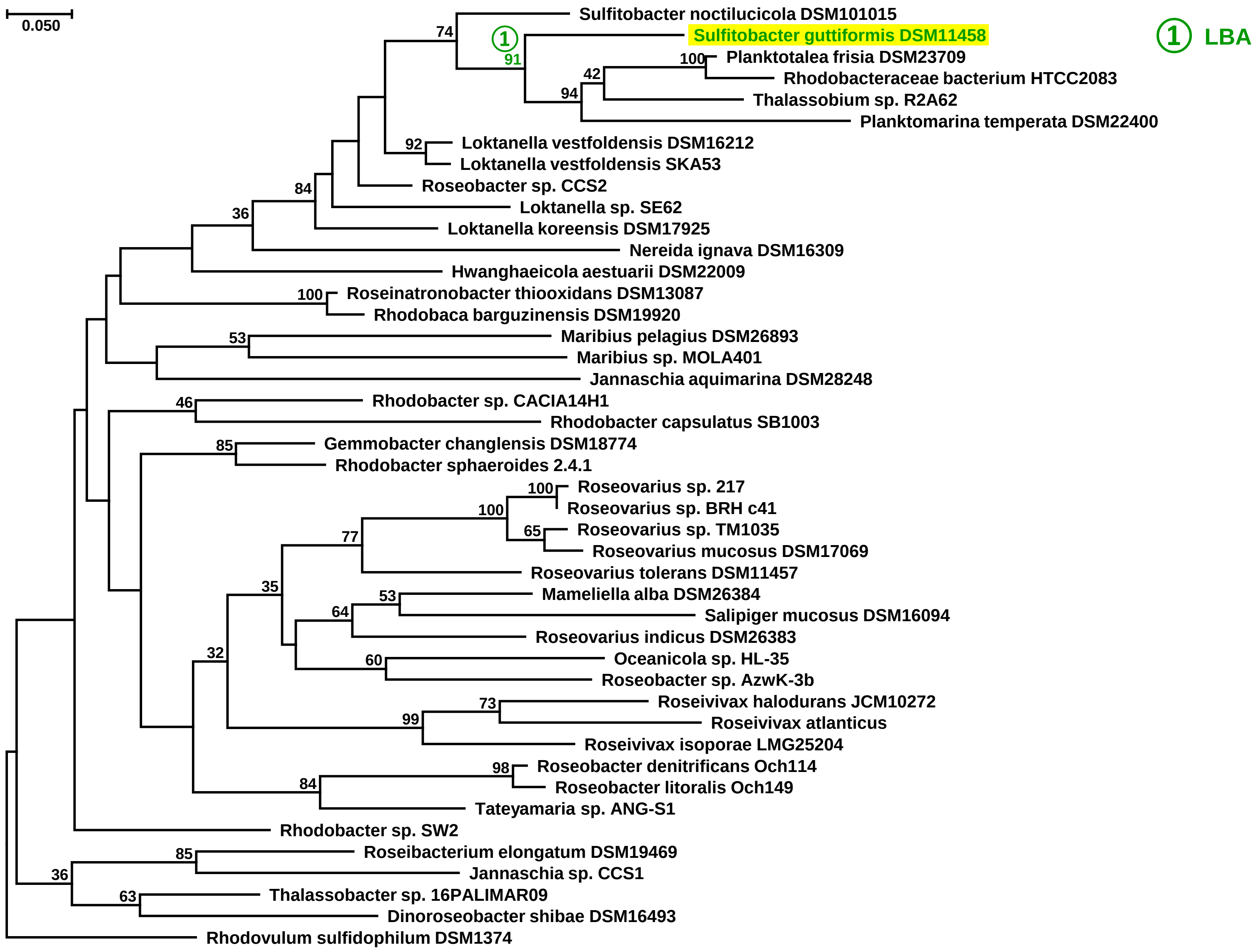

Figure S3-03 - bchD #06 Phylogenetic RaxML Analysis (LGF4Γ; 100 bootstrap replicates)

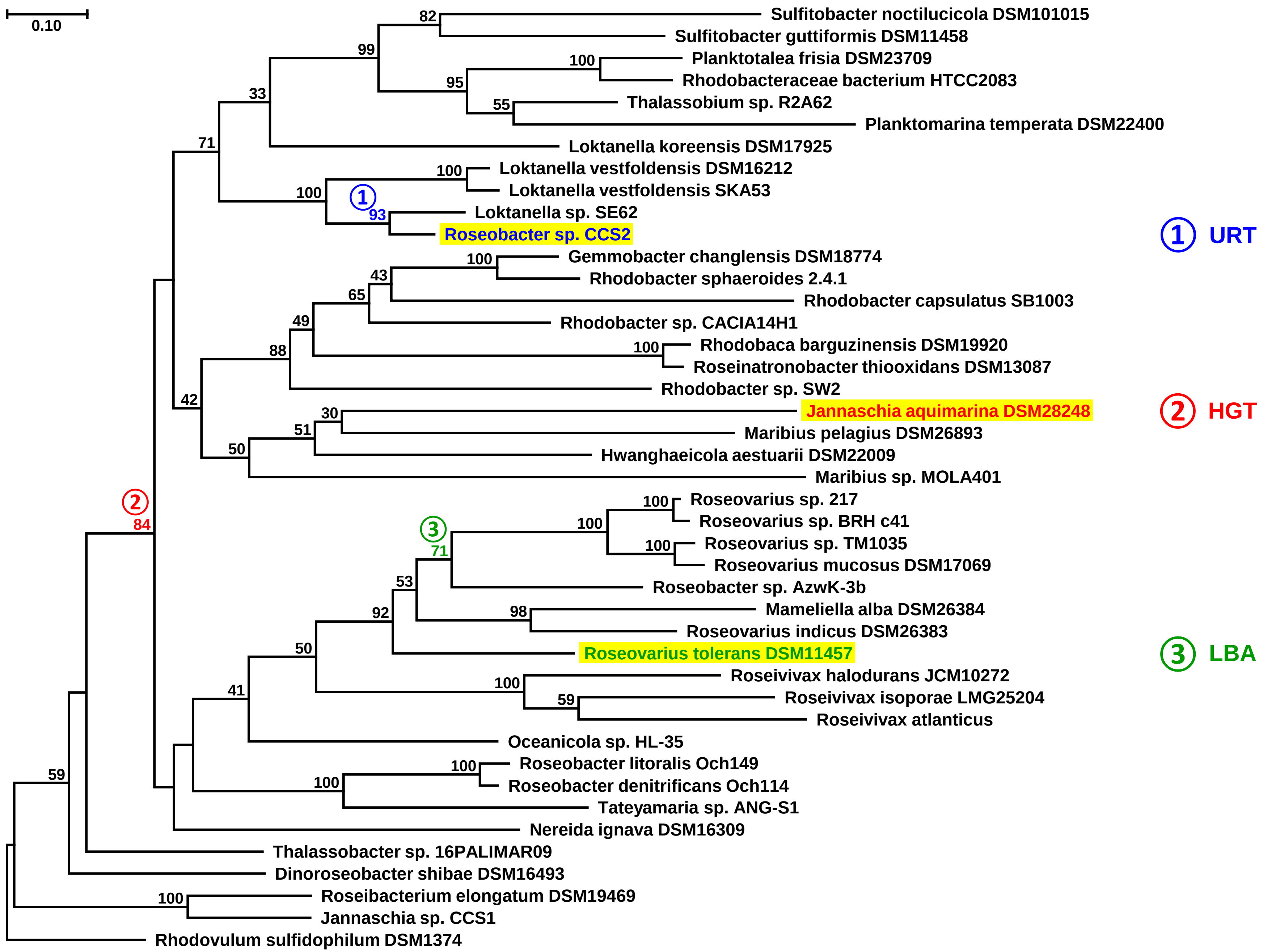

Figure S3-04 - bchO #07 **Phylogenetic RaxML Analysis** (LGF4Γ; 100 bootstrap replicates)

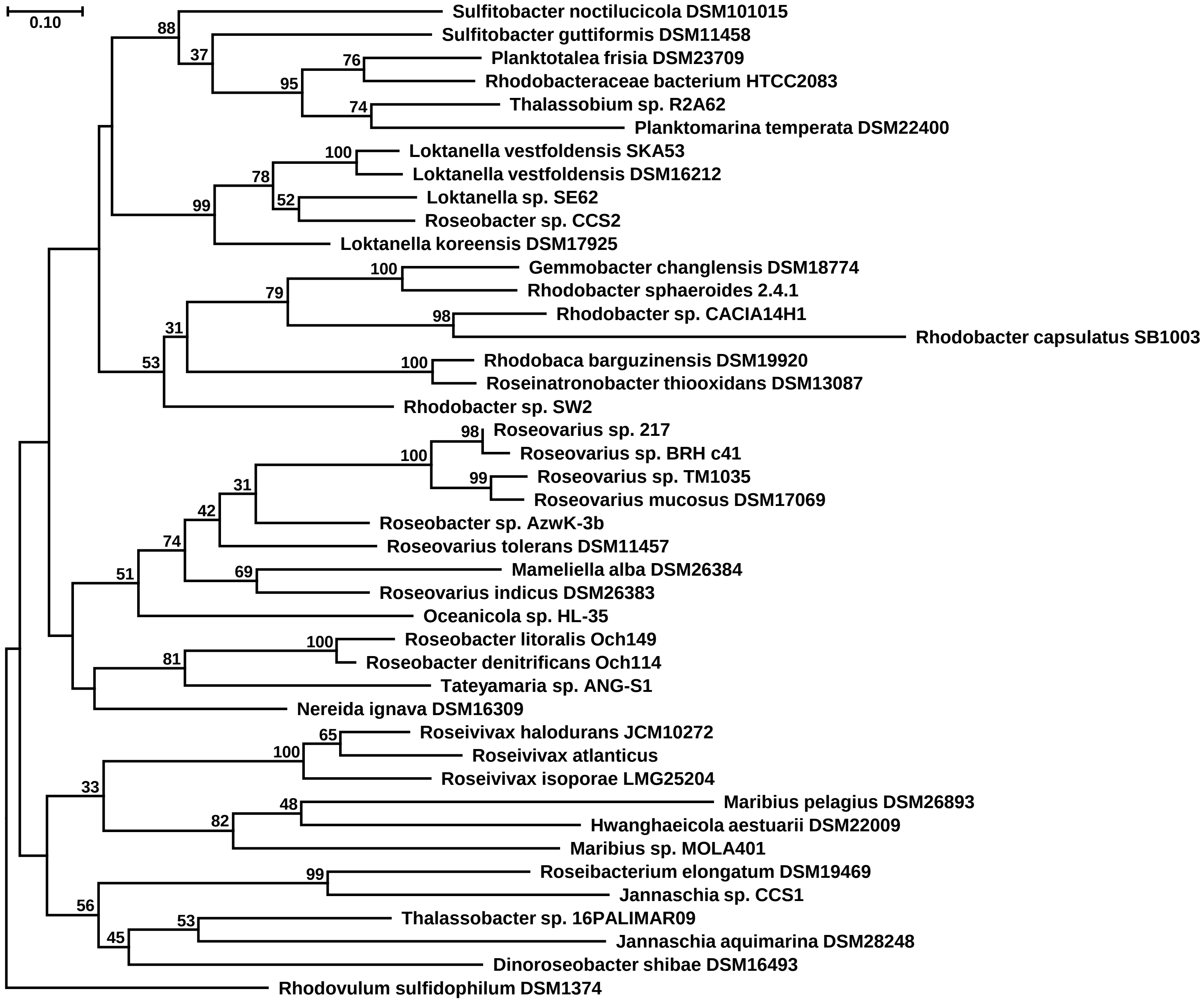

Figure S3-05 - crtI #11

Phylogenetic RaxML Analysis (LGF4Γ; 100 bootstrap replicates)

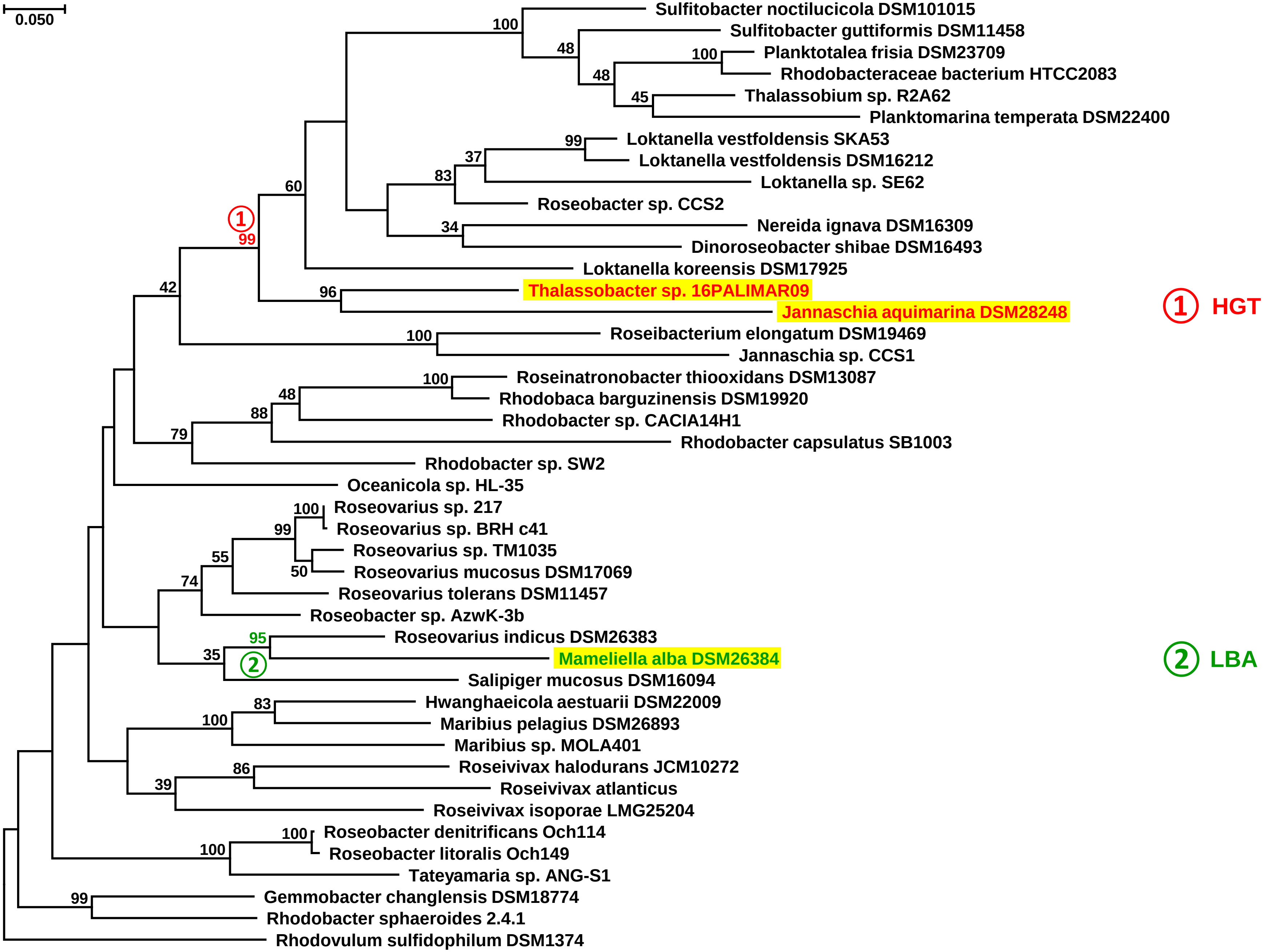

Figure S3-06 - crtB #12

Phylogenetic RaxML Analysis (LGF4Γ; 100 bootstrap replicates)

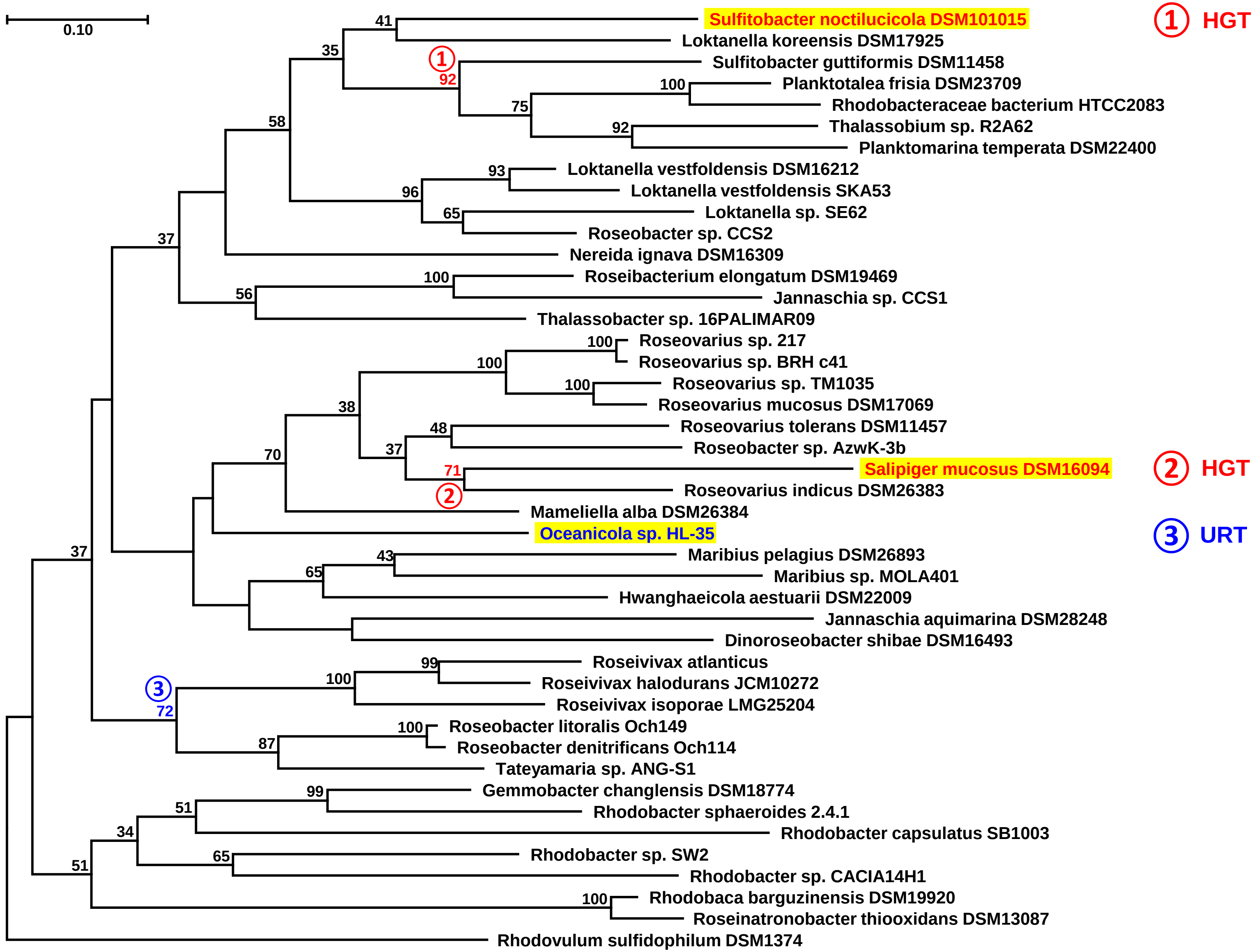

Figure S3-07 - crtK #13 **Phylogenetic RaxML Analysis** (LGF4Γ; 100 bootstrap replicates)

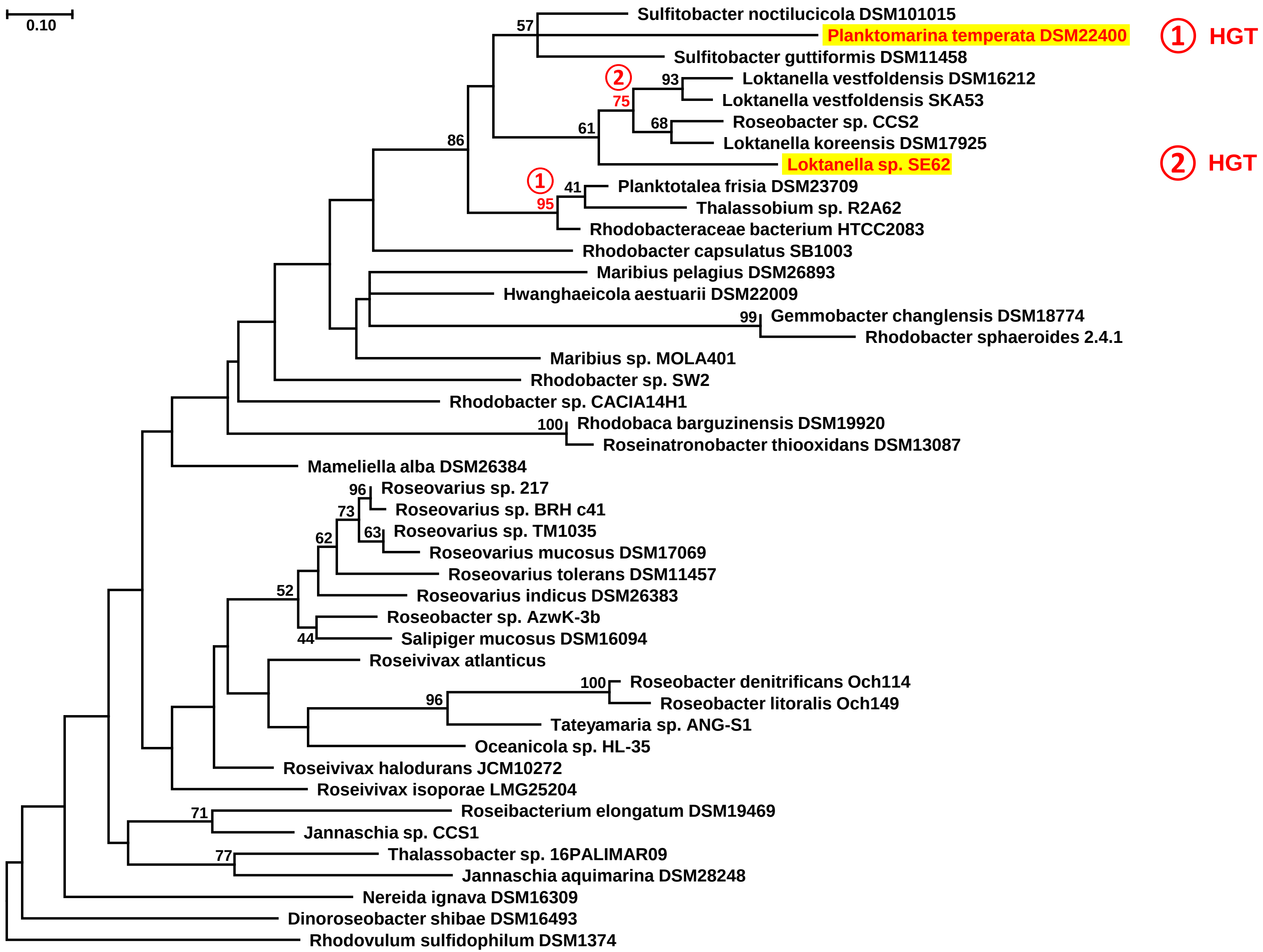

Figure S3-08 - crtC #15

Phylogenetic RaxML Analysis (LGF4Γ; 100 bootstrap replicates)

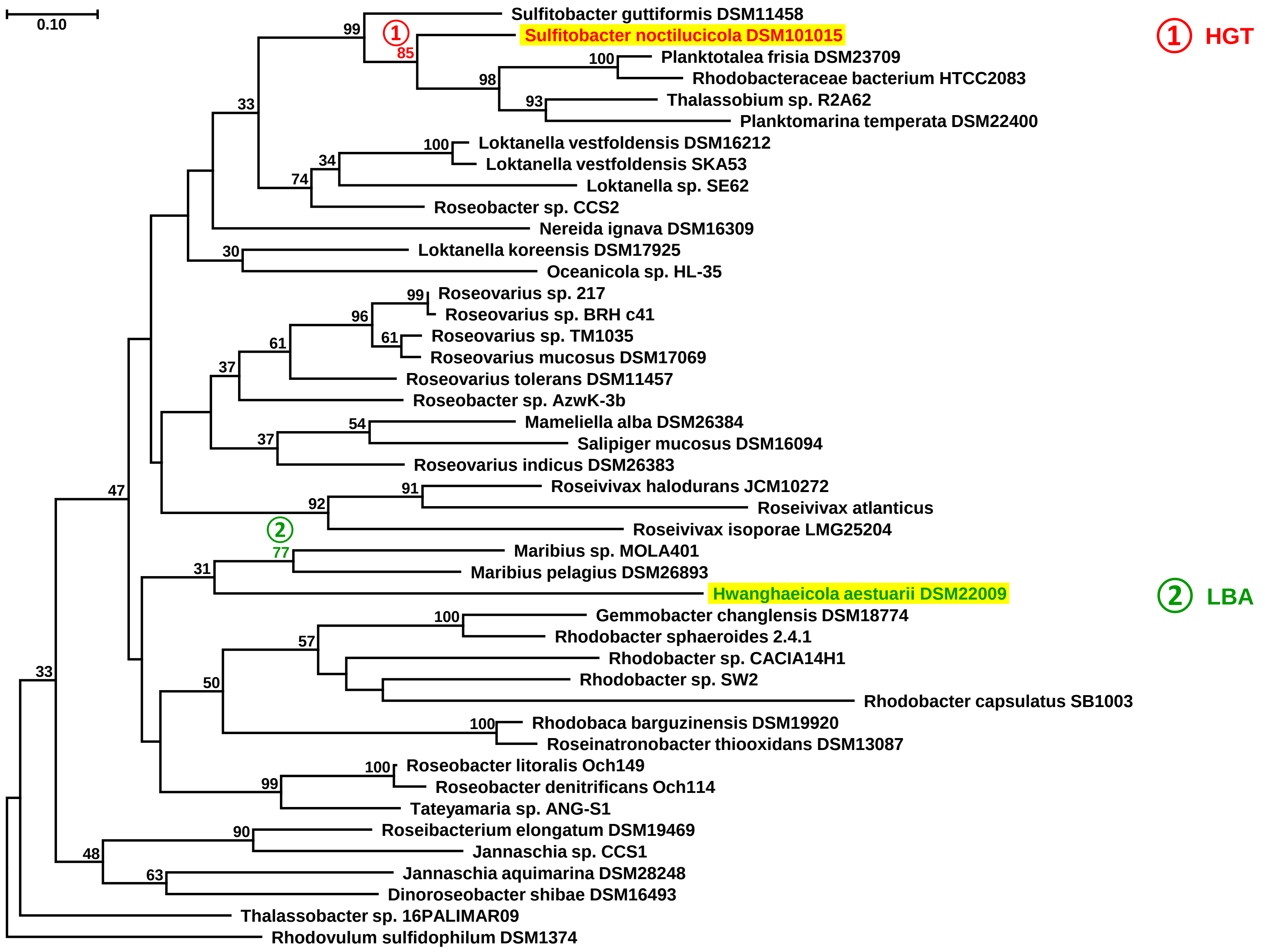

Figure S3-09 - crtD #16

Phylogenetic RaxML Analysis (LGF4Γ; 100 bootstrap replicates)

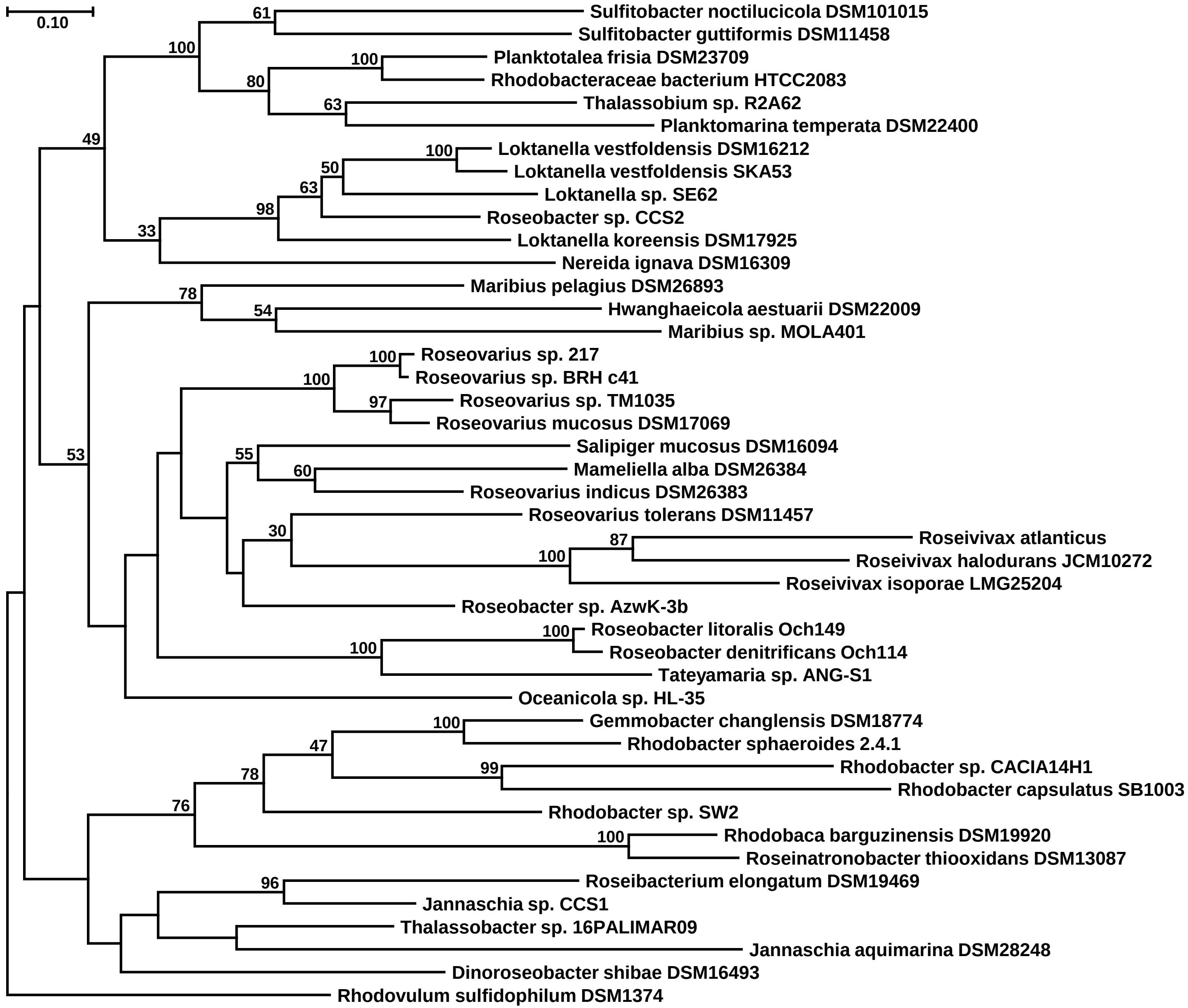

Figure S3-10 - crtE #17 **Phylogenetic RaxML Analysis** (LGF4Γ; 100 bootstrap replicates)

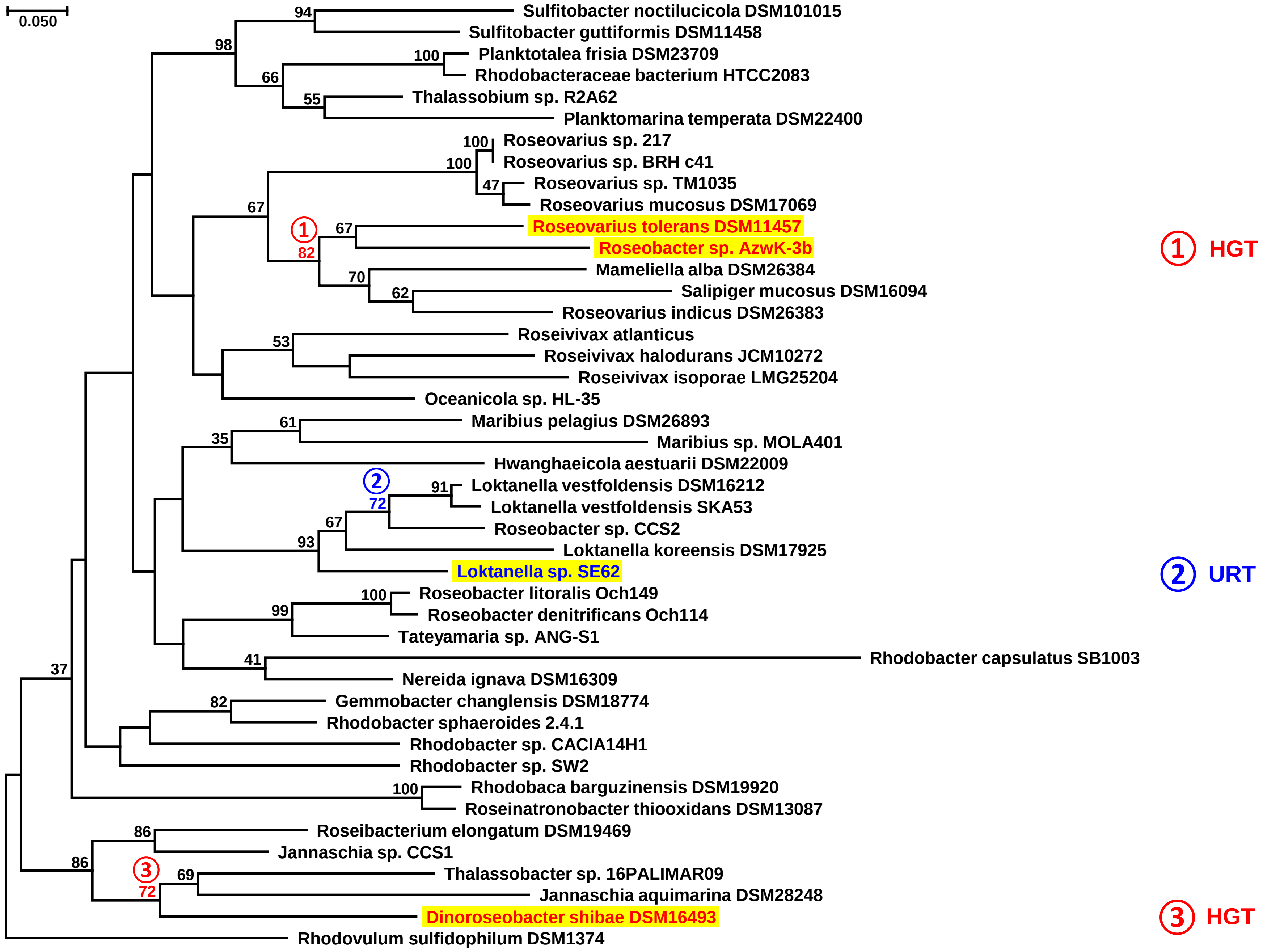

Figure S3-11 - crtF #18

Phylogenetic RaxML Analysis (LGF4Γ; 100 bootstrap replicates)

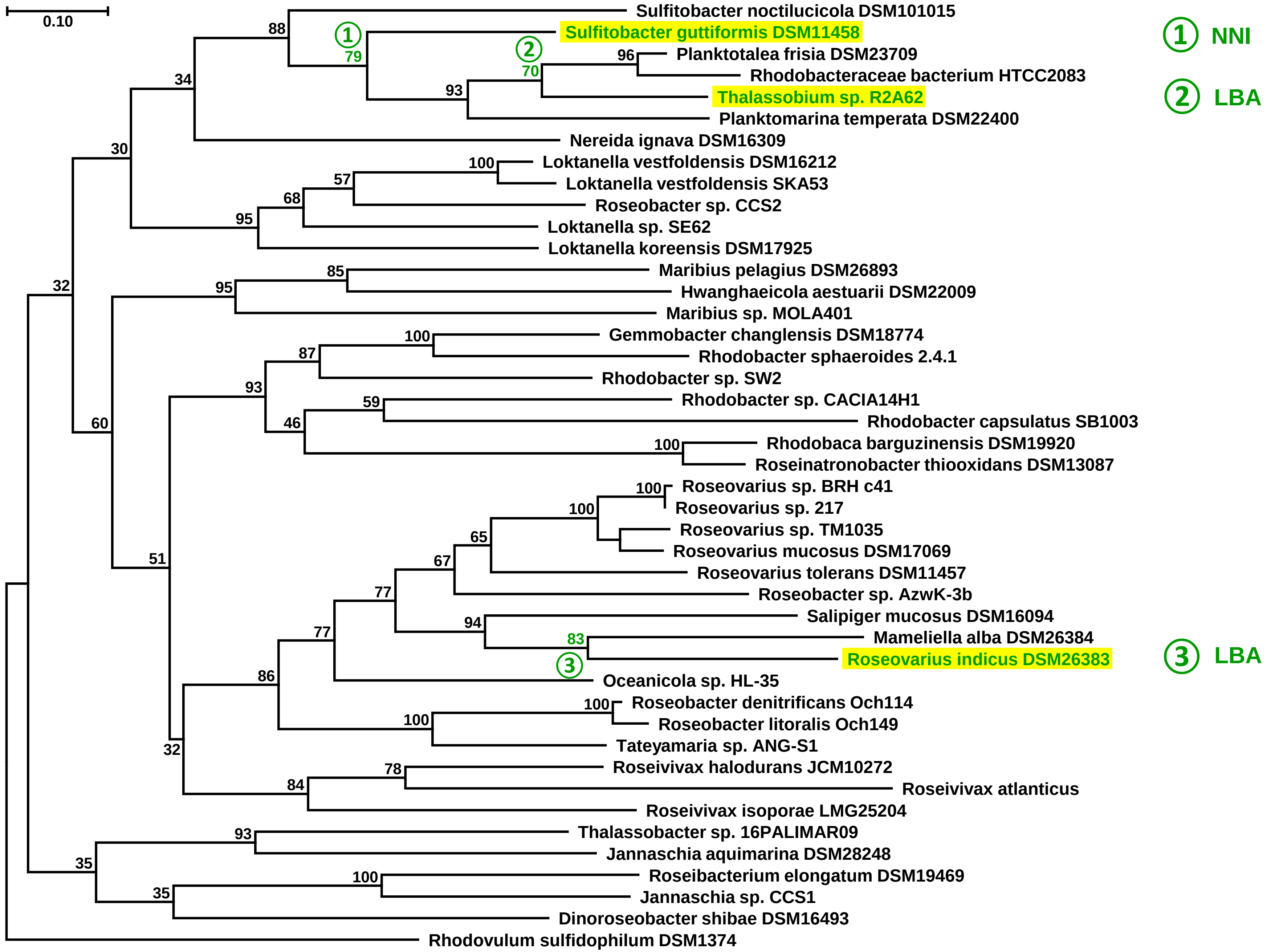

Figure S3-12 - bchC #19 **Phylogenetic RaxML Analysis** (LGF4Γ; 100 bootstrap replicates)

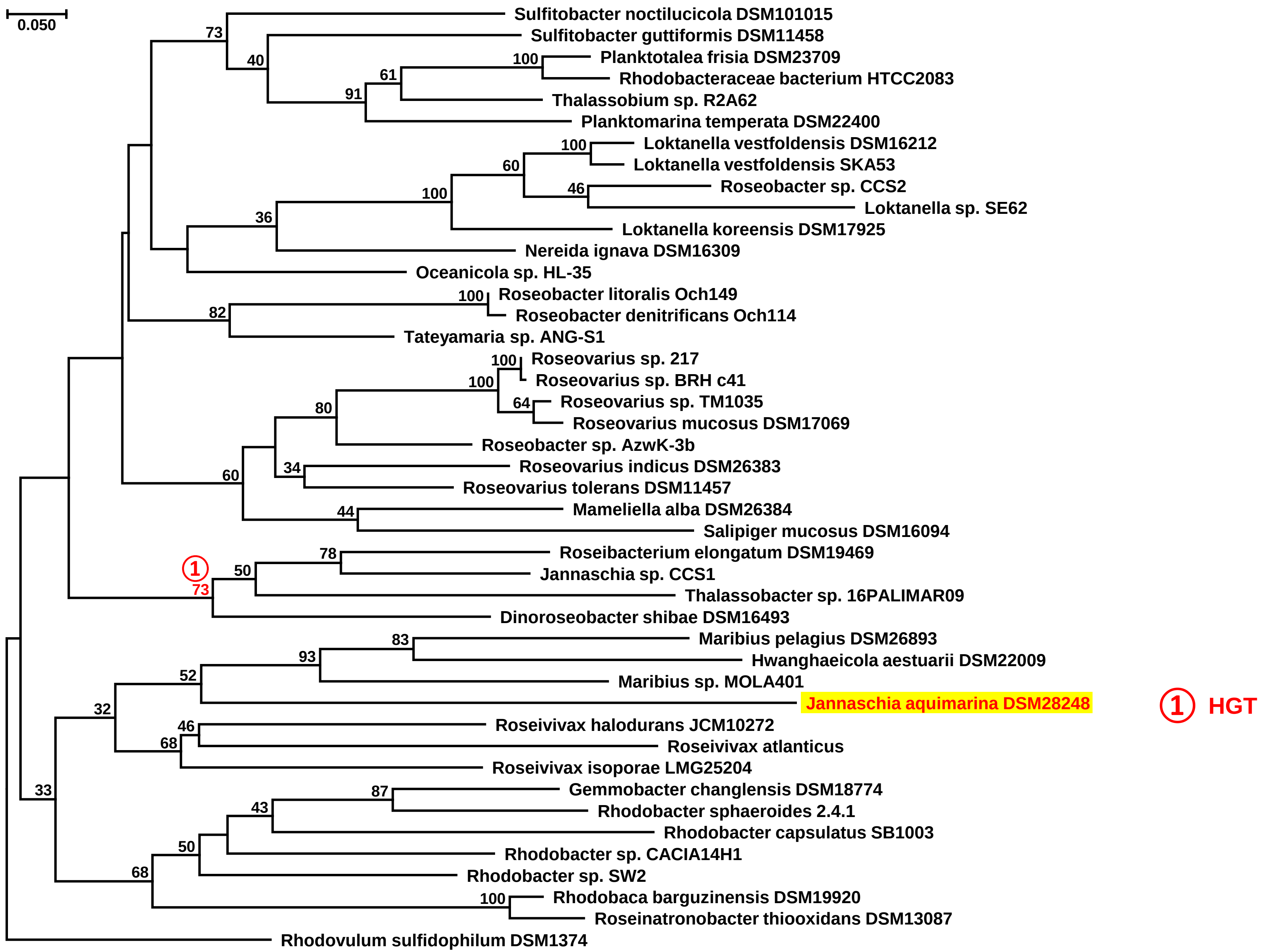

Figure S3-13 - bchX #20 **Phylogenetic RaxML Analysis** (LGF4Γ; 100 bootstrap replicates)

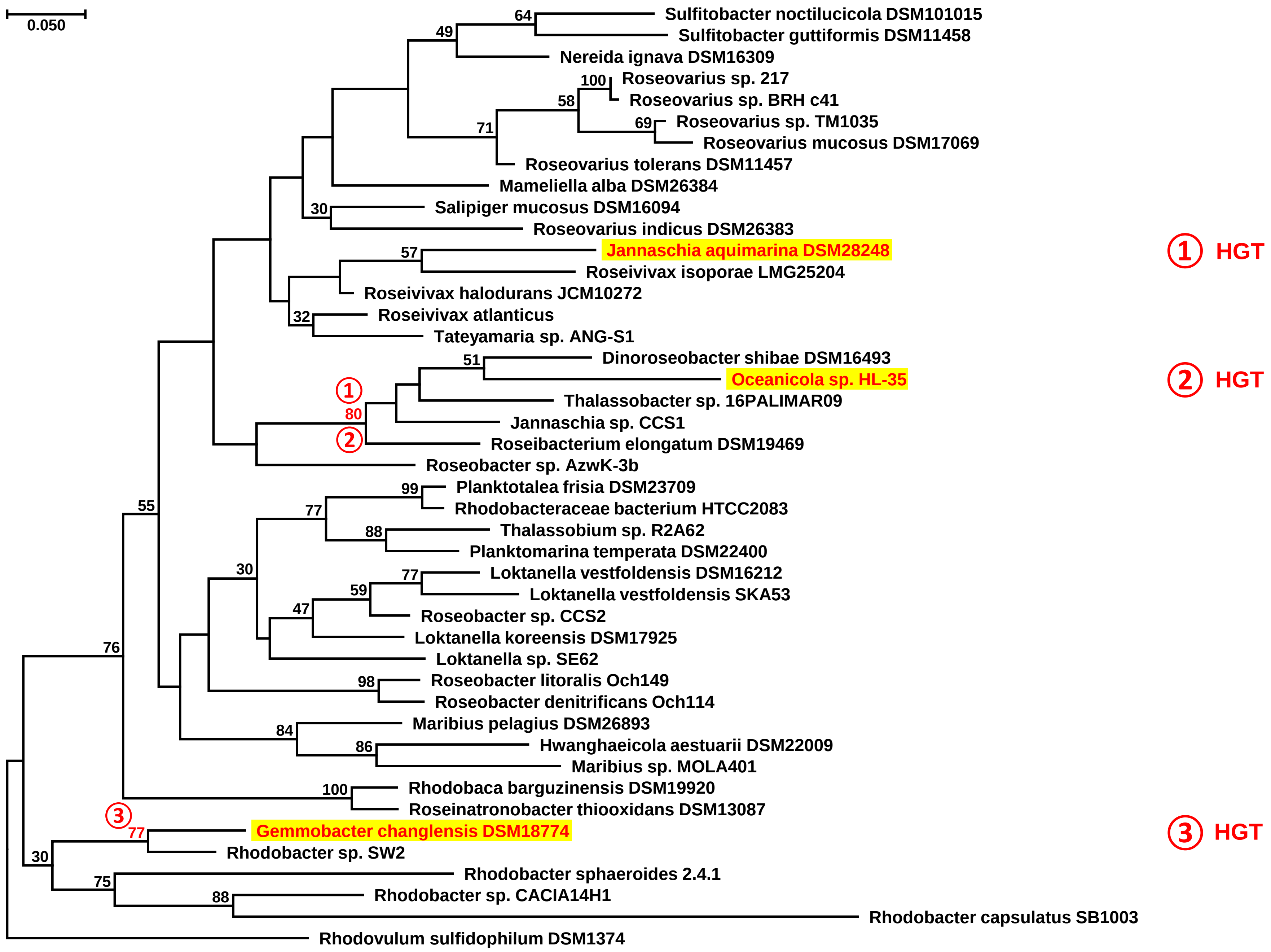

Figure S3-14 - bchY #21 **Phylogenetic RaxML Analysis** (LGF4Γ; 100 bootstrap replicates)

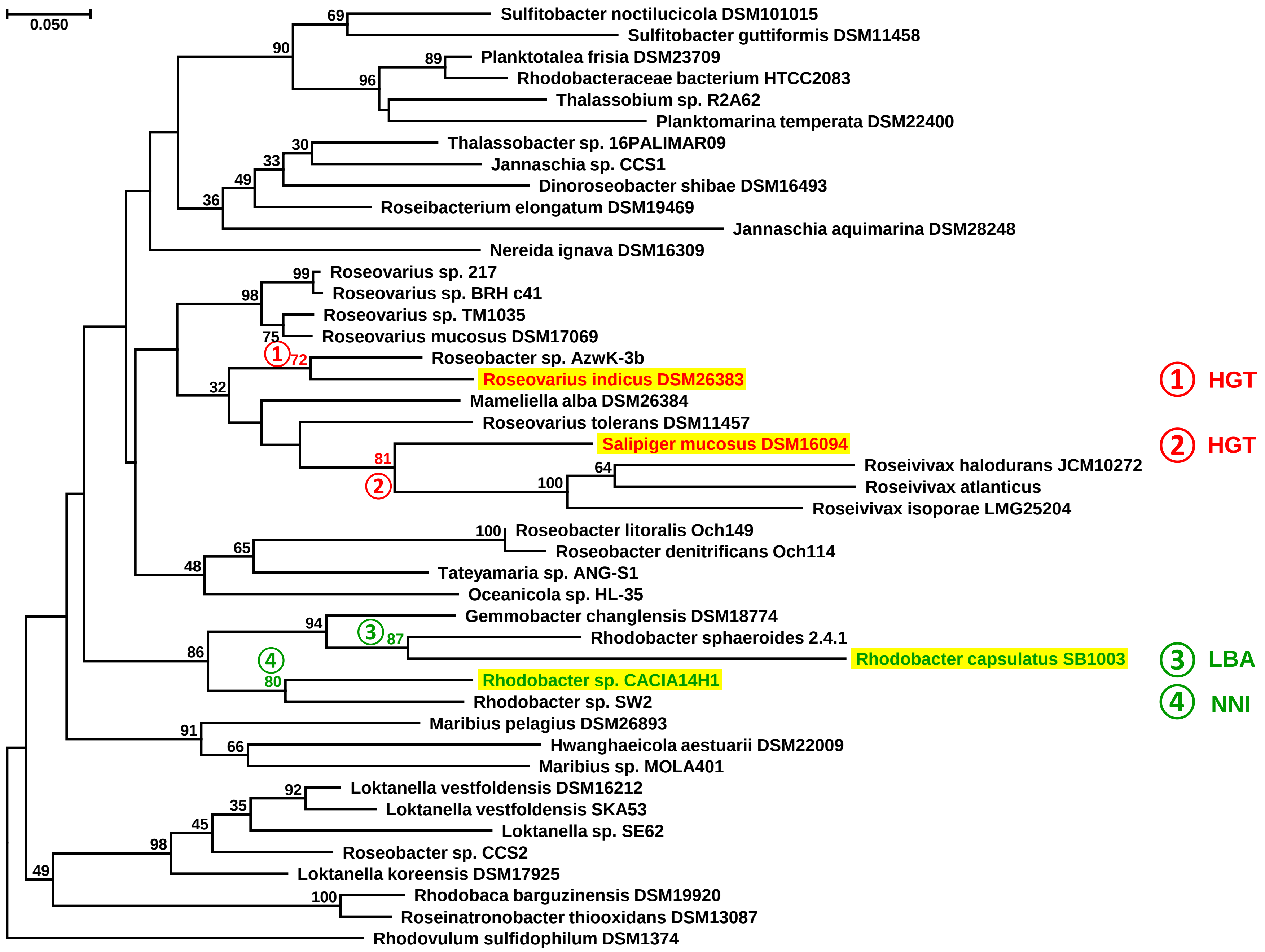

Figure S3-15 - bchZ #22

Phylogenetic RaxML Analysis (LGF4Γ; 100 bootstrap replicates)

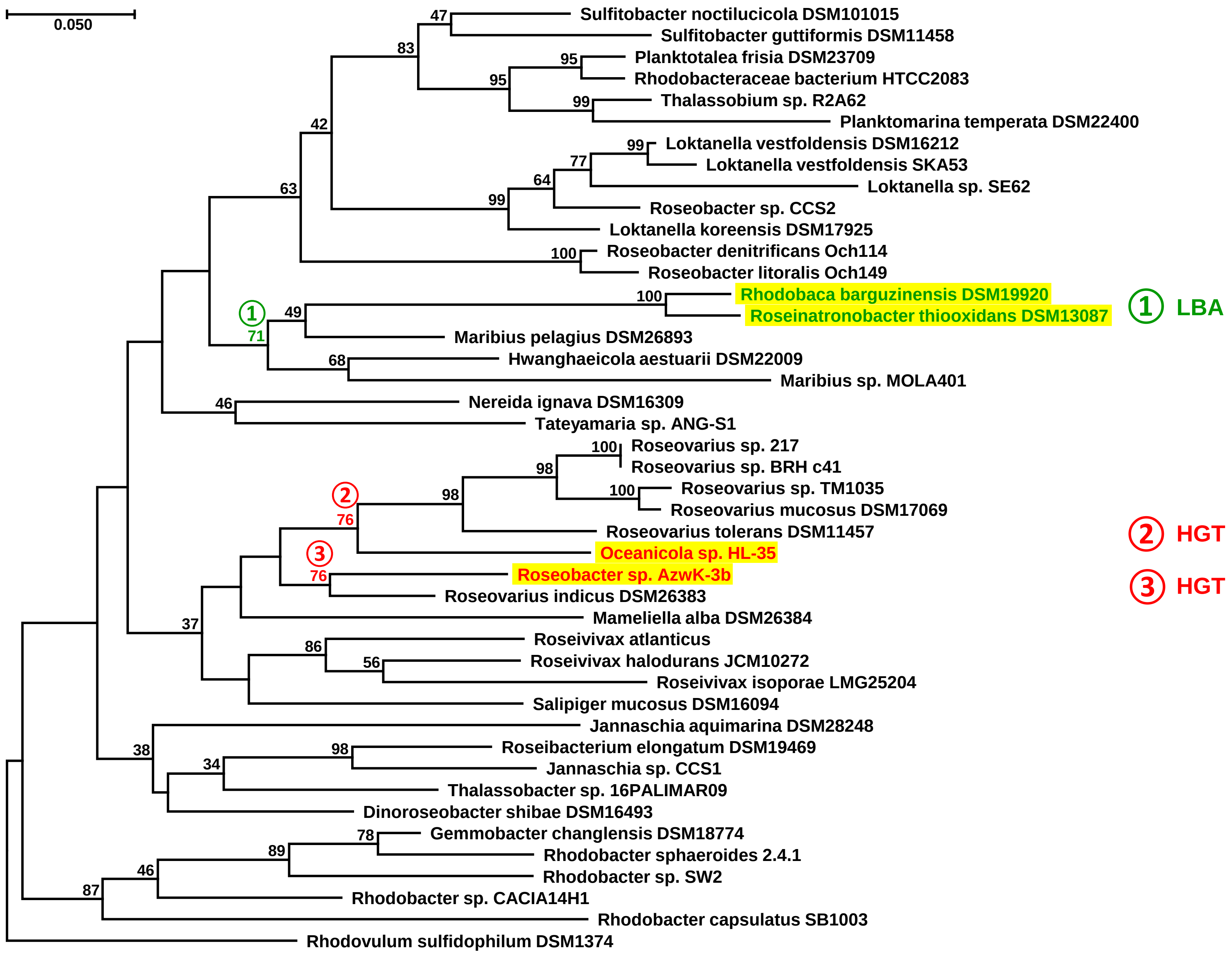

Figure S3-16 - pufL #26

Phylogenetic RaxML Analysis (LGF4Γ; 100 bootstrap replicates)

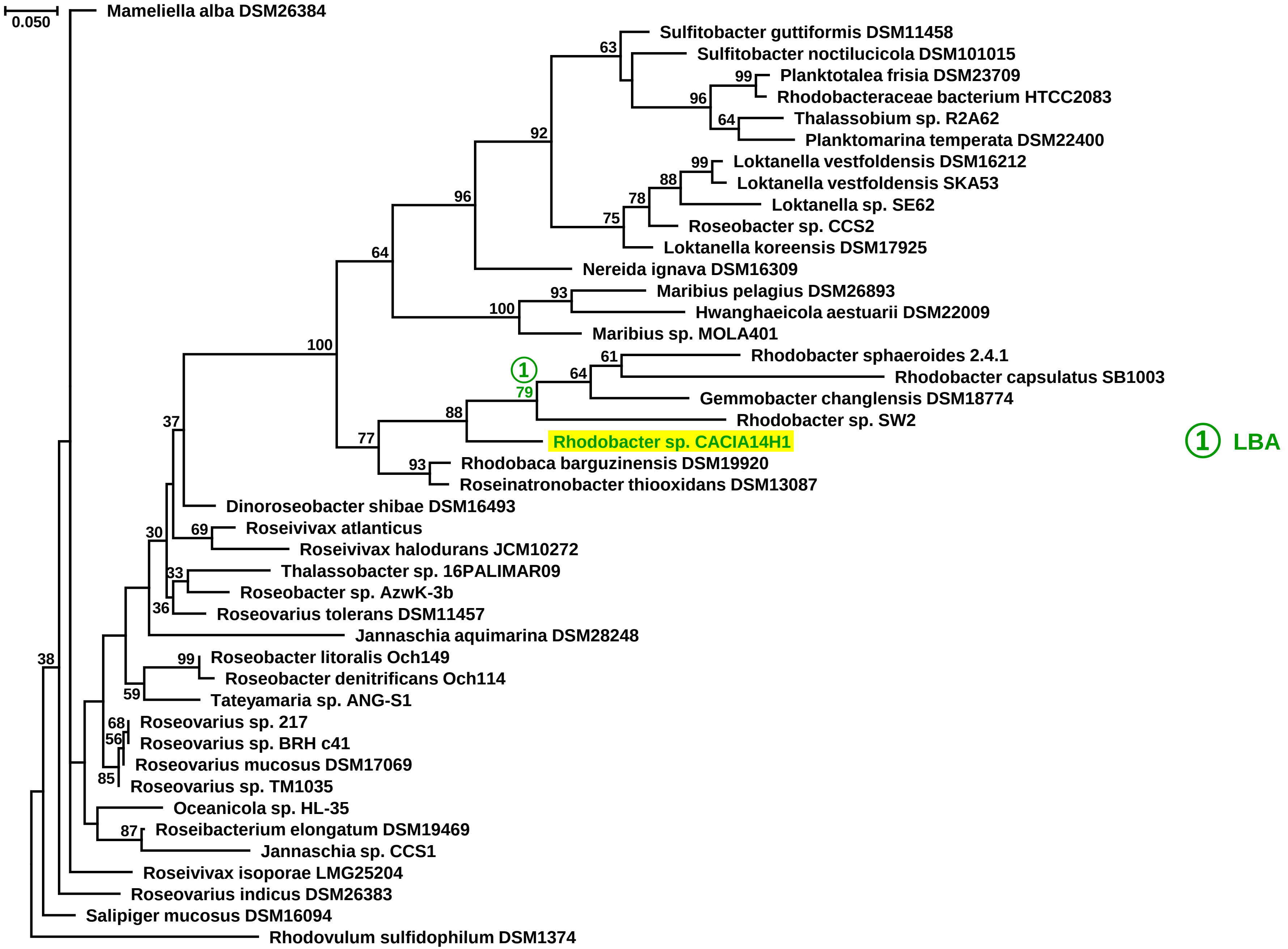

Figure S3-17 - pufM #27 **Phylogenetic RaxML Analysis** (LGF4Γ; 100 bootstrap replicates)

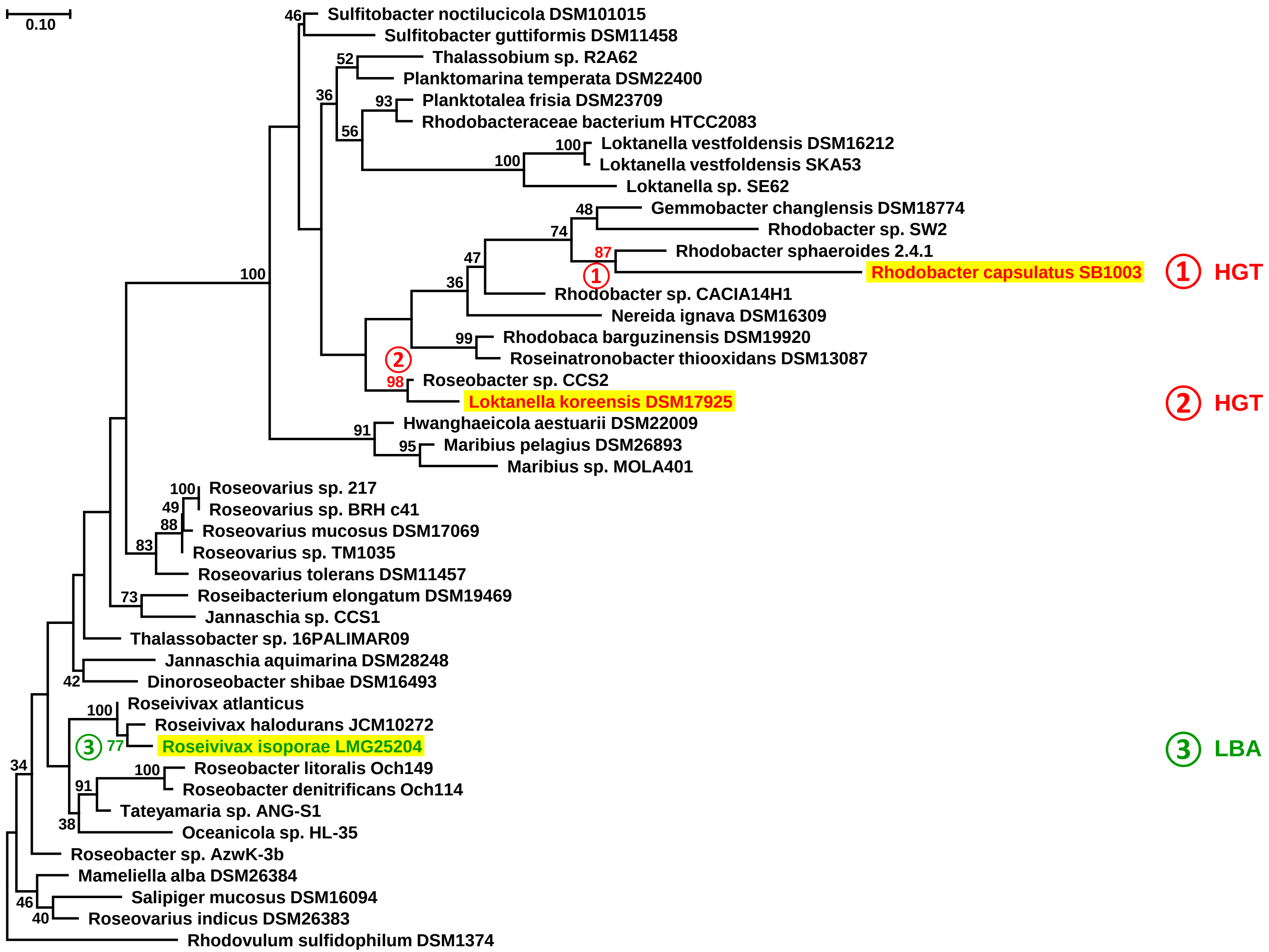

Figure S3-18 - dxps #29

Phylogenetic RaxML Analysis (LGF4Γ; 100 bootstrap replicates)

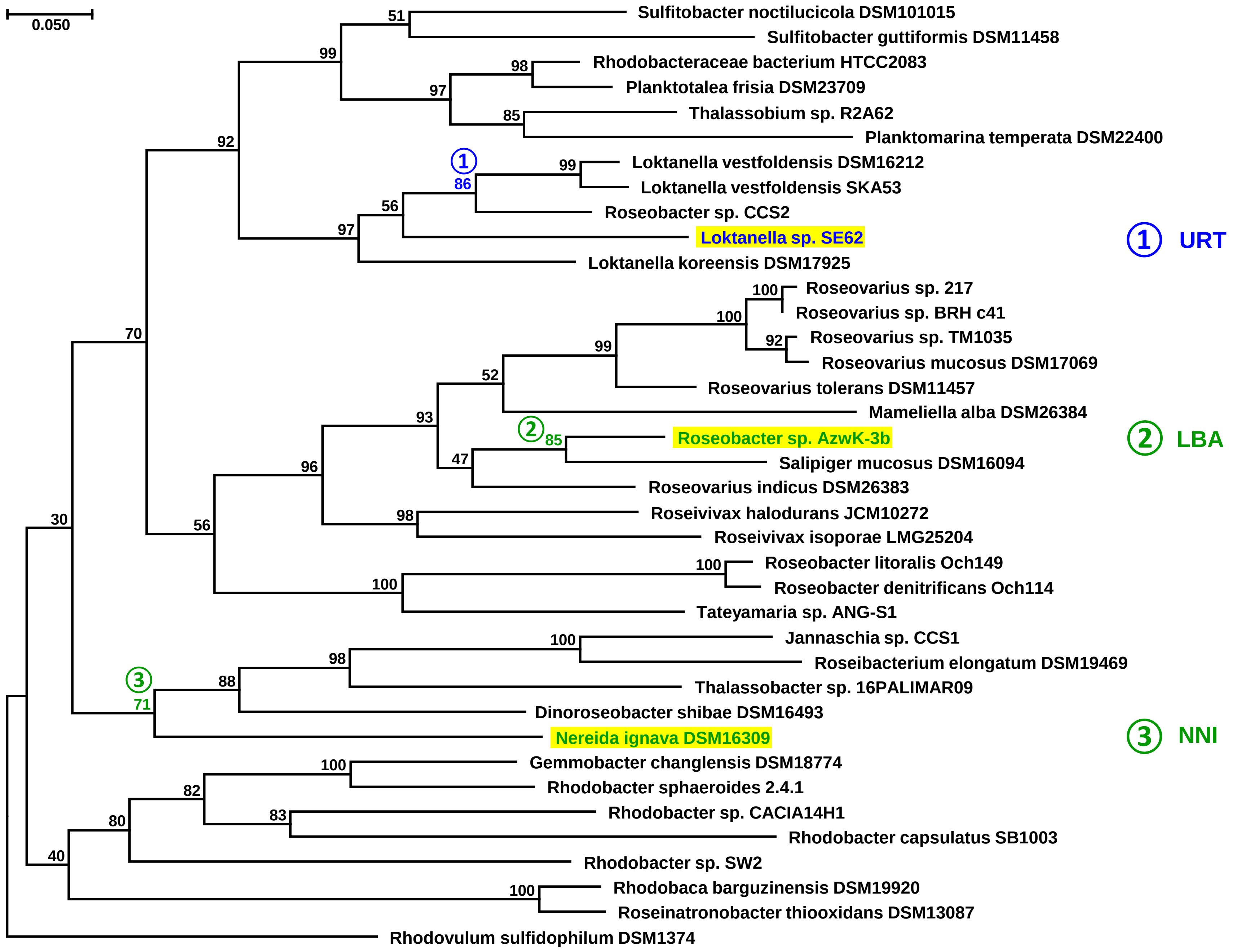

Figure S3-19 - bchP #31 Phylogenetic RaxML Analysis (LGF4Γ; 100 bootstrap replicates)

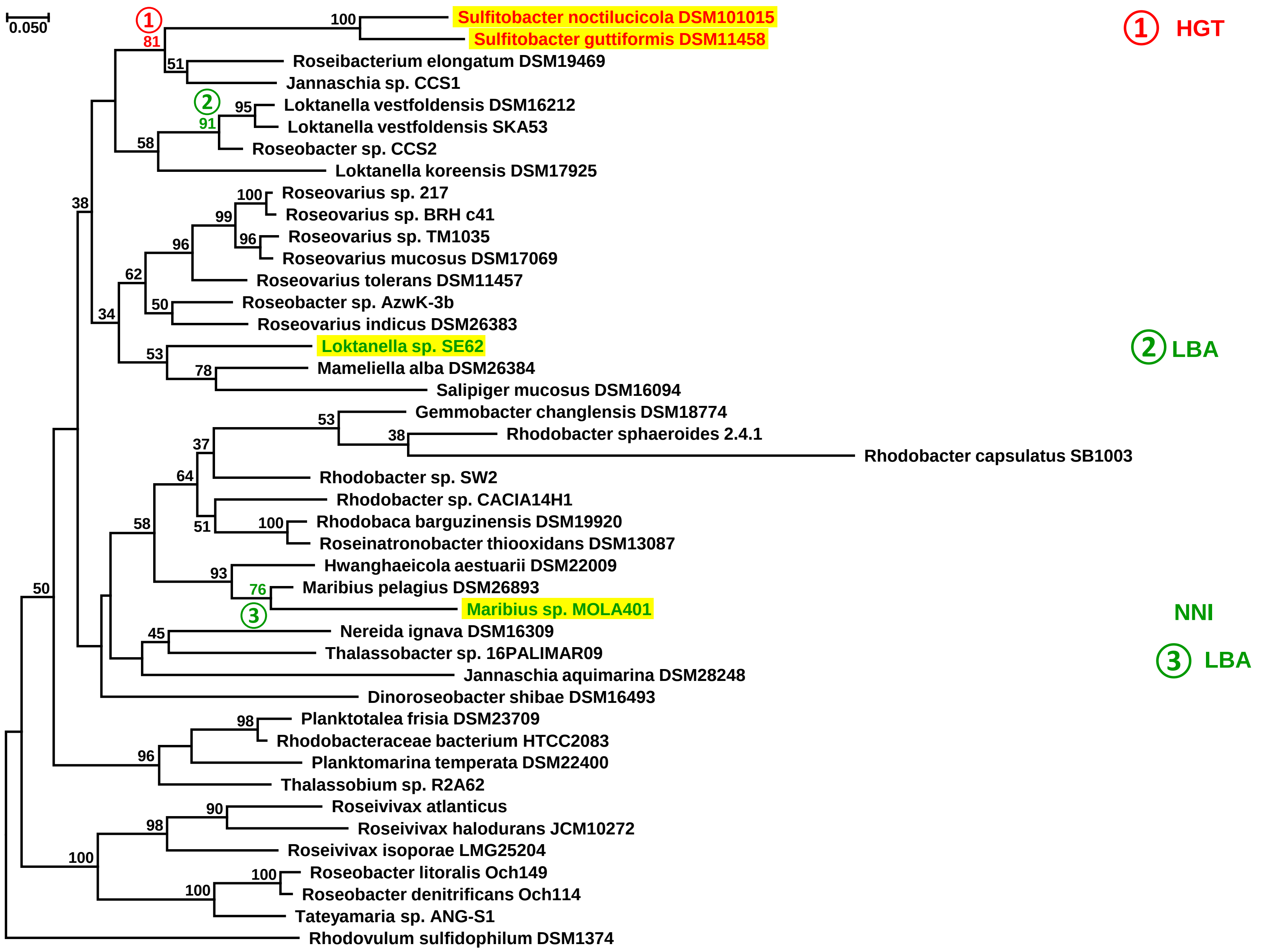

Figure S3-20 - pucC #32 **Phylogenetic RaxML Analysis** (LGF4Γ; 100 bootstrap replicates)

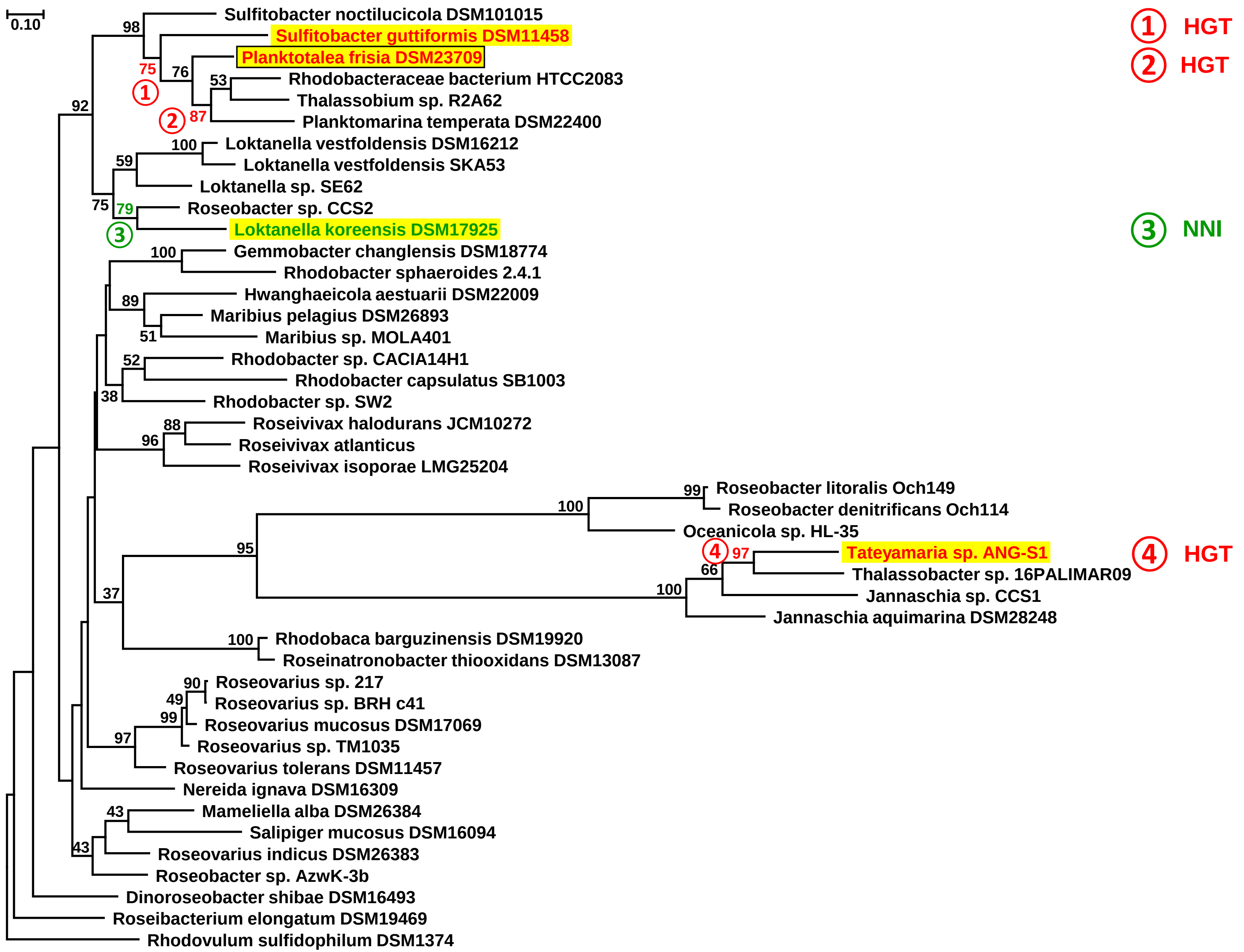

Figure S3-21 - bchG #33 **Phylogenetic RaxML Analysis** (LGF4Γ; 100 bootstrap replicates)

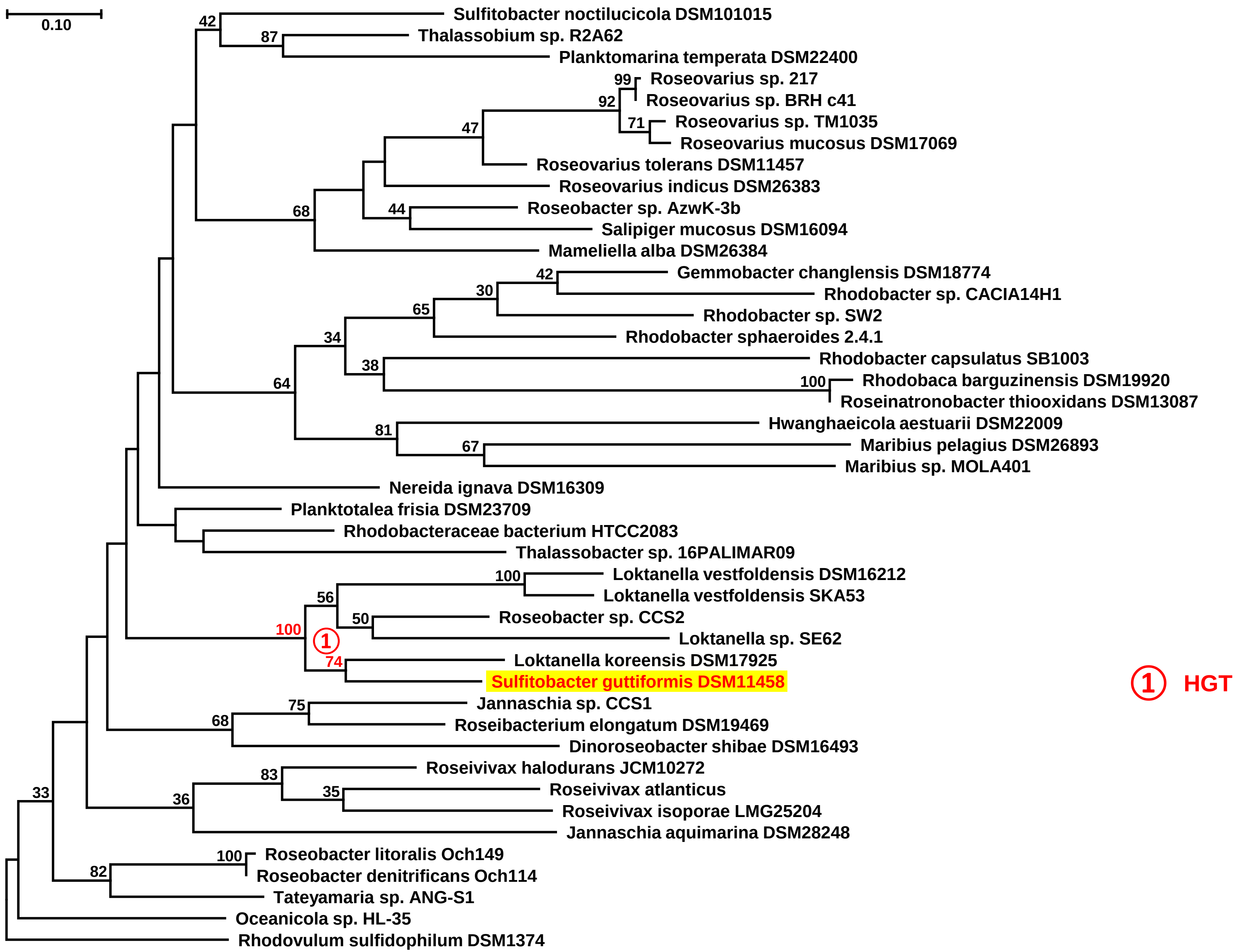

Figure S3-22 - ppsR #34 **Phylogenetic RaxML Analysis** (LGF4Γ; 100 bootstrap replicates)

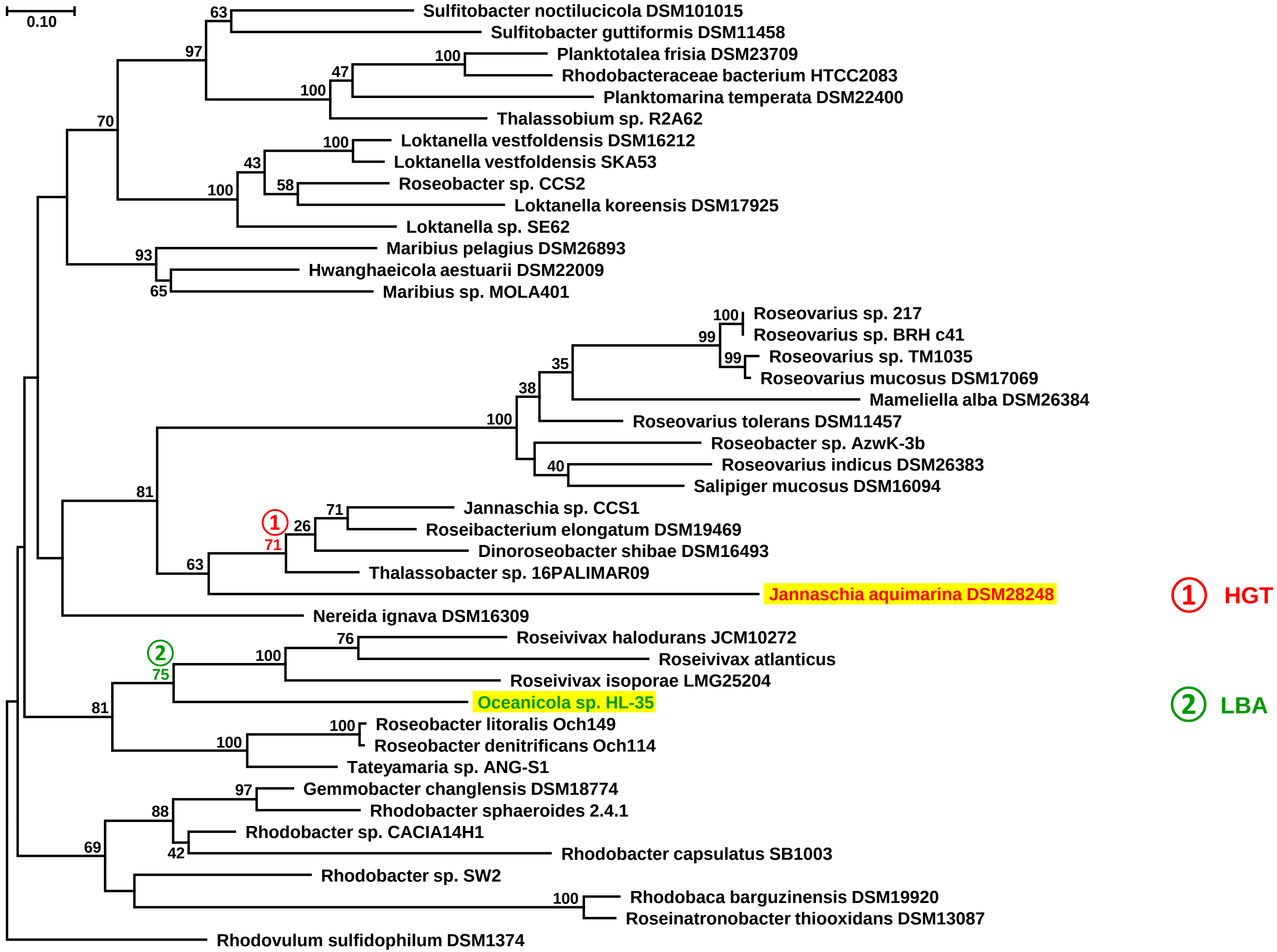

Figure S3-23 - bchE #36

Phylogenetic RaxML Analysis (LGF4Γ; 100 bootstrap replicates)

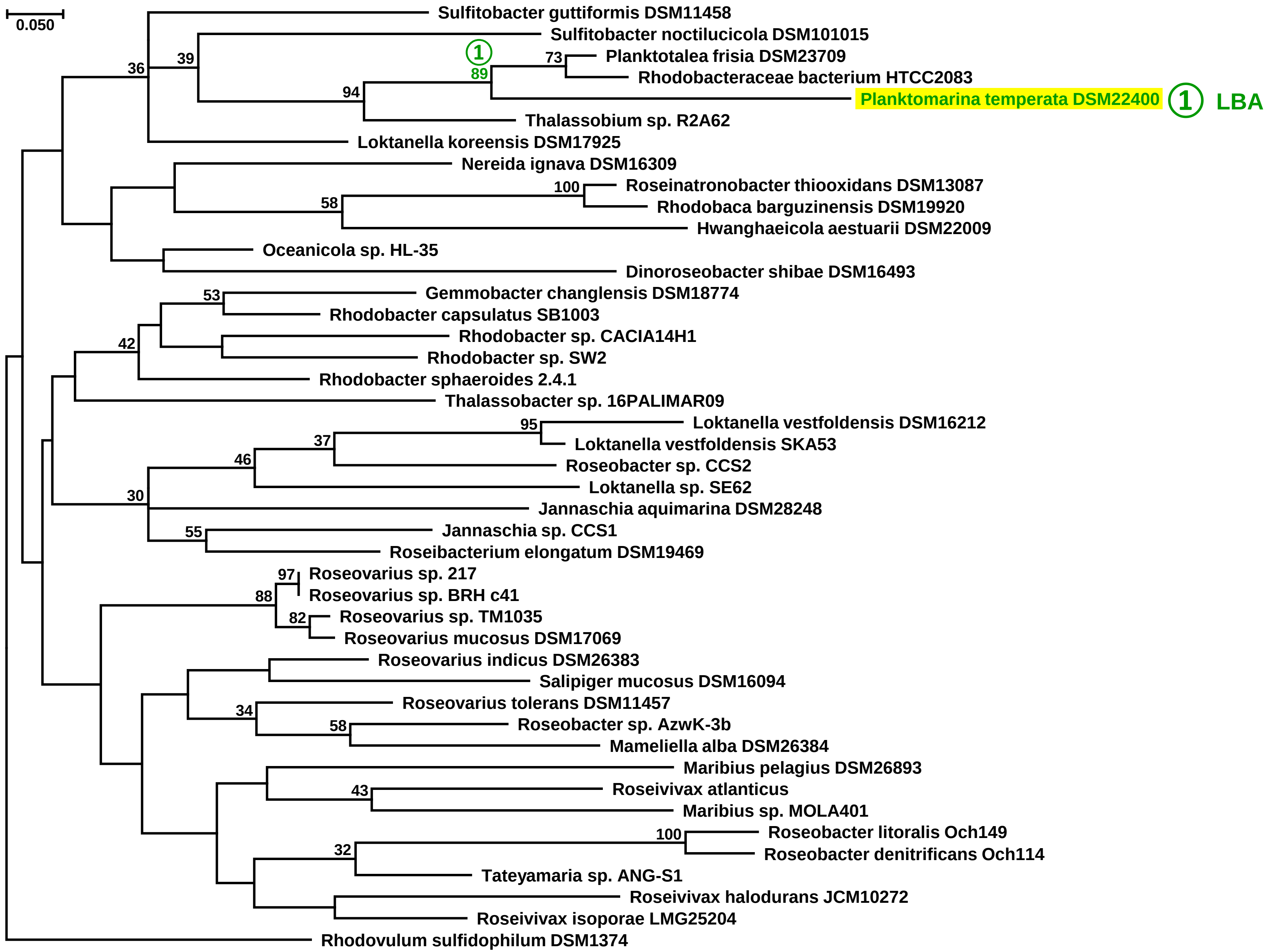

Figure S3-24 - bchN #37 **Phylogenetic RaxML Analysis** (LGF4Γ; 100 bootstrap replicates)

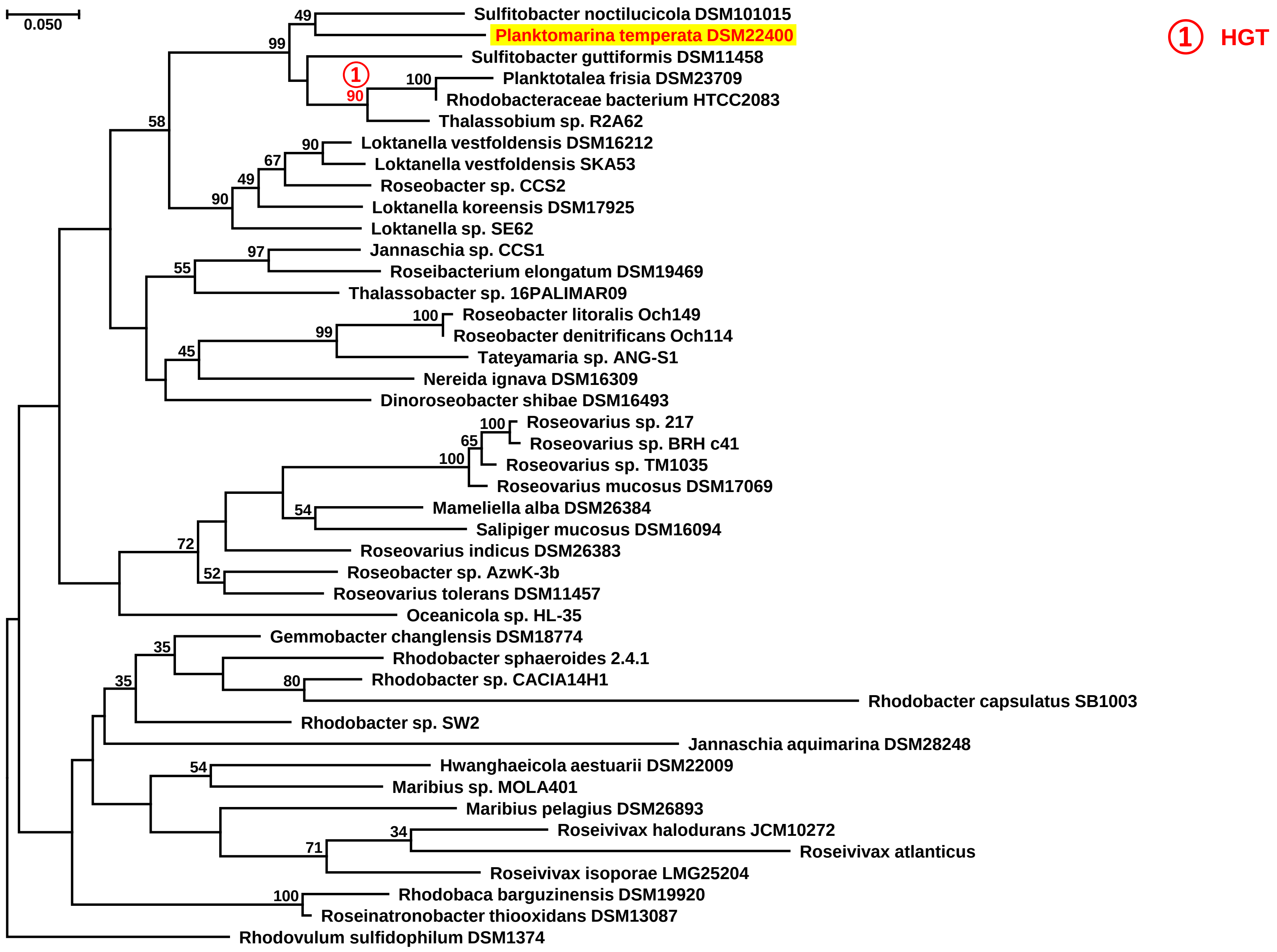

Figure S3-25 - bchB #38   Phylogenetic RaxML Analysis (LGF4Γ; 100 bootstrap replicates)

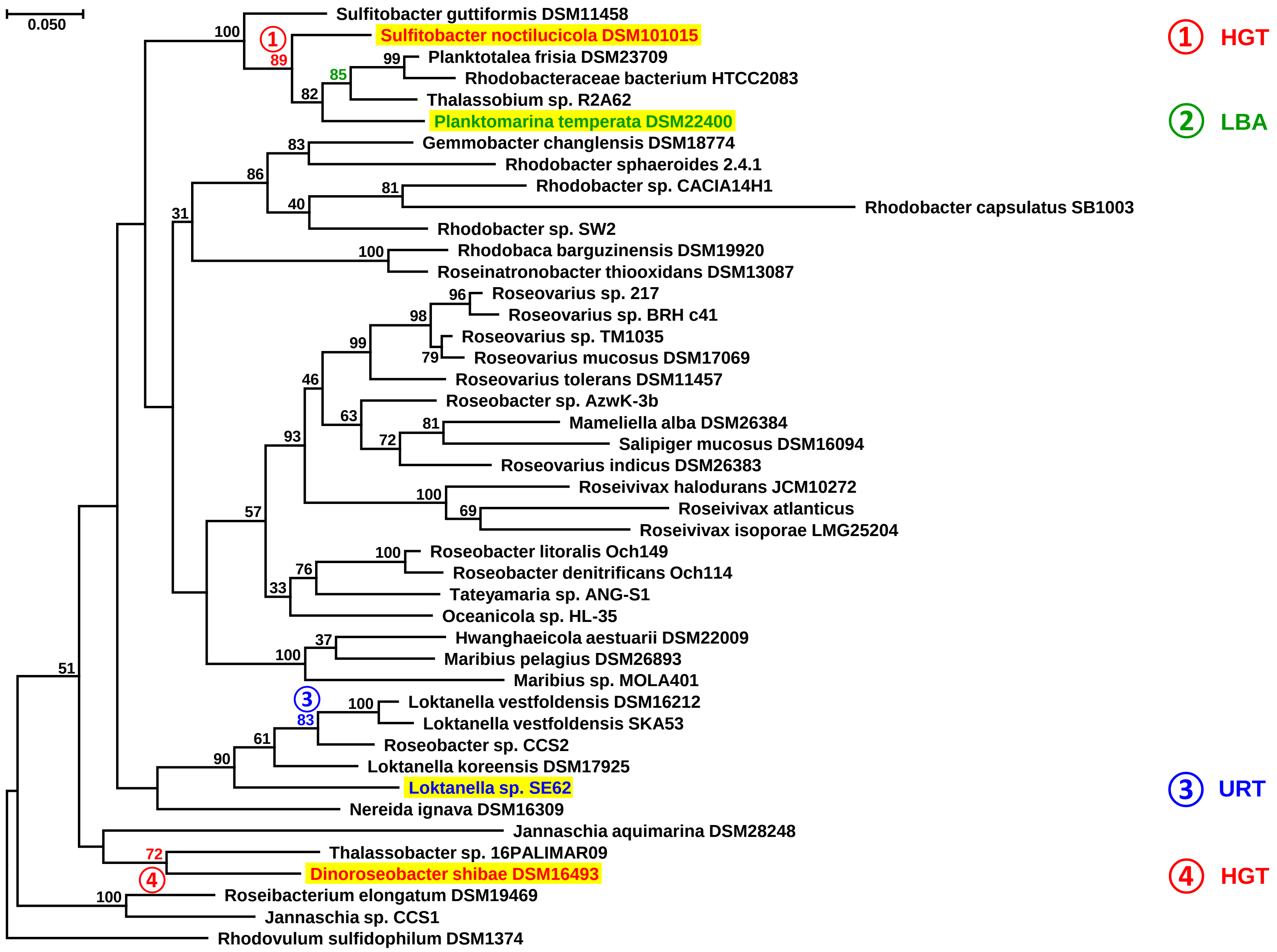

Figure S3-26 - bchH #39

Phylogenetic RaxML Analysis (LGF4Γ; 100 bootstrap replicates)

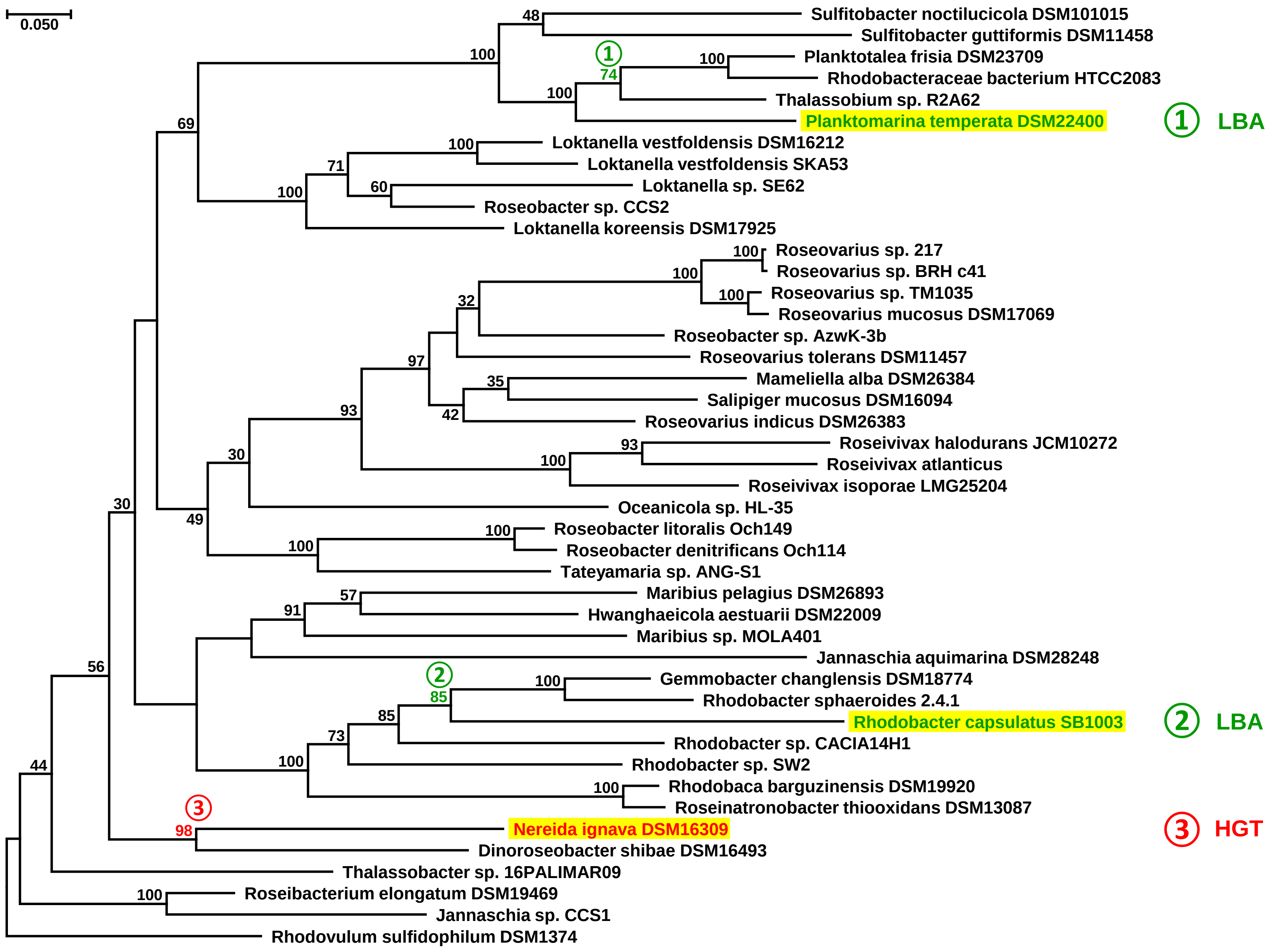

Figure S3-27 - bchL #40

Phylogenetic RaxML Analysis (LGF4Γ; 100 bootstrap replicates)

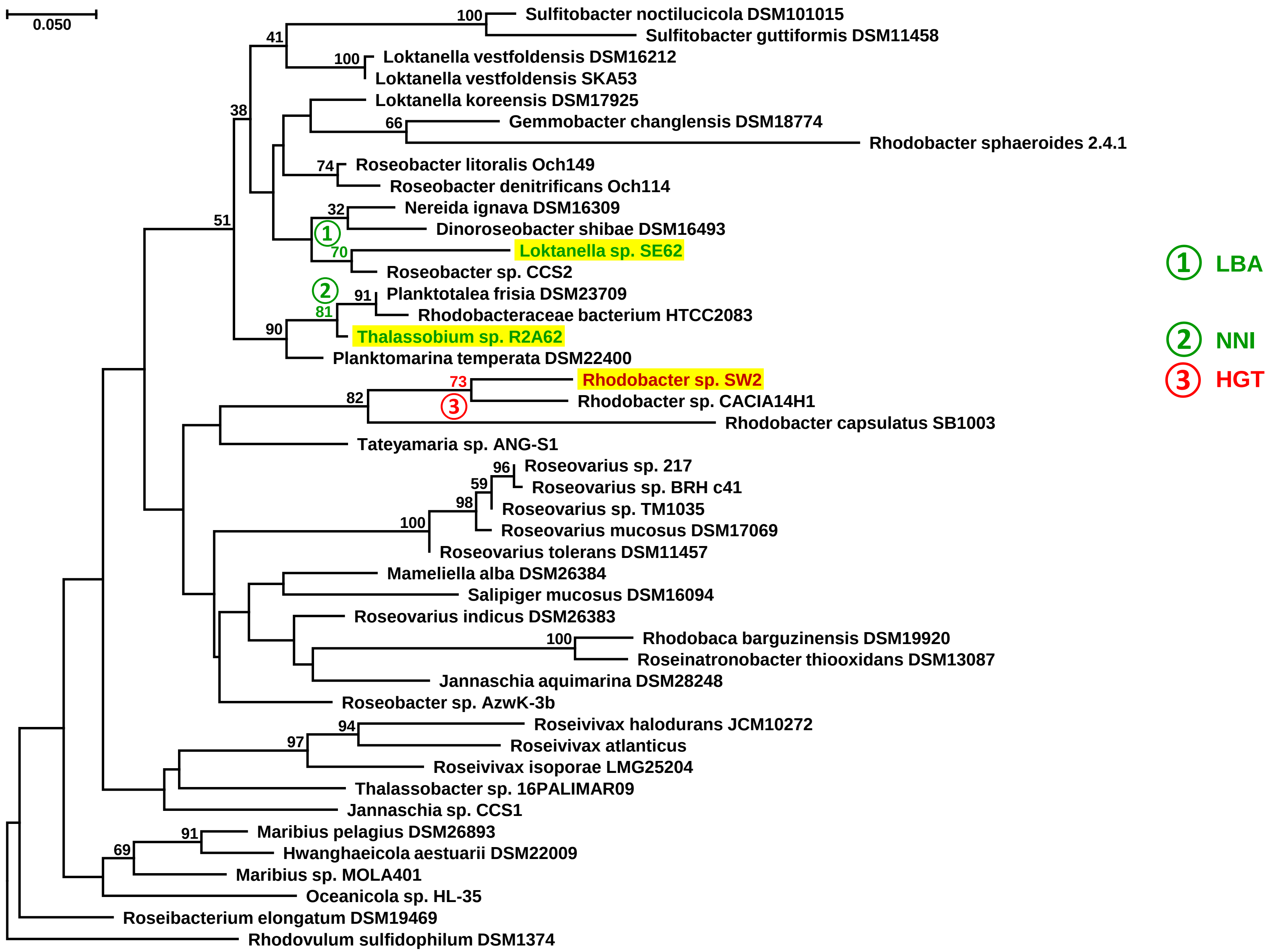

Figure S3-28 - bchM #41 Phylogenetic RaxML Analysis (LGF4Γ; 100 bootstrap replicates)

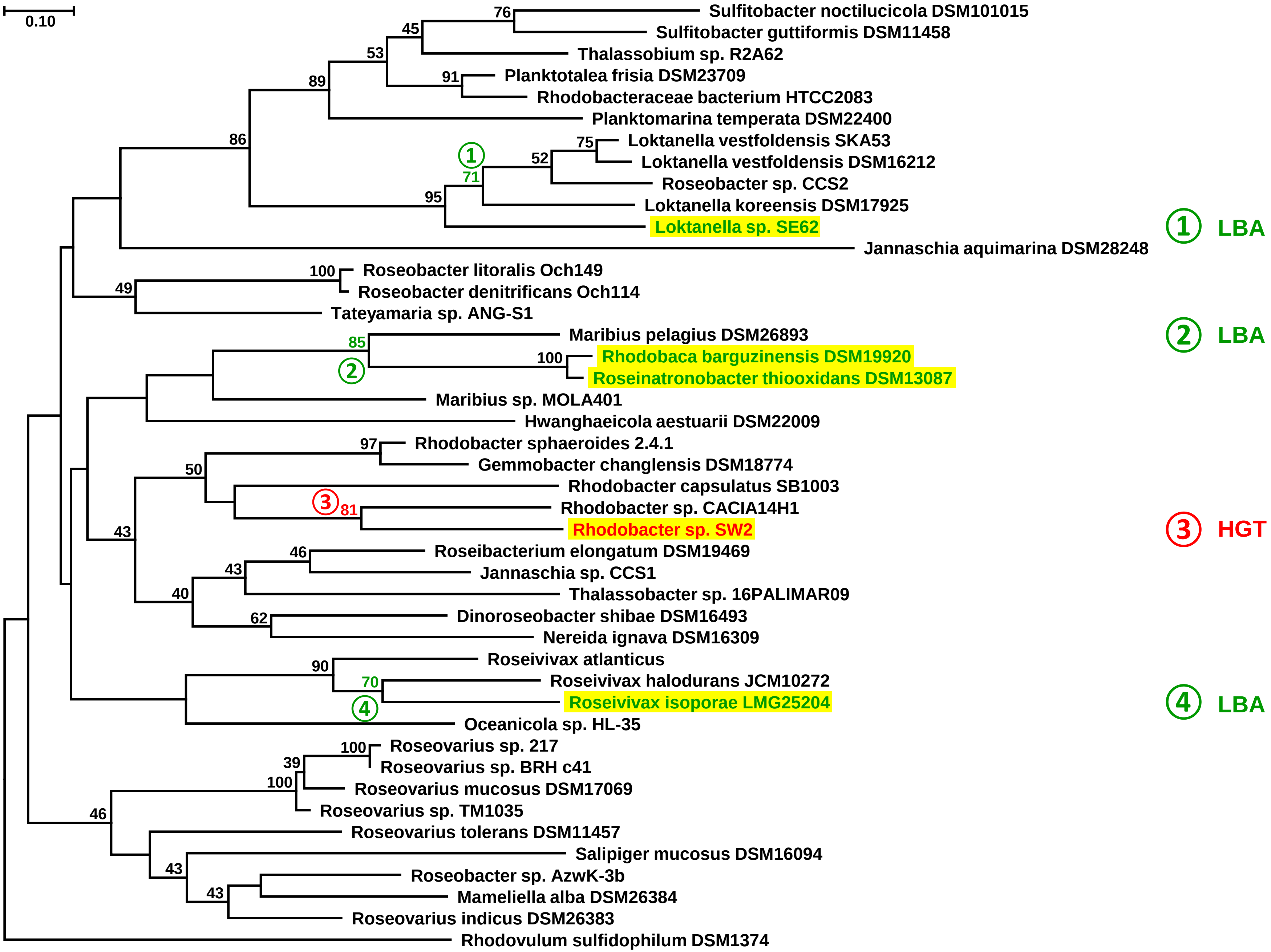

Figure S3-29 - lhaA #42

Phylogenetic RaxML Analysis (LGF4Γ; 100 bootstrap replicates)

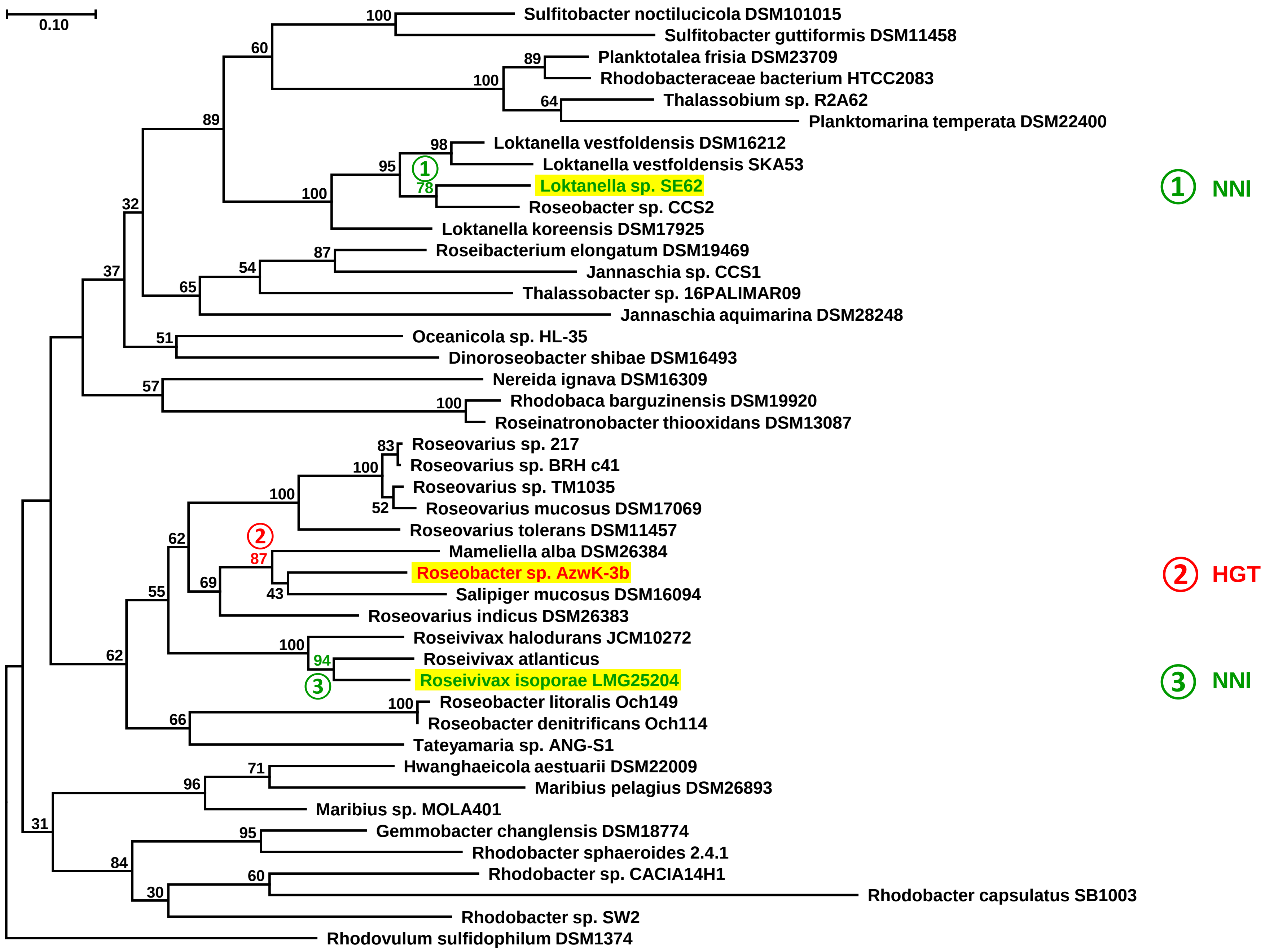

Figure S3-30 - puhA #43

Phylogenetic RaxML Analysis (LGF4Γ; 100 bootstrap replicates)

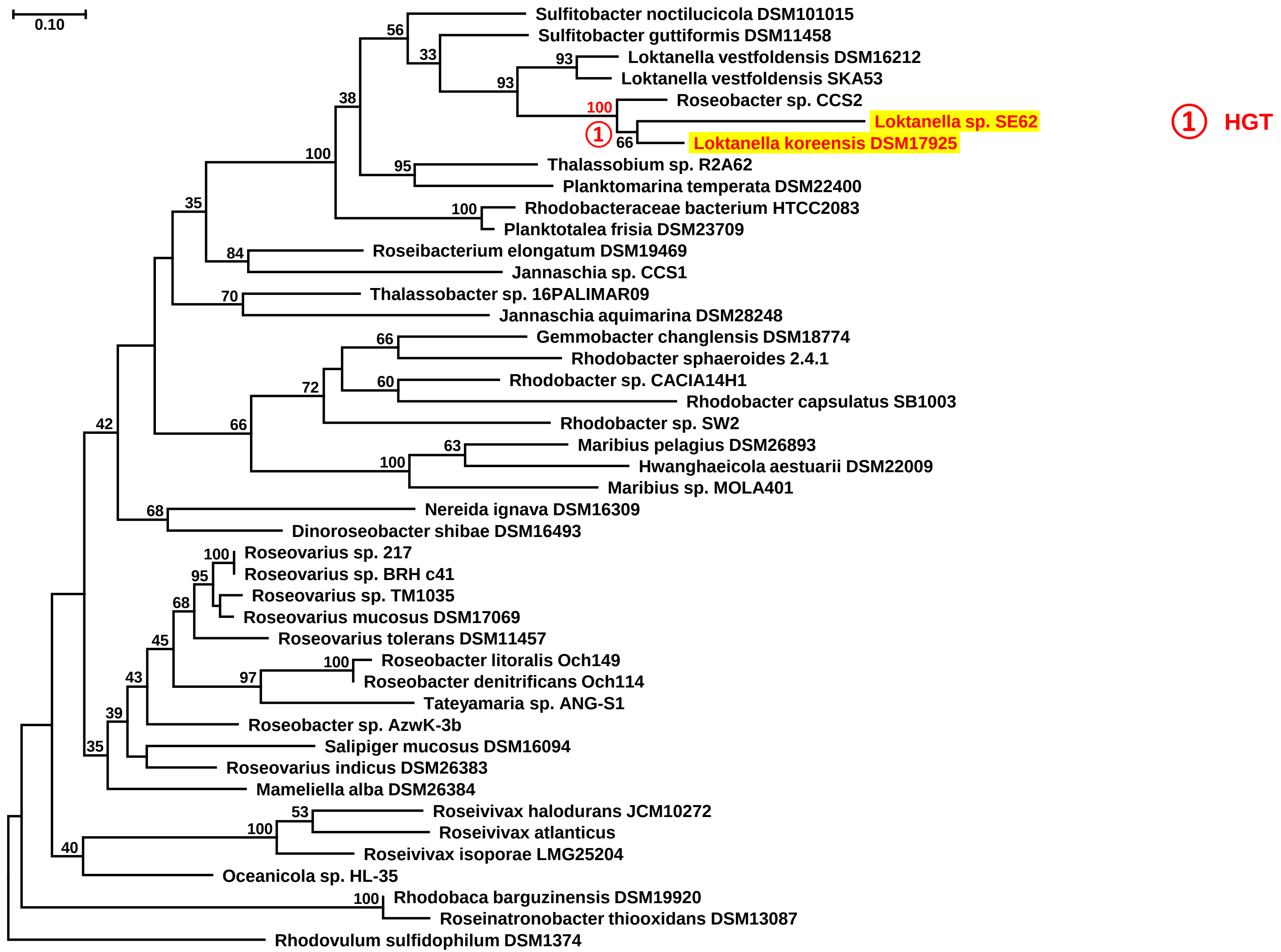

Figure S3-31 - puhB #44 Phylogenetic RaxML Analysis (LGF4Γ; 100 bootstrap replicates)

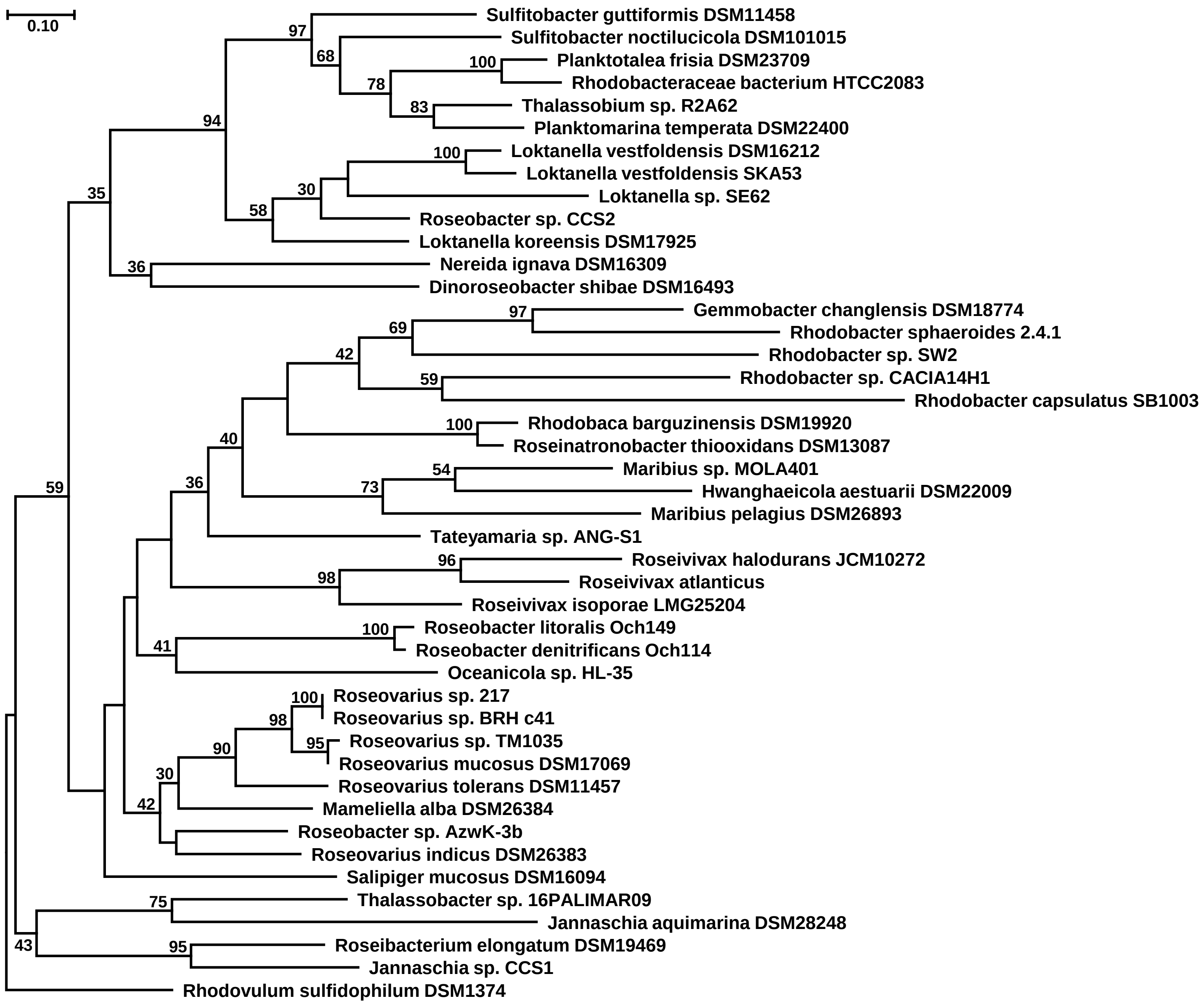

Figure S3-32 - acsF #47

Phylogenetic RaxML Analysis (LGF4Γ; 100 bootstrap replicates)

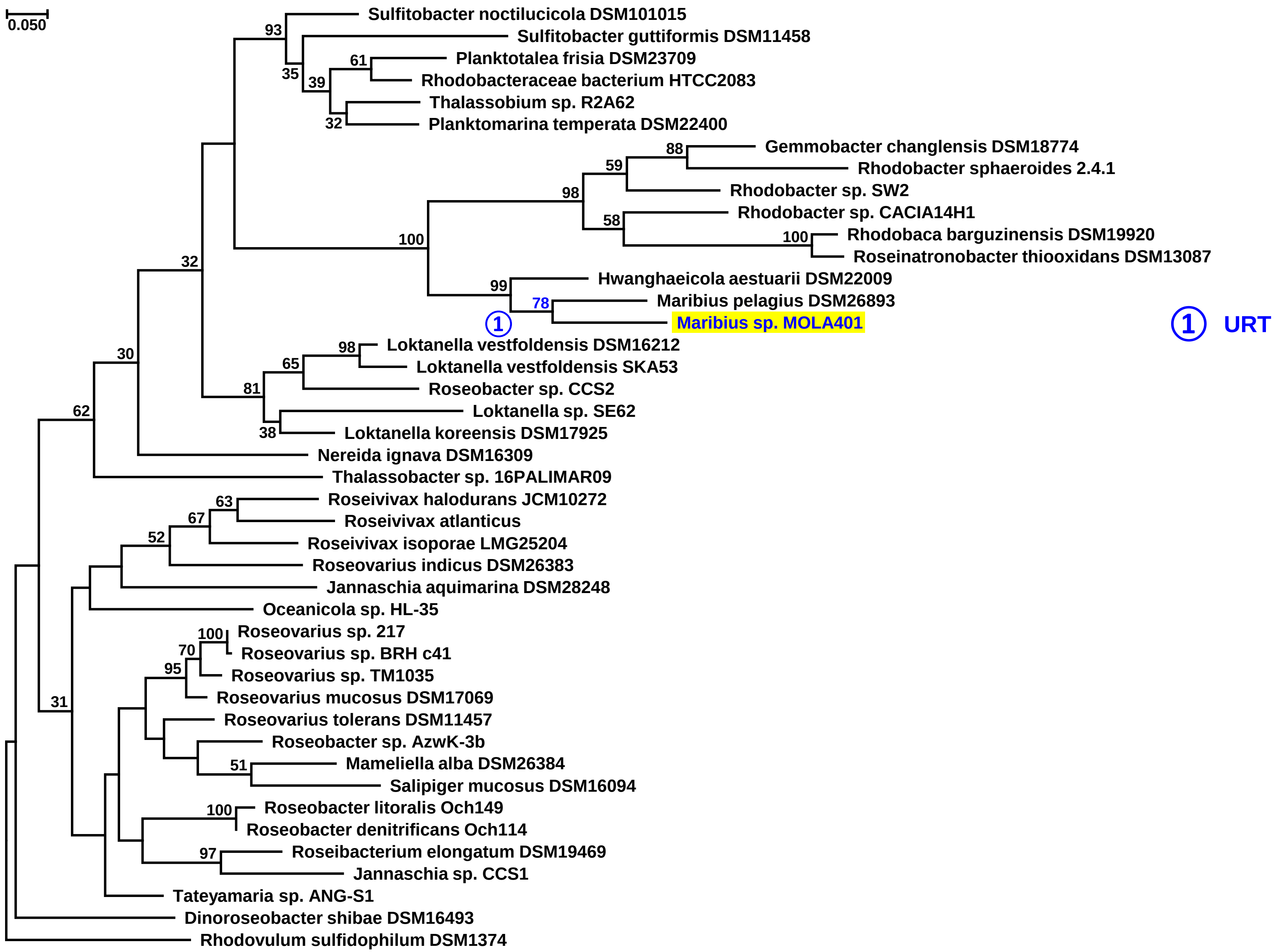

Figure S3-33 - puhE #48

Phylogenetic RaxML Analysis (LGF4Γ; 100 bootstrap replicates)

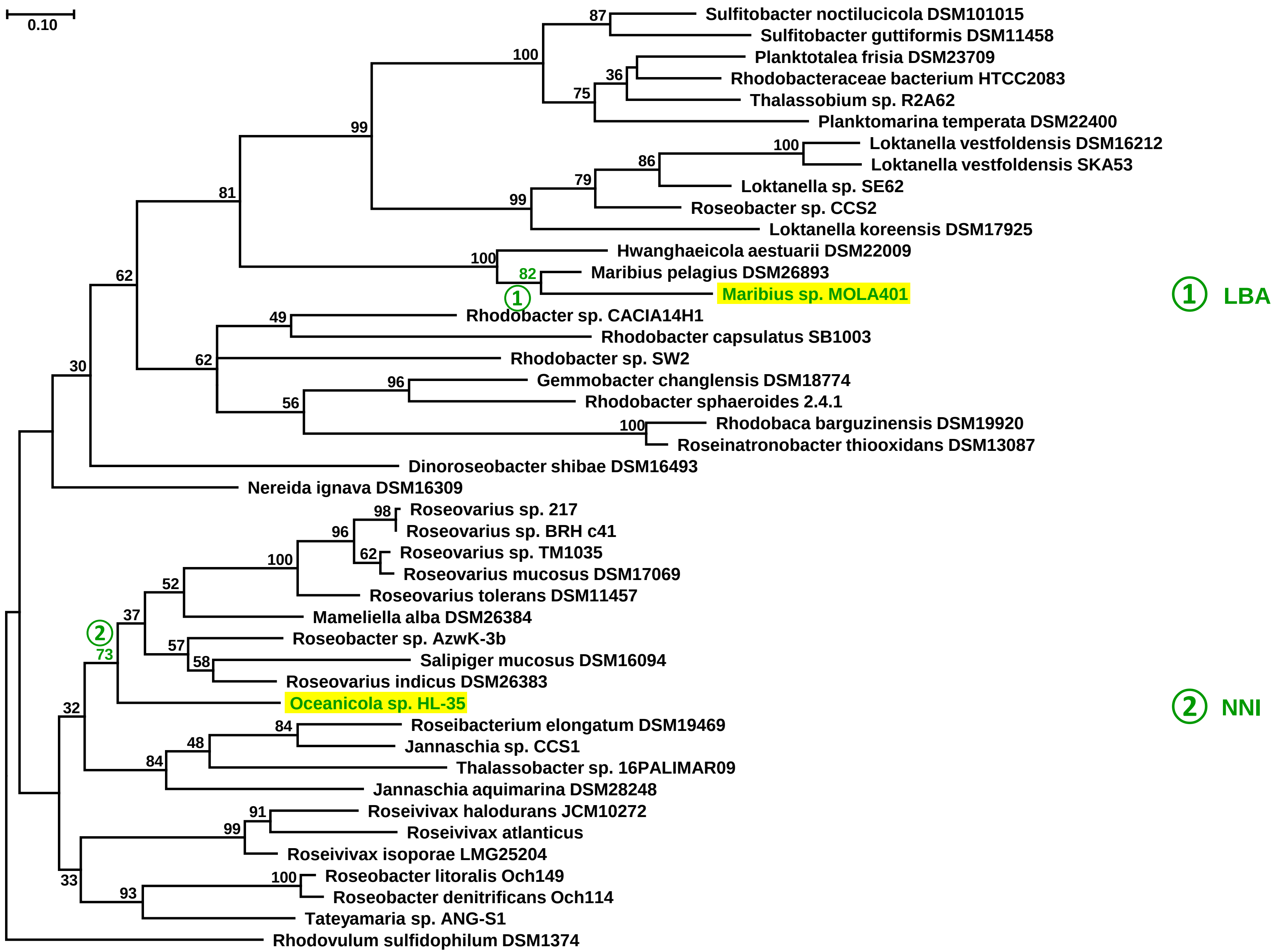

Figures S3-01 to **S3-33.** Single gene analyses of all 33 proteins that were used for the concatenated PGC tree. The phylogenetic analyses were performed with RAxML (LG+F+4Γ) including 100 rapid bootstrap replicates. The 33 trees are shown according to the gene order and numbering of the reference PGC from *Dinoroseobacter shibae* (see Fig. S1), which is exemplified by the largest marker gene *bchH* with the number #39 (Fig. S3-26). The comparison of the individual phylogenies with the concatenated PGC tree (Fig. S2B) allowed to detect conflicts, which are shown if the altered topology is supported by a BP of at least 70%. These conflicts could either reflect (1) tree reconstruction artifacts (shown in green) or (2) genuine HGTs (shown in red). In order to distinguish between both alternatives the program Treefix was applied to all 33 trees at the significance level of p=0.05. The 'fixed' topological differences – reflecting reconstruction artifacts - were assigned to (i) nearest-neighbor interchanges (NNIs), (ii) long-branch attraction artifacts (LBAs) or (iii) not further determined (n.d.) reconstruction problems such as compositional bias or heterotachy. Conflicts that persisted after Treefix were considered as genuine HGTs, if the corresponding node in the concatenated PGC tree was supported with at least 90% BP. All conflicts with a lower BP were classified as 'uncertainties in the reference tree' (URTs; shown in blue). A total of 34 conflicts were classified as genuine HGTs and the respective sequences were removed from the alignment of the PGC-tree shown in Figure 2B; the summary of all conflicts is shown in Table S2.
