# Supplementary material for: Horizontal operon transfer, plasmids, and the evolution of photosynthesis in Rhodobacteraceae
Source: ISME J. 2018 May 24;12(8):1994–2010. doi: 10.1038/s41396-018-0150-9 (PMC6052148; doi:10.1038/s41396-018-0150-9)
Supplement: Supplementary file 18 — Figure S5 [file 41396_2018_150_MOESM18_ESM.pdf]

Figure S5

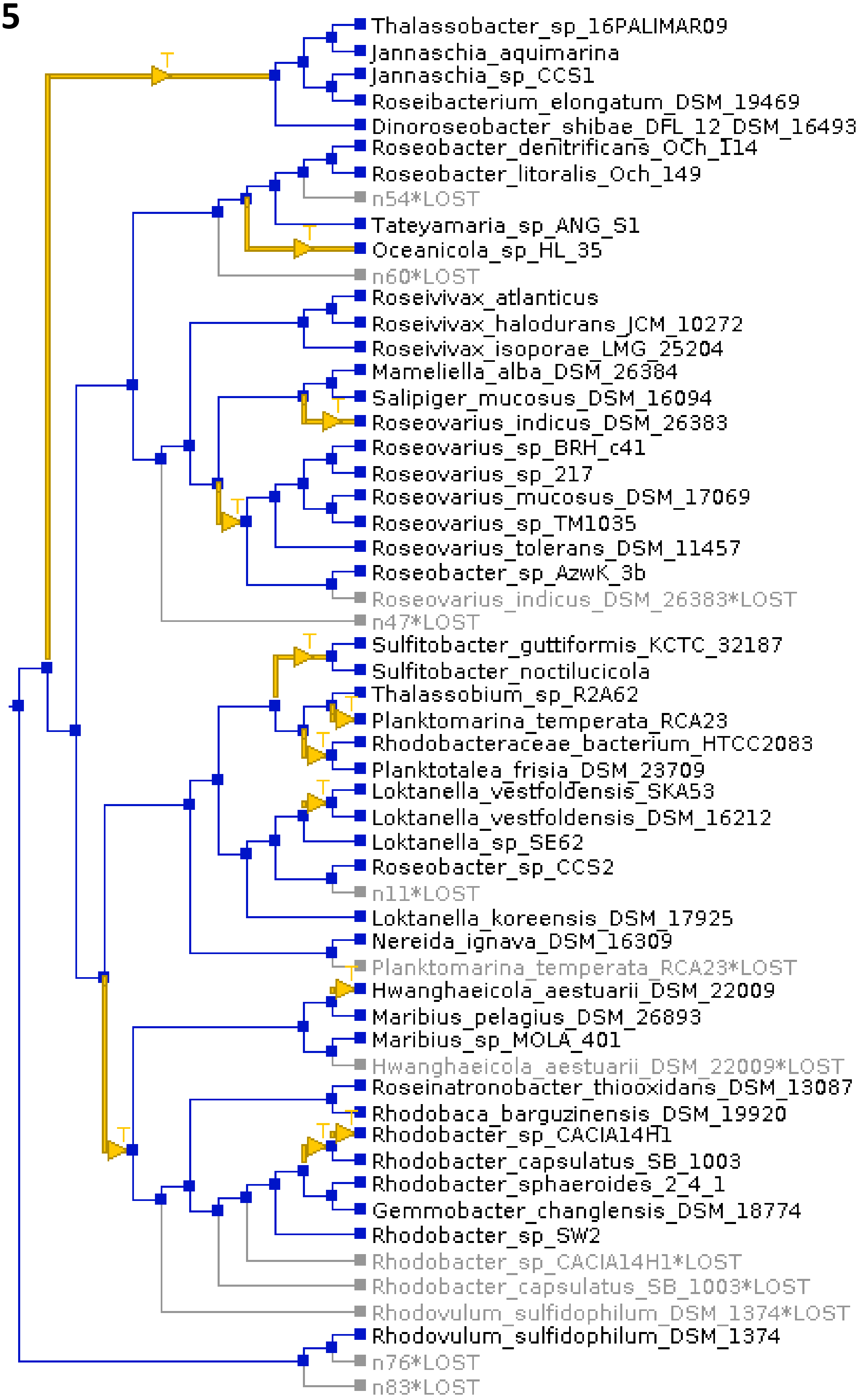

Figure S5A. Notung analysis showing one of 64 optimal solutions for the reconciliation of the PGC tree (Fig. S2B) of *Rhodobacteraceae* with the species tree (Fig. 2A). Horizontal transfers (T) of the PGC are shown by yellow arrows. PGC losses are indicated in grey (*LOST).

Figure S5

Statistics for PGC-tree without treefix (Fig. S2B):

Reconciliation Information
- Duplications: 0
- Co-Divergences: 0
- Transfers: **12**
- Losses: **12**
- Number of Temporally Feasible Optimal Solutions: **64**

Figure S5. Statistics of the Notung analysis for the reconciliation of the PGC tree (Fig. S2B) of *Rhodobacteraceae* with the species tree (Fig. 2A).
