# Supplementary material for: Horizontal operon transfer, plasmids, and the evolution of photosynthesis in Rhodobacteraceae
Source: ISME J. 2018 May 24;12(8):1994–2010. doi: 10.1038/s41396-018-0150-9 (PMC6052148; doi:10.1038/s41396-018-0150-9)
Supplement: Supplementary file 19 — Figure S6 [file 41396_2018_150_MOESM19_ESM.pdf]

Figure S6-01

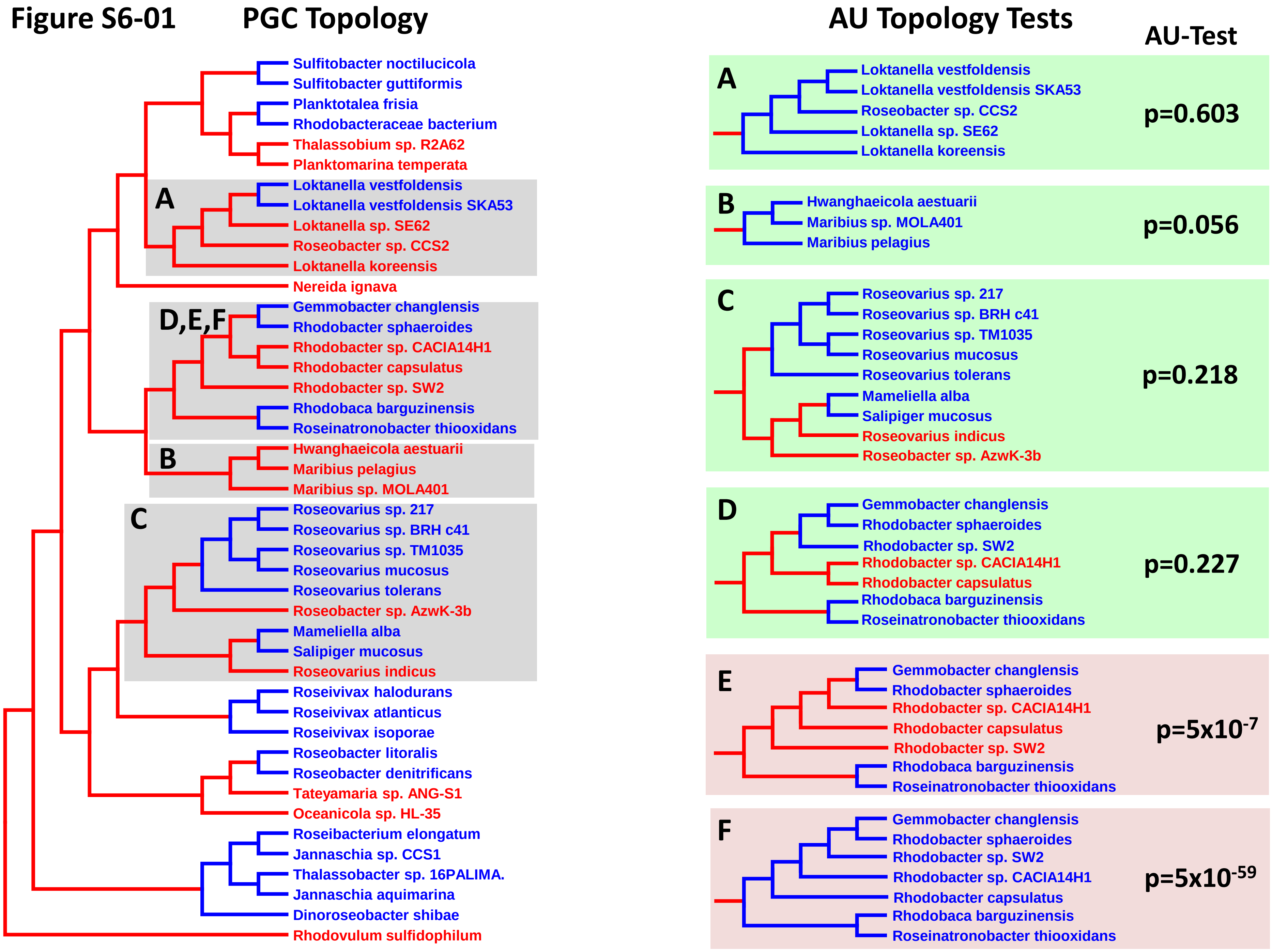
PGC Topology
Sulfitobacter noctilucicola
Sulfitobacter guttiformis
Planktotalea frisia
Rhodobacteraceae bacterium
Thalassobium sp. R2A62
Planktomarina temperata
A
Loktanella vestfoldensis
Loktanella vestfoldensis SKA53
Loktanella sp. SE62
Roseobacter sp. CCS2
Loktanella koreensis
Nereida ignava
D,E,F
Gemmobacter changlensis
Rhodobacter sphaeroides
Rhodobacter sp. CACIA14H1
Rhodobacter capsulatus
Rhodobacter sp. SW2
Rhodobaca barguzinensis
Roseinatronobacter thiooxidans
B
Hwanghaeicola aestuarii
Maribius pelagius
Maribius sp. MOLA401
C
Roseovarius sp. 217
Roseovarius sp. BRH c41
Roseovarius sp. TM1035
Roseovarius mucosus
Roseovarius tolerans
Roseobacter sp. AzwK-3b
Mameliella alba
Salipiger mucosus
Roseovarius indicus
Roseivivax halodurans
Roseivivax atlanticus
Roseivivax isoporae
Roseobacter litoralis
Roseobacter denitrificans
Tateyamaria sp. ANG-S1
Oceanicola sp. HL-35
Roseibacterium elongatum
Jannaschia sp. CCS1
Thalassobacter sp. 16PALIMA.
Jannaschia aquimarina
Dinoroseobacter shibae
Rhodovulum sulfidophilum
AU Topology Tests
AU-Test
A
Loktanella vestfoldensis
Loktanella vestfoldensis SKA53
Roseobacter sp. CCS2
Loktanella sp. SE62
Loktanella koreensis
p=0.603
B
Hwanghaeicola aestuarii
Maribius sp. MOLA401
Maribius pelagius
p=0.056
C
Roseovarius sp. 217
Roseovarius sp. BRH c41
Roseovarius sp. TM1035
Roseovarius mucosus
Roseovarius tolerans
Mameliella alba
Salipiger mucosus
Roseovarius indicus
Roseobacter sp. AzwK-3b
p=0.218
D
Gemmobacter changlensis
Rhodobacter sphaeroides
Rhodobacter sp. SW2
Rhodobacter sp. CACIA14H1
Rhodobacter capsulatus
Rhodobaca barguzinensis
Roseinatronobacter thiooxidans
p=0.227
E
Gemmobacter changlensis
Rhodobacter sphaeroides
Rhodobacter sp. CACIA14H1
Rhodobacter capsulatus
Rhodobacter sp. SW2
Rhodobaca barguzinensis
Roseinatronobacter thiooxidans
$p=5x10^{-7}$
F
Gemmobacter changlensis
Rhodobacter sphaeroides
Rhodobacter sp. SW2
Rhodobacter sp. CACIA14H1
Rhodobacter capsulatus
Rhodobaca barguzinensis
Roseinatronobacter thiooxidans
$p=5x10^{-59}$

Figure S6-02 **PGC Topology** **AU Topology Tests** **AU-Test**

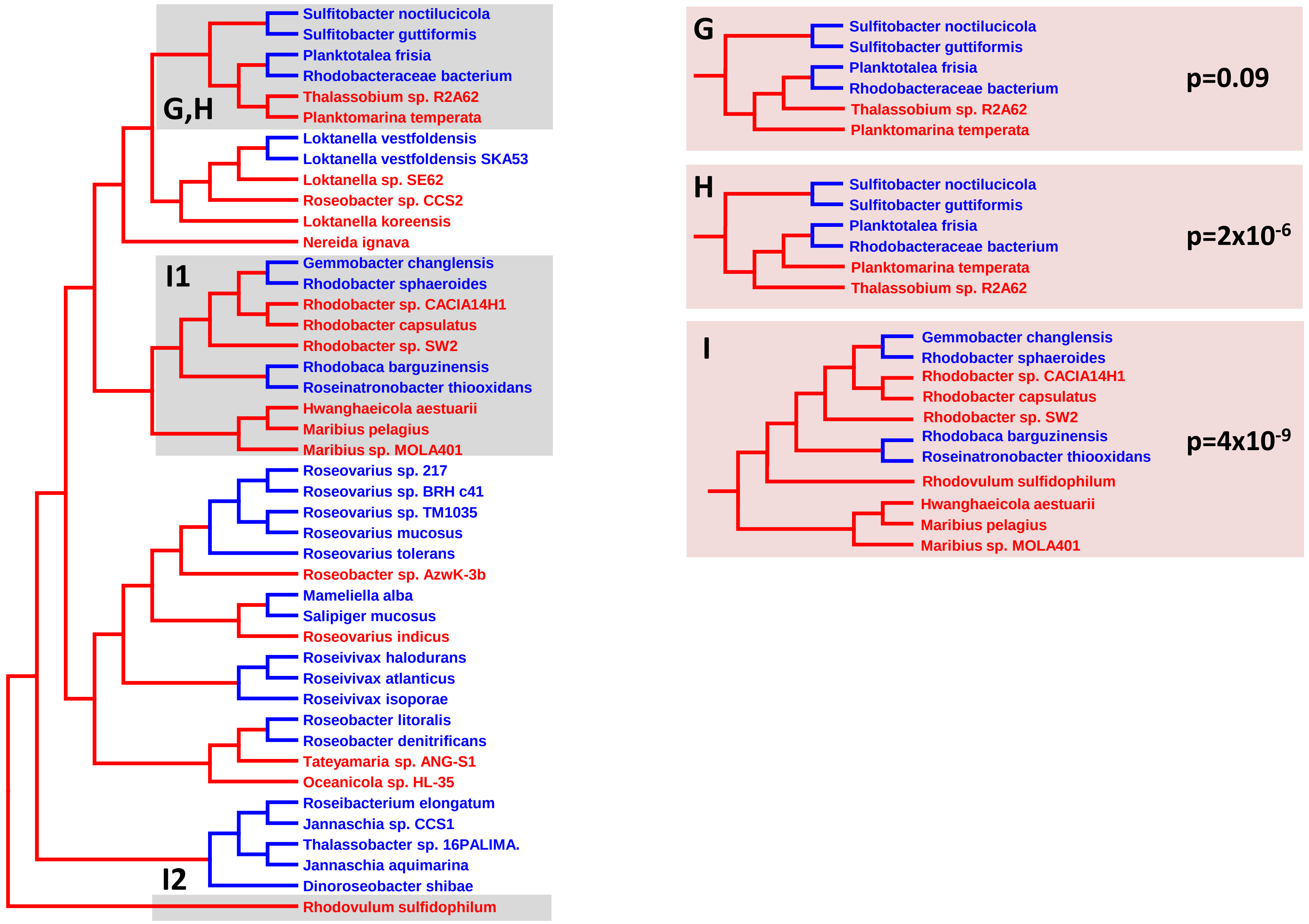

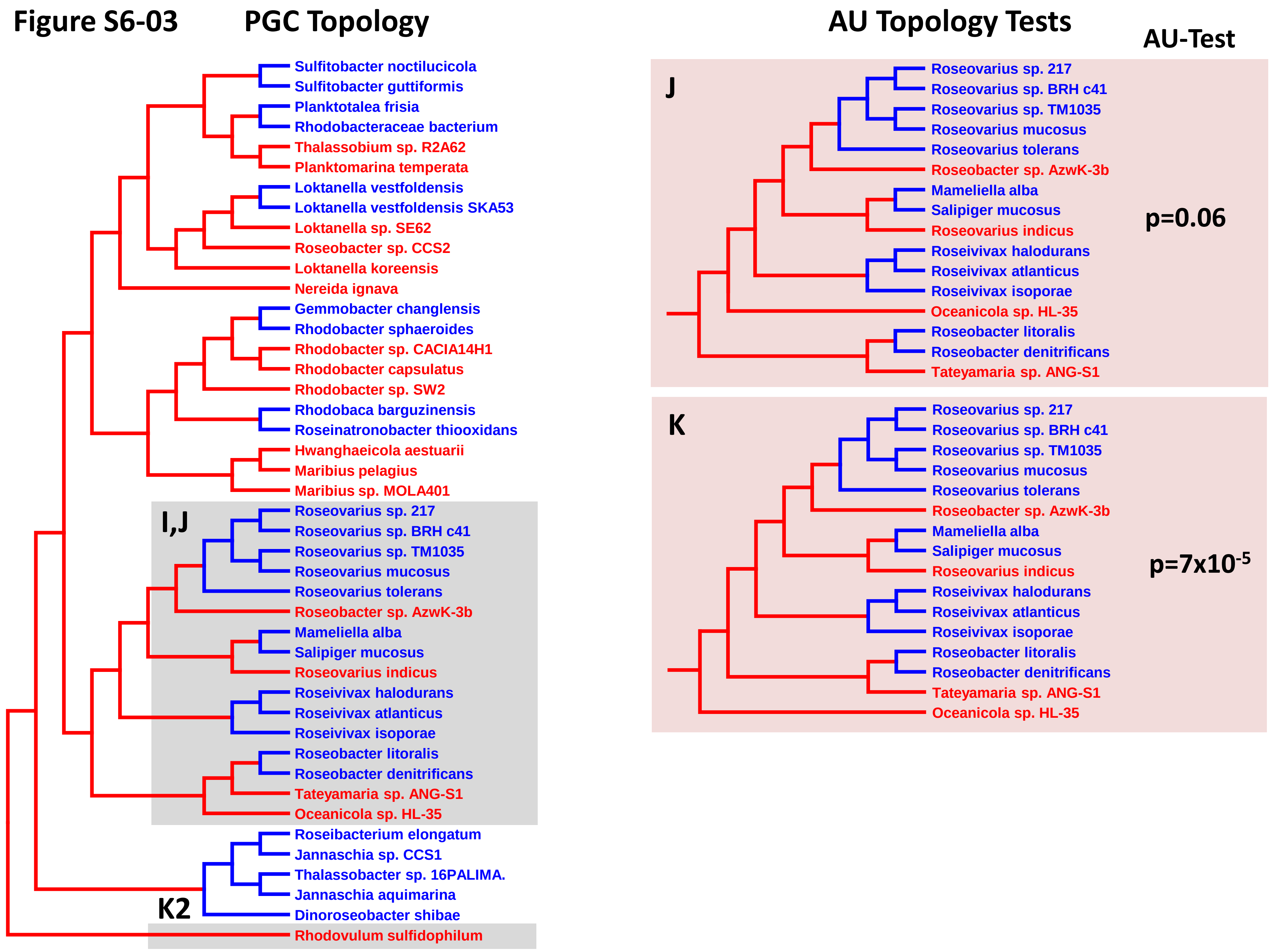
Figure S6-03
PGC Topology
AU Topology Tests
AU-Test
Sulfitobacter noctilucicola
Sulfitobacter guttiformis
Planktotalea frisia
Rhodobacteraceae bacterium
Thalassobium sp. R2A62
Planktomarina temperata
Loktanella vestfoldensis
Loktanella vestfoldensis SKA53
Loktanella sp. SE62
Roseobacter sp. CCS2
Loktanella koreensis
Nereida ignava
Gemmobacter changlensis
Rhodobacter sphaeroides
Rhodobacter sp. CACIA14H1
Rhodobacter capsulatus
Rhodobacter sp. SW2
Rhodobaca barguzinensis
Roseinatronobacter thiooxidans
Hwanghaeicola aestuarii
Maribius pelagius
Maribius sp. MOLA401
I,J
Roseovarius sp. 217
Roseovarius sp. BRH c41
Roseovarius sp. TM1035
Roseovarius mucosus
Roseovarius tolerans
Roseobacter sp. AzwK-3b
Mameliella alba
Salipiger mucosus
Roseovarius indicus
Roseivivax halodurans
Roseivivax atlanticus
Roseivivax isoporae
Roseobacter litoralis
Roseobacter denitrificans
Tateyamaria sp. ANG-S1
Oceanicola sp. HL-35
Roseibacterium elongatum
Jannaschia sp. CCS1
Thalassobacter sp. 16PALIMA.
Jannaschia aquimarina
K2
Dinoroseobacter shibae
Rhodovulum sulfidophilum
J
Roseovarius sp. 217
Roseovarius sp. BRH c41
Roseovarius sp. TM1035
Roseovarius mucosus
Roseovarius tolerans
Roseobacter sp. AzwK-3b
Mameliella alba
Salipiger mucosus
Roseovarius indicus
Roseivivax halodurans
Roseivivax atlanticus
Roseivivax isoporae
Oceanicola sp. HL-35
Roseobacter litoralis
Roseobacter denitrificans
Tateyamaria sp. ANG-S1
p=0.06
K
Roseovarius sp. 217
Roseovarius sp. BRH c41
Roseovarius sp. TM1035
Roseovarius mucosus
Roseovarius tolerans
Roseobacter sp. AzwK-3b
Mameliella alba
Salipiger mucosus
Roseovarius indicus
Roseivivax halodurans
Roseivivax atlanticus
Roseivivax isoporae
Roseobacter litoralis
Roseobacter denitrificans
Tateyamaria sp. ANG-S1
Oceanicola sp. HL-35
p=7x10-5

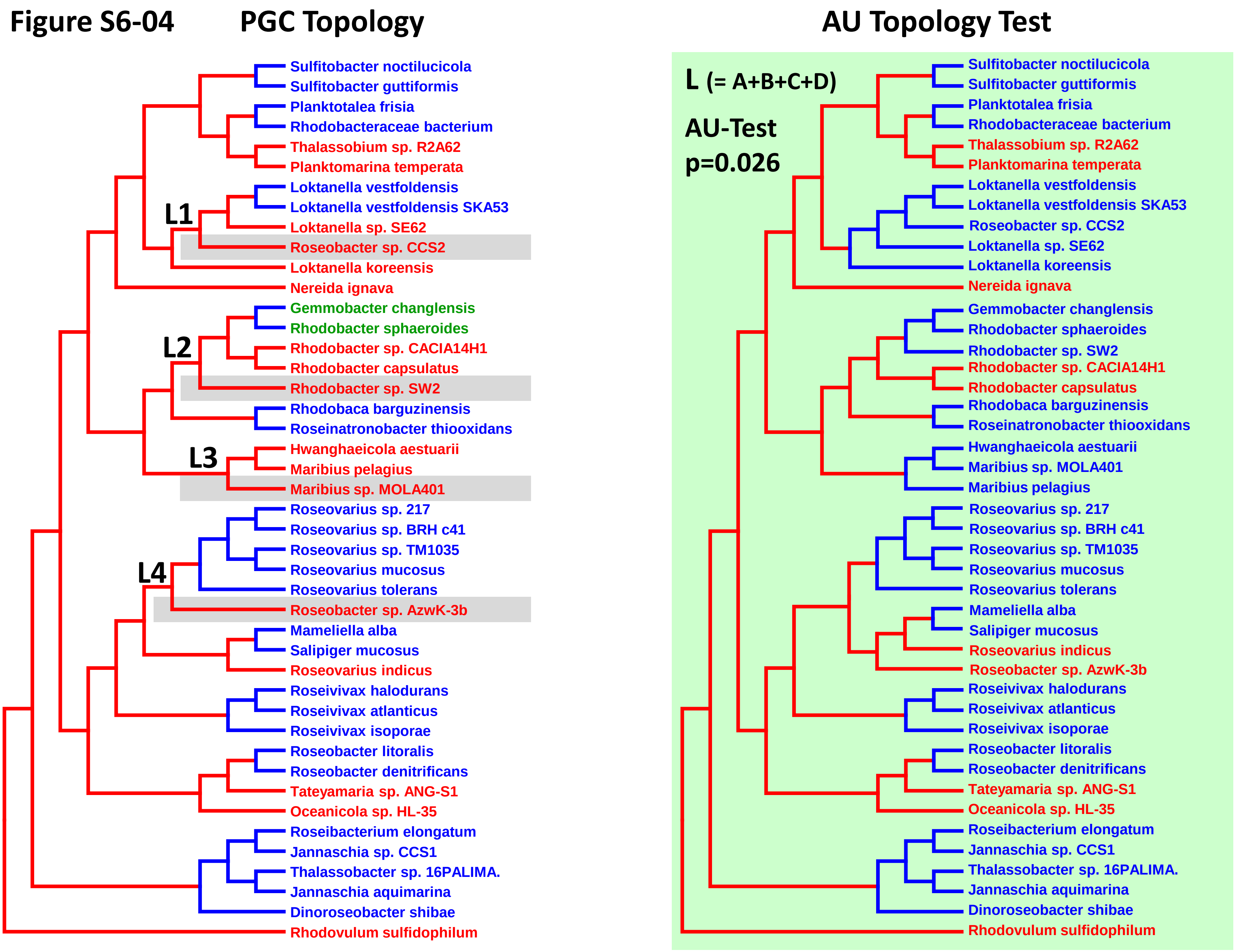
PGC Topology
AU Topology Test
Sulfitobacter noctilucicola
Sulfitobacter guttiformis
Planktotalea frisia
Rhodobacteraceae bacterium
Thalassobium sp. R2A62
Planktomarina temperata
Loktanella vestfoldensis
Loktanella vestfoldensis SKA53
Loktanella sp. SE62
L1
Roseobacter sp. CCS2
Loktanella koreensis
Nereida ignava
Gemmobacter changlensis
Rhodobacter sphaeroides
L2
Rhodobacter sp. CACIA14H1
Rhodobacter capsulatus
Rhodobacter sp. SW2
Rhodobaca barguzinensis
Roseinatronobacter thiooxidans
L3
Hwanghaeicola aestuarii
Maribius pelagius
Maribius sp. MOLA401
Roseovarius sp. 217
Roseovarius sp. BRH c41
Roseovarius sp. TM1035
Roseovarius mucosus
L4
Roseovarius tolerans
Roseobacter sp. AzwK-3b
Mameliella alba
Salipiger mucosus
Roseovarius indicus
Roseivivax halodurans
Roseivivax atlanticus
Roseivivax isoporae
Roseobacter litoralis
Roseobacter denitrificans
Tateyamaria sp. ANG-S1
Oceanicola sp. HL-35
Roseibacterium elongatum
Jannaschia sp. CCS1
Thalassobacter sp. 16PALIMA.
Jannaschia aquimarina
Dinoroseobacter shibae
Rhodovulum sulfidophilum
L (= A+B+C+D)
AU-Test
p=0.026
Sulfitobacter noctilucicola
Sulfitobacter guttiformis
Planktotalea frisia
Rhodobacteraceae bacterium
Thalassobium sp. R2A62
Planktomarina temperata
Loktanella vestfoldensis
Loktanella vestfoldensis SKA53
Roseobacter sp. CCS2
Loktanella sp. SE62
Loktanella koreensis
Nereida ignava
Gemmobacter changlensis
Rhodobacter sphaeroides
Rhodobacter sp. SW2
Rhodobacter sp. CACIA14H1
Rhodobacter capsulatus
Rhodobaca barguzinensis
Roseinatronobacter thiooxidans
Hwanghaeicola aestuarii
Maribius sp. MOLA401
Maribius pelagius
Roseovarius sp. 217
Roseovarius sp. BRH c41
Roseovarius sp. TM1035
Roseovarius mucosus
Roseovarius tolerans
Mameliella alba
Salipiger mucosus
Roseovarius indicus
Roseobacter sp. AzwK-3b
Roseivivax halodurans
Roseivivax atlanticus
Roseivivax isoporae
Roseobacter litoralis
Roseobacter denitrificans
Tateyamaria sp. ANG-S1
Oceanicola sp. HL-35
Roseibacterium elongatum
Jannaschia sp. CCS1
Thalassobacter sp. 16PALIMA.
Jannaschia aquimarina
Dinoroseobacter shibae
Rhodovulum sulfidophilum

Figure S6-04

Figure S6-05 **Approximately Unbiased (AU) Tests of Alternative Topologies**

Legend: level of significance 99% (p<0.01); BP = Bootstrap Proportions

green text = alternative topology not-significantly rejected; red text = significant rejection

Test	ΔlnL	BP	AU	Figure
ML	-1.9	0.377	0.757	Fig. 2B
A	1.9	0.420	0.603	Fig. S5-01
B	64.1	0.010	0.056	Fig. S5-01
C	23.5	0.099	0.218	Fig. S5-01
D	28.9	0.084	0.227	Fig. S5-01
E	229.3	0.000	$5x10^{-07}$	Fig. S5-01
F	194.7	0.000	$5x10^{-59}$	Fig. S5-01
G	76.7	0.002	0.009	Fig. S5-02
H	127.1	0.000	$2x10^{-06}$	Fig. S5-02
I	364.7	0.000	$4x10^{-09}$	Fig. S5-02
J	69.5	0.002	0.006	Fig. S5-03
K	96.2	0.000	$7x10^{-05}$	Fig. S5-03
L	90.3	0.005	0.026	Fig. S5-04

Figure S6. The significance of 12 alternative topologies (A to L) is estimated with the approximately unbiased (AU) test based on the ML topology of the PGC tree. Alternative topologies that are above the chosen significance level of p=0.01 are accepted and thus highlighted by a green box, those below are rejected und shown by a red box. Note the last test topology L is still accepted, despite the fact that it corresponds to the simultaneous change of the first four tests (A, B, C and D). The highest rejection with a p value of $5x10^{-59}$ is associated with two HOT events in the genus *Rhodobacter* (test F).
