# Supplementary material for: Horizontal operon transfer, plasmids, and the evolution of photosynthesis in Rhodobacteraceae
Source: ISME J. 2018 May 24;12(8):1994–2010. doi: 10.1038/s41396-018-0150-9 (PMC6052148; doi:10.1038/s41396-018-0150-9)
Supplement: Supplementary file 21 — Figure S8 [file 41396_2018_150_MOESM21_ESM.pdf]

Figure S8

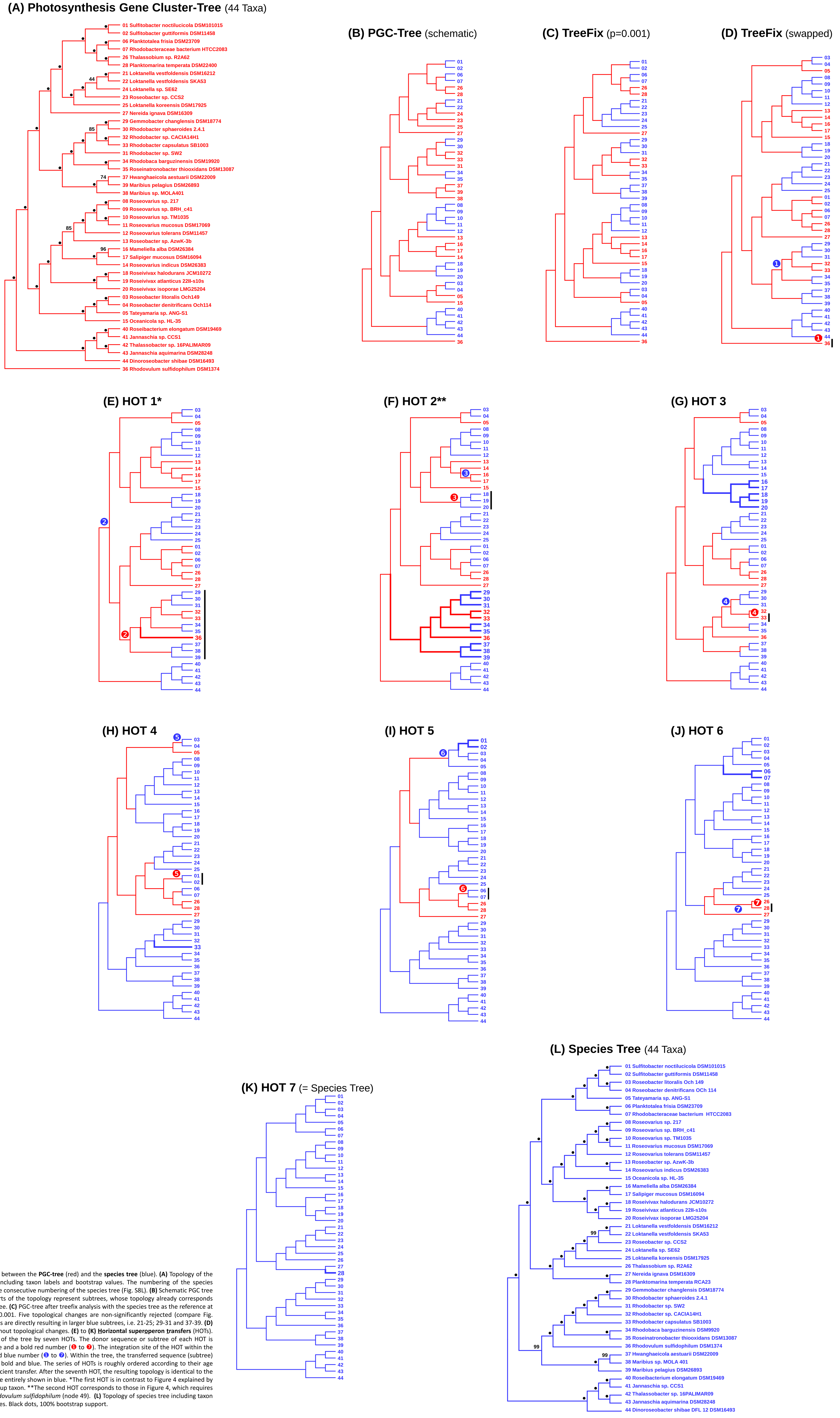

Figure S8. Reconciliation between the **PGC-tree** (red) and the **species tree** (blue). **(A)** Topology of the concatenated PGC tree including taxon labels and bootstrap values. The numbering of the species names corresponds to the consecutive numbering of the species tree (Fig. S8L). **(B)** Schematic PGC tree with numbers. Green parts of the topology represent subtrees, whose topology already corresponds to those of the species tree. **(C)** PGC-tree after treefix analysis with the species tree as the reference at the significance level p=0.001. Five topological changes are non-significantly rejected (compare Fig. S8B and C). Three changes are directly resulting in larger blue subtrees, i.e. 21-25; 29-31 and 37-39. **(D)** Swapped treefix tree without topological changes. **(E)** to **(K)** **Horizontal superoperon transfers** (HOTs). Stepwise transformation of the tree by seven HOTs. The donor sequence or subtree of each HOT is highlighted by a black line and a bold red number (❶ to ❼). The integration site of the HOT within the tree is indicated by a bold blue number (❶ to ❼). Within the tree, the transferred sequence (subtree) of each HOT is shown in bold and blue. The series of HOTs is roughly ordered according to their age starting with the most ancient transfer. After the seventh HOT, the resulting topology is identical to the species tree and therefore entirely shown in blue. *The first HOT is in contrast to Figure 4 explained by a transfer from an outgroup taxon. **The second HOT corresponds to those in Figure 4, which requires an additional loss of *Rhodovulum sulfidophilum* (node 49). **(L)** Topology of species tree including taxon labels and bootstrap values. Black dots, 100% bootstrap support.
