# Supplementary material for: Horizontal operon transfer, plasmids, and the evolution of photosynthesis in Rhodobacteraceae
Source: ISME J. 2018 May 24;12(8):1994–2010. doi: 10.1038/s41396-018-0150-9 (PMC6052148; doi:10.1038/s41396-018-0150-9)
Supplement: Supplementary file 22 — Figure S9 [file 41396_2018_150_MOESM22_ESM.pdf]

Figure S9 **RpoB-Phylogeny** (PhyloBayes; 91 ingroup strains; 1227 aa; CATGTR4Γ)

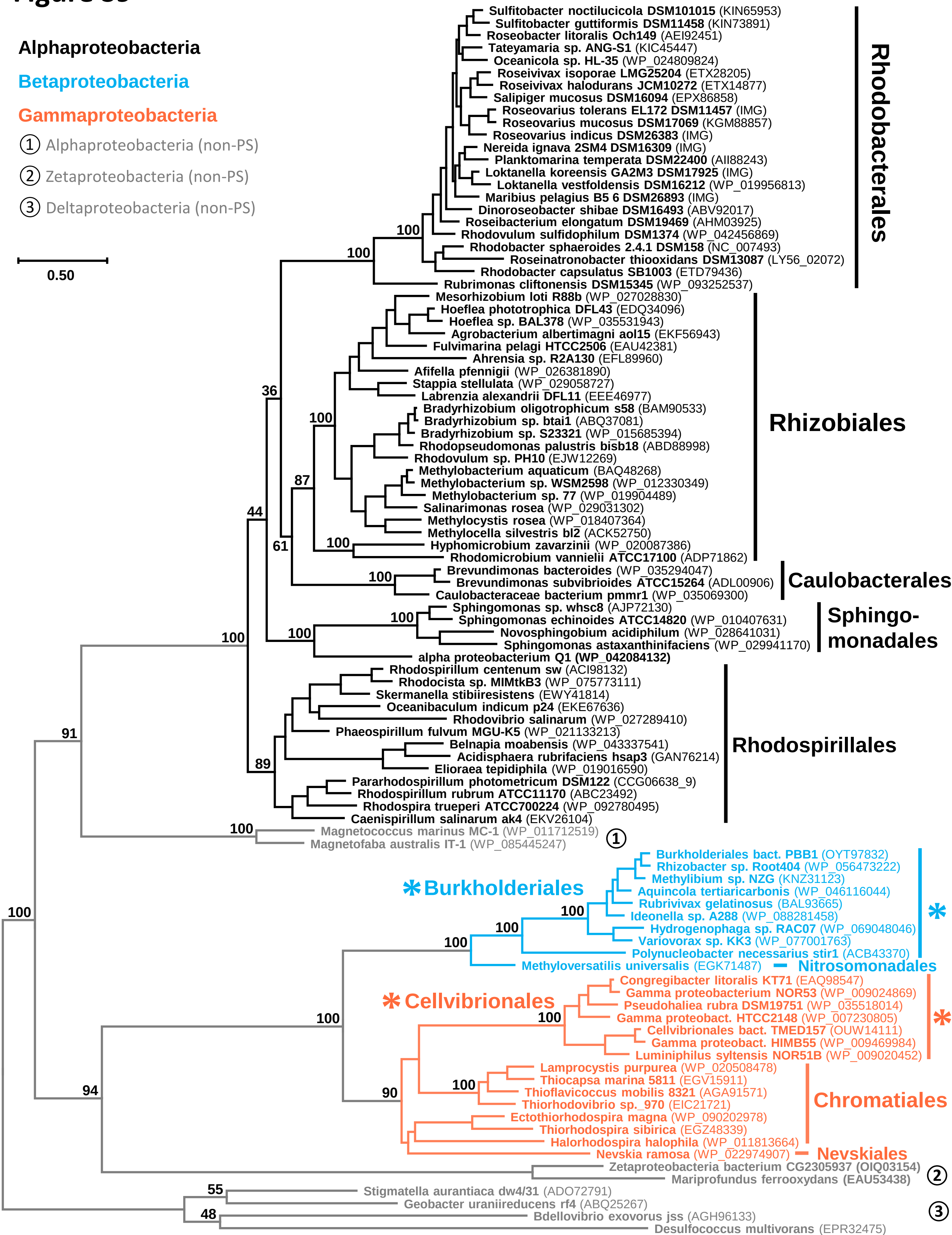

Figure S9. Phylogentic tree of the DNA-dependent RNA polymerase subunit B (RpoB). The tree was inferred with PhyloBayes under a CAT-GTR+4Γ model; selected bootstrap values generated in a RAxML analysis with a GTR+4Γ model are shown. A total of 99 RpoB sequences with 1,227 aligned amino acid positions after g-blocks were used for the phylogenetic inference. This included 91 ingroup *(Alpha-*, *Beta-*, *Gammaproteobacteria*) as well as 8 outgroup sequences (shown in grey). The taxonomic affiliation of the three classes *Alpha-*, *Beta-* and *Gammaproteobacteria* are shown at the order level, those of the outgroup sequences only at the class level. The tree clearly shows the long evolutionary time span of the three ingroup classes that passed by before the extant diversity originated.
