# Supplementary material for: Horizontal operon transfer, plasmids, and the evolution of photosynthesis in Rhodobacteraceae
Source: ISME J. 2018 May 24;12(8):1994–2010. doi: 10.1038/s41396-018-0150-9 (PMC6052148; doi:10.1038/s41396-018-0150-9)
Supplement: Supplementary file 23 — Figure S10 [file 41396_2018_150_MOESM23_ESM.pdf]

Figure S10

BchH-Phylogeny (PhyloBayes; 91 ingroup strains; 1062aa; CATGTR4Γ)

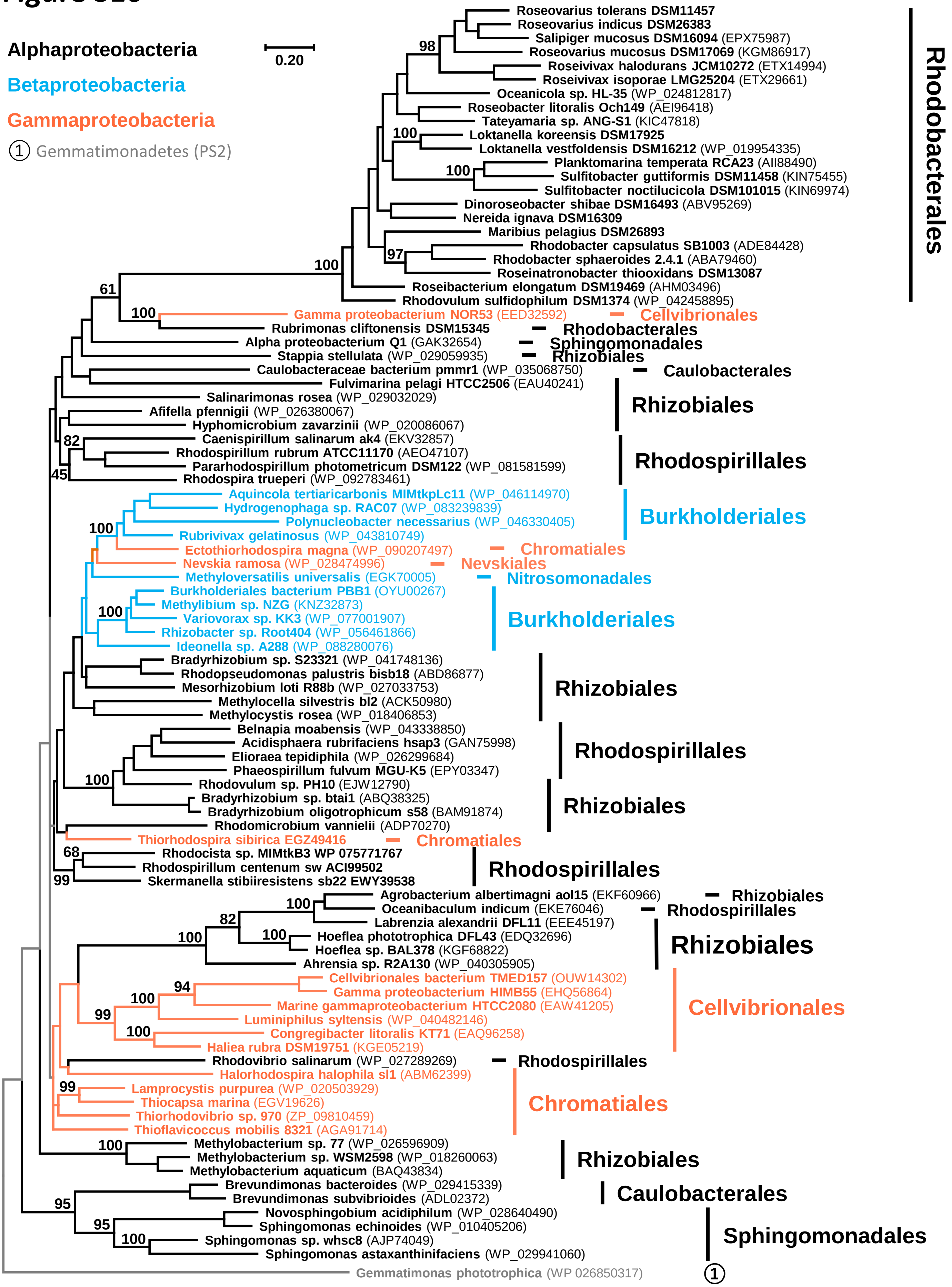

Figure S10. Phylogenetic tree of the subunit H of the magnesium chelatase (BchH). The tree was inferred with PhyloBayes under a CAT-GTR+4Γ model, selected bootstrap values generated in a RAxML analysis with a GTR+4Γ model are shown. A total of 92 BchH sequences with 1,062 aligned amino acid positions after g-blocks were used for the phylogenetic inference. This included 91 ingroup (*Alpha*-, *Beta*-, *Gammaproteobacteria*) as well as 1 outgroup sequence *Gemmatimonas phototrophica* (*Gemmatimonadetes*) that is shown in grey. The taxonomic affiliation of the three classes *Alpha*-, *Beta*- and *Gammaproteobacteria* are shown at the order level, those of the outgroup sequence at the class level. The figure shows a massive radiation with the *Rhodobacteraceae* (core *Rhodobacterales*) as the only remaining higher order monophyletic group. The coloration of the groups illustrates many putative HGTs between the three ingroup classes as well as among the taxa within a given class and even within all studied orders.
