# Supplementary material for: Horizontal operon transfer, plasmids, and the evolution of photosynthesis in Rhodobacteraceae
Source: ISME J. 2018 May 24;12(8):1994–2010. doi: 10.1038/s41396-018-0150-9 (PMC6052148; doi:10.1038/s41396-018-0150-9)
Supplement: Supplementary file 17 — Figure S4 [file 41396_2018_150_MOESM17_ESM.pdf]

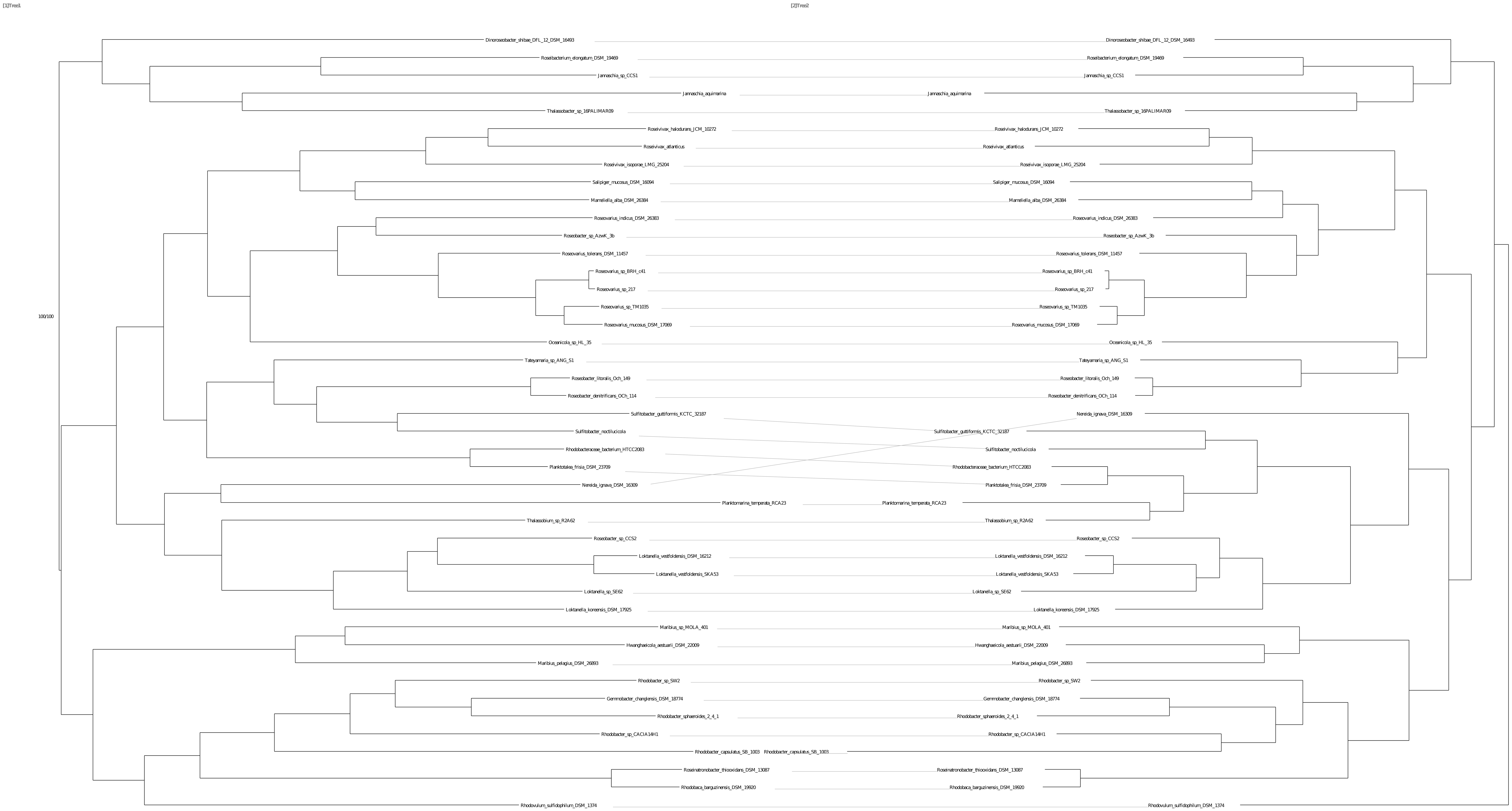

[1]Tree1
[2]Tree2
100/100
Dinoroseobacter_shibae_DFL_12_DSM_16493
Roseibacterium_elongatum_DSM_19469
Jannaschia_sp_CCS1
Jannaschia_aquimarina
Thalassobacter_sp_16PALIMAR09
Roseivivax_halodurans_JCM_10272
Roseivivax_atlanticus
Roseivivax_isoporae_LMG_25204
Salipiger_mucosus_DSM_16094
Marinellia_alba_DSM_26384
Roseovarius_indicus_DSM_26383
Roseobacter_sp_AzwK_3b
Roseovarius_tolerans_DSM_11457
Roseovarius_sp_BRH_c41
Roseovarius_sp_217
Roseovarius_sp_TM1035
Roseovarius_mucosus_DSM_17069
Oceanicola_sp_HL_35
Tateyamaria_sp_ANG_S1
Roseobacter_litoralis_Och_149
Roseobacter_denitrificans_OCh_114
Sulfitobacter_guttiformis_KCTC_32187
Sulfitobacter_noctilucicola
Rhodobacteraceae_bacterium_HTCC2083
Planktotalea_frisia_DSM_23709
Nereida_ignava_DSM_16309
Planktomarina_temperata_RCA23
Thalassobium_sp_R2A62
Roseobacter_sp_CCS2
Loktanella_vestfoldensis_DSM_16212
Loktanella_vestfoldensis_SKA53
Loktanella_sp_SE62
Loktanella_koreensis_DSM_17925
Maribius_sp_MOLA_401
Hwanghaeicola_aestuarii_DSM_22009
Maribius_pelagius_DSM_26893
Rhodobacter_sp_SW2
Gemmobacter_changlensis_DSM_18774
Rhodobacter_sphaeroides_2_4_1
Rhodobacter_sp_CACIA14H1
Rhodobacter_capsulatus_SB_1003
Roseinatronobacter_thiooxidans_DSM_13087
Rhodobaca_barguzinensis_DSM_19920
Rhodovulum_sulfidophilum_DSM_1374

Figure S4. Tanglegram of the species tree (Fig. 2A) and the PGC tree (Fig. 2B) resulting in a minimal number of crossing sections.
